# The *Arabidopsis* transcriptional regulator DPB3‐1 enhances heat stress tolerance without growth retardation in rice

**DOI:** 10.1111/pbi.12535

**Published:** 2016-02-03

**Authors:** Hikaru Sato, Daisuke Todaka, Madoka Kudo, Junya Mizoi, Satoshi Kidokoro, Yu Zhao, Kazuo Shinozaki, Kazuko Yamaguchi‐Shinozaki

**Affiliations:** ^1^ Graduate School of Agricultural and Life SciencesUniversity of TokyoTokyoJapan; ^2^ RIKEN Center for Sustainable Resource ScienceTsurumi‐kuYokohamaJapan; ^3^ Present address: RIKEN Center for Sustainable Resource ScienceTsukubaIbaraki305‐0074Japan

**Keywords:** DNA polymerase II subunit B3‐1/nuclear factor Y subunit C10, heat stress tolerance, *Oryza sativa*, transcriptional regulation, dehydration‐responsive element binding protein 2, microarray analysis

## Abstract

The enhancement of heat stress tolerance in crops is an important challenge for food security to facilitate adaptation to global warming. In *Arabidopsis thaliana*, the transcriptional regulator DNA polymerase II subunit B3‐1 (DPB3‐1)/nuclear factor Y subunit C10 (NF‐YC10) has been reported as a positive regulator of Dehydration‐responsive element binding protein 2A (DREB2A), and the overexpression of *DPB3‐1* enhances heat stress tolerance without growth retardation. Here, we show that DPB3‐1 interacts with DREB2A homologues in rice and soya bean. Transactivation analyses with *Arabidopsis* and rice mesophyll protoplasts indicate that DPB3‐1 and its rice homologue OsDPB3‐2 function as positive regulators of DREB2A homologues. Overexpression of *DPB3‐1* did not affect plant growth or yield in rice under nonstress conditions. Moreover, *DPB3‐1*‐overexpressing rice showed enhanced heat stress tolerance. Microarray analysis revealed that many heat stress‐inducible genes were up‐regulated in *DPB3‐1*‐overexpressing rice under heat stress conditions. However, the overexpression of *DPB3‐1* using a constitutive promoter had almost no effect on the expression of these genes under nonstress conditions. This may be because DPB3‐1 is a coactivator and thus lacks inherent transcriptional activity. We conclude that DPB3‐1, a coactivator that functions specifically under abiotic stress conditions, could be utilized to increase heat stress tolerance in crops without negative effects on vegetative and reproductive growth.

## Introduction

There is increasing evidence of global warming, and it has been reported that the increased temperature will have negative effects on crop yields during the 21st century (Intergovernmental Panel on Climate Change, Working Group II, 2014). Moreover, frequent extreme heat events triggered by global warming will cause severe damage to several crop species, particularly in tropical and temperate regions (Deryng *et al*., 2014; Lobell *et al*., 2011; Schlenker and Roberts, 2009). Adaptation to climate change is one of the most important challenges for crop production in many parts of the world. Many previous studies have reported the negative effects of heat stress on plant growth and reproduction at physiological and molecular levels. Heat stress generally decreases the water content in plant cells, which results in cell growth inhibition, cell division and cell expansion (Ashraf and Hafeez, 2004; Hasanuzzaman *et al*., 2013; Rodríguez *et al*., 2005). Severe heat stress damages leaf tips and margins, resulting in leaf desiccation and ultimately necrosis (Omae *et al*., 2012). Several studies have reported that high temperature (28–30 °C) induces stem elongation or leaf elevation in some plants, such as *Arabidopsis thaliana* and potato; this also results in a decrease in total biomass or yield (Foreman *et al*., 2011; Patel and Franklin, 2009; Singh *et al*., 2013).

Several recent papers have demonstrated a loss of crop yields in response to high temperature. In 2003, severe heat stress caused a loss of approximately 5 million tons of rice grain yields in China (Tian *et al*., 2009). In Europe, heat waves in the summer of 2003 greatly reduced the yield of a wide range of agricultural crops (Ciais *et al*., 2005). It is estimated that global warming and extreme heat events will continue to exert negative effects on agricultural production in the future (Rosenzweig *et al*., 2014). Previous studies have reported the enhancement of heat stress tolerance in rice by genetic engineering, such as the overexpression of the *Arabidopsis* molecular chaperone *HSP101* or the rice transcription factor *OsWRKY11* in transgenic rice. Overexpression of these genes decreased the necrosis of leaves after heat stress treatment in rice plants (Katiyar‐Agarwal *et al*., 2003; Wu *et al*., 2009b); however, most of these studies did not reported differences in the transcriptomes of transgenic plants by gene transfer under nonstress conditions. Changes in the transcriptome in response to the overexpression of a gene under nonstress conditions might cause unexpected effects that are disadvantageous for agricultural applications.

We previously reported that DPB3‐1 interacts with the transcription factor DREB2A and that the overexpression of *DPB3‐1* improves heat stress tolerance in *Arabidopsis* by increasing the expression of many stress‐inducible genes under heat stress conditions. DREB2A is an important transcription factor that regulates both heat and drought stress responses in *Arabidopsis*, and DREB2A activates gene expression by binding to dehydration‐responsive element (DRE) on the target promoters (Sakuma *et al*., 2006a,b). A phenotypic analysis suggested that DPB3‐1 would be a useful factor for generating heat stress‐tolerant crops because the overexpression of *DPB3‐1* did not have negative effects on vegetative growth in *Arabidopsis* (Sato *et al*., 2014). In this study, interactions between DPB3‐1 and DREB2A homologues in rice and soya bean were confirmed. The effects of *DPB3‐1* overexpression on vegetative and reproductive growth were assessed under nonstress conditions in rice; heat stress tolerance in *DPB3‐1* overexpressing rice was also assessed.

## Results

### *Arabidopsis* DPB3‐1 interacts with DREB2A homologues in rice and soya bean

**Figure 1:**
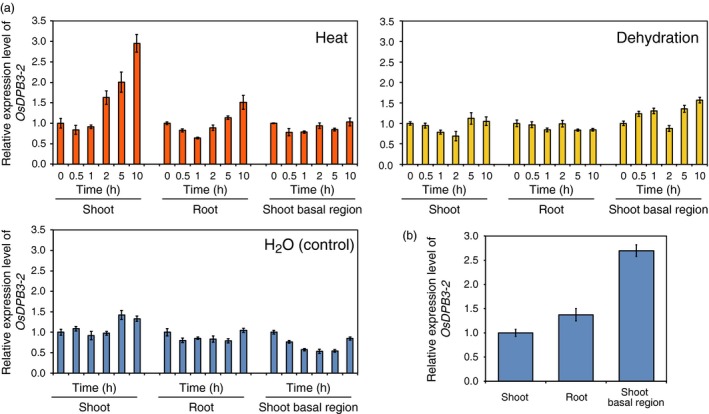
Expression profiles of the rice gene *OsDPB3‐2*. (a) *OsDPB3‐2* expression levels in different rice tissues under heat (42 °C), dehydration or nonstress (H_2_O) conditions. *OsDPB3‐2* expression levels were calculated using quantitative RT‐PCR analysis. The expression levels at 0 h under each condition were defined as 1.0. The error bars indicate the SD (*n* = 3). (b) *OsDPB3‐2* expression levels in different tissues under nonstress conditions. The expression levels in shoot tissues were defined as 1.0. The error bars indicate the SD (*n* = 3).

**Figure 2:**
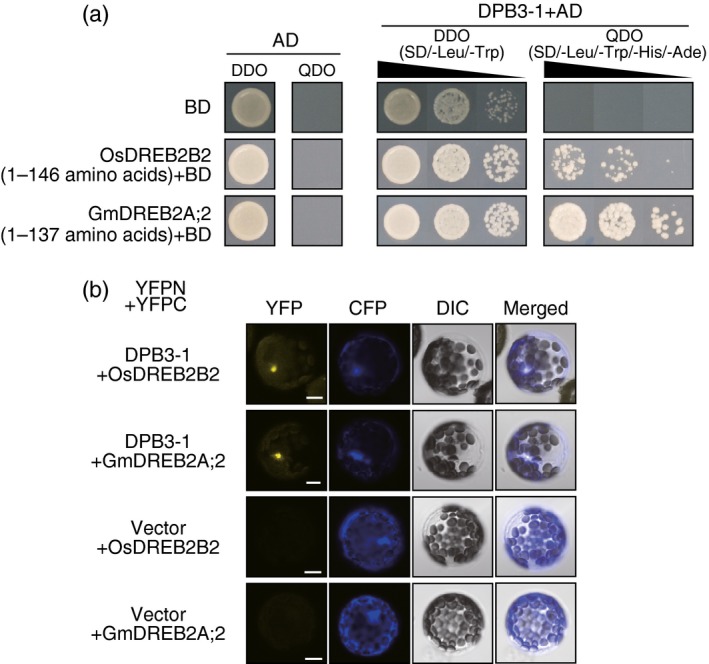
Interactions of DPB3‐1 with OsDREB2B2 and GmDREB2A;2 in yeast and *Arabidopsis* mesophyll cells. (a) The growth of yeast cells harbouring DPB3‐1 fused to the GAL4 activation domain (AD). SD/‐Leu/‐Trp (DDO) was the nonselective medium, and SD/‐Leu/‐Trp/‐His/‐Ade (QDO) was the selective medium. The N‐terminal region of OsDREB2B or GmDREB2A;2 was expressed as a fusion protein with the GAL4 binding domain (BD). (b) Verification of the interaction between DPB3‐1 and DREB2A homologous proteins by the BiFC system in *Arabidopsis* mesophyll protoplasts. Two constructs expressing a fusion protein of DPB3‐1 and the N‐terminal half of YFP (DPB3‐1‐YFPN) and a fusion protein of a DREB2A homologue protein and the C‐terminal half of YFP (OsDREB2B‐YFPC or GmDREB2A;2‐YFPC) were transfected. A construct expressing CFP was co‐transfected to identify transfected protoplasts. The transfected protoplasts were treated with 25 μm MG132, a 26S proteasome inhibitor, for 2 h in the dark. Differential interference contrast (DIC) images, confocal images of YFP and CFP fluorescence, and merged images are shown. Scale bars represent 10 μm.

Recently, we reported that DPB3‐1 interacts with the N‐terminal region of DREB2A and functions as a positive regulator of DREB2A specifically under heat stress conditions (Sato *et al*., 2014). DPB3 family proteins are widely conserved among land plants, including rice and soya bean (Sato *et al*., 2014), indicating that DPB3‐1 might positively regulate DREB2A homologues in various crop plants. Initially, we analysed the reproductive growth phenotypes and yield of *DPB3‐1*‐overexpressing *Arabidopsis* to confirm the effects of *DPB3‐1* overexpression on yield (Figure S1). The results indicated that the overexpression of *DPB3‐1* did not have negative effects on reproductive growth and seed formation (Table S1). We also examined the expression levels of a rice *DPB3‐1* homologue, *OsDPB3‐2* (*LOC_Os03g63530*), under abiotic stress conditions in seedlings. Expression of the *OsDPB3‐2* gene was induced by heat stress in shoots (Figure 1a). The gene was highly expressed in shoot basal regions, including meristematic cells (Figure 2); this expression pattern has also been observed for *DPB3‐1* in *Arabidopsis* (Sato *et al*., 2014). These findings suggest that OsDPB3‐2 has conserved functions similar to DPB3‐1 in rice. The expression patterns of *OsDPB3‐2* were different from those of *DPB3‐1*;* OsDPB3‐2* was expressed in both roots and shoots under nonstress conditions and was not repressed by dehydration stress (Figure 1a). However, *DPB3‐1* was primarily expressed in aerial regions and was repressed by dehydration (Figure 1b). These results imply that OsDPB3‐2 has specific functions in these tissues under such conditions.

Next, we investigated the interaction between DPB3‐1 and DREB2A homologues. We selected OsDREB2B2 and GmDREB2A;2 because these proteins were previously identified as canonical orthologues of DREB2A in rice and soya bean, respectively (Matsukura *et al*., 2010; Mizoi *et al*., 2013). Yeast two‐hybrid assays revealed that the N‐terminal regions of OsDREB2B2 and GmDREB2A;2 were sufficient for the interaction with DPB3‐1 (Figure 2a). For the BiFC tests, we expressed one fusion protein consisting of each DREB2A homologue and the C‐terminal half of yellow fluorescent protein (YFP; OsDREB2B2‐YFPC or GmDREB2A;2‐YFPC) and one fusion protein consisting of DPB3‐1 and the N‐terminal half of YFP (DPB3‐1‐YFPN) in protoplasts prepared from *Arabidopsis* mesophyll cells. Fluorescent signals were detected in the nuclei after treatment with MG132, an inhibitor of the 26S proteasome that promotes the accumulation of DREB2A homologue proteins (Mizoi *et al*., 2013; Morimoto *et al*., 2013; Sato *et al*., 2014; Figure 2b). These results suggested that DPB3‐1 interacts directly with OsDREB2B2 and GmDREB2A;2 in the nuclei. The high homology between the N‐terminal regions of the DREB2A homologues might have resulted in the interaction with DPB3‐1 (Figure S2).

**Figure 3:**
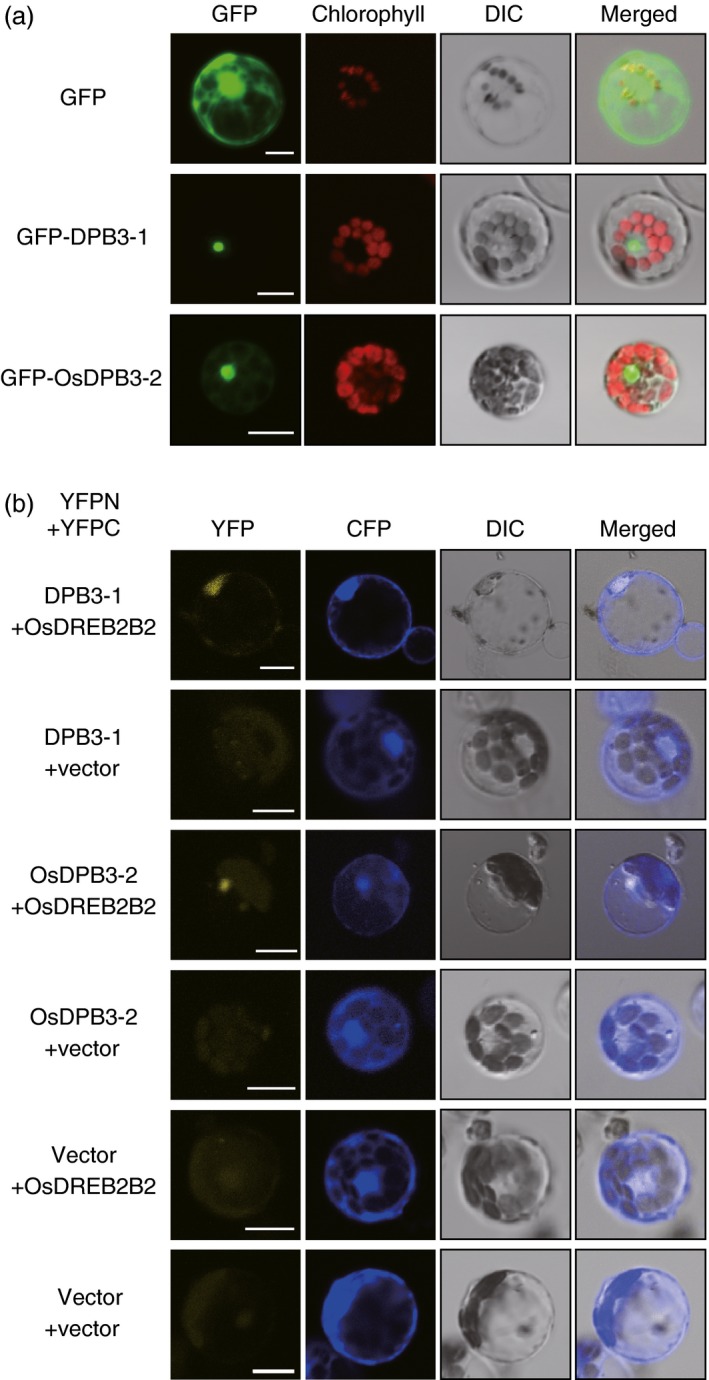
Subcellular localizations of DPB3‐1 and OsDPB3‐2 and the interaction between DPB3‐1 and OsDPB3‐2 with OsDREB2B2 in rice cells. (a) The subcellular localizations of DPB3‐1 and OsDPB3‐2 proteins fused to sGFP (GFP‐DPB3‐1 and GFP‐OsDPB3‐2) in rice mesophyll protoplasts. Protoplasts were transfected with the construct or the empty vector as a control. DIC images, confocal images of GFP fluorescence, confocal images of chlorophyll fluorescence and merged images are shown. Scale bars represent 10 μm. (b) Verification of the interaction between DPB3‐1 and OsDPB3‐2 with OsDREB2B2 using the BiFC system in rice mesophyll protoplasts. Rice mesophyll protoplasts were transfected with two constructs expressing OsDREB2B2‐YFPC and either DPB3‐1‐YFPN or OsDPB3‐2‐YFPN. The analysis was performed as described in Figure 2b. DIC images, confocal images of YFP and CFP fluorescence, and merged images are shown. Scale bars represent 10 μm.

We further performed a protein interaction analysis between DPB3‐1 and OsDREB2B2 using rice mesophyll protoplasts. A fusion protein of green fluorescent protein (GFP) and DPB3‐1 localized to the nuclei of the rice protoplasts (Figure 3a). Coexpression of OsDREB2B2‐YFPC and DPB3‐1‐YFPN in rice protoplasts treated with MG132 also produced YFP fluorescent signals in the nuclei (Figure 3b). This result indicated that DPB3‐1 interacts with OsDREB2B2 in the nuclei of rice cells. We also found that OsDPB3‐2 was localized to the nuclei (Figure 3a) and interacted with OsDREB2B2 (Figure 3b) in rice protoplasts.

### A trimer composed of *Arabidopsis* NF‐YA2, NF‐YB3 and DPB3‐1 enhances reporter activity together with DREB2A homologues

**Figure 4:**
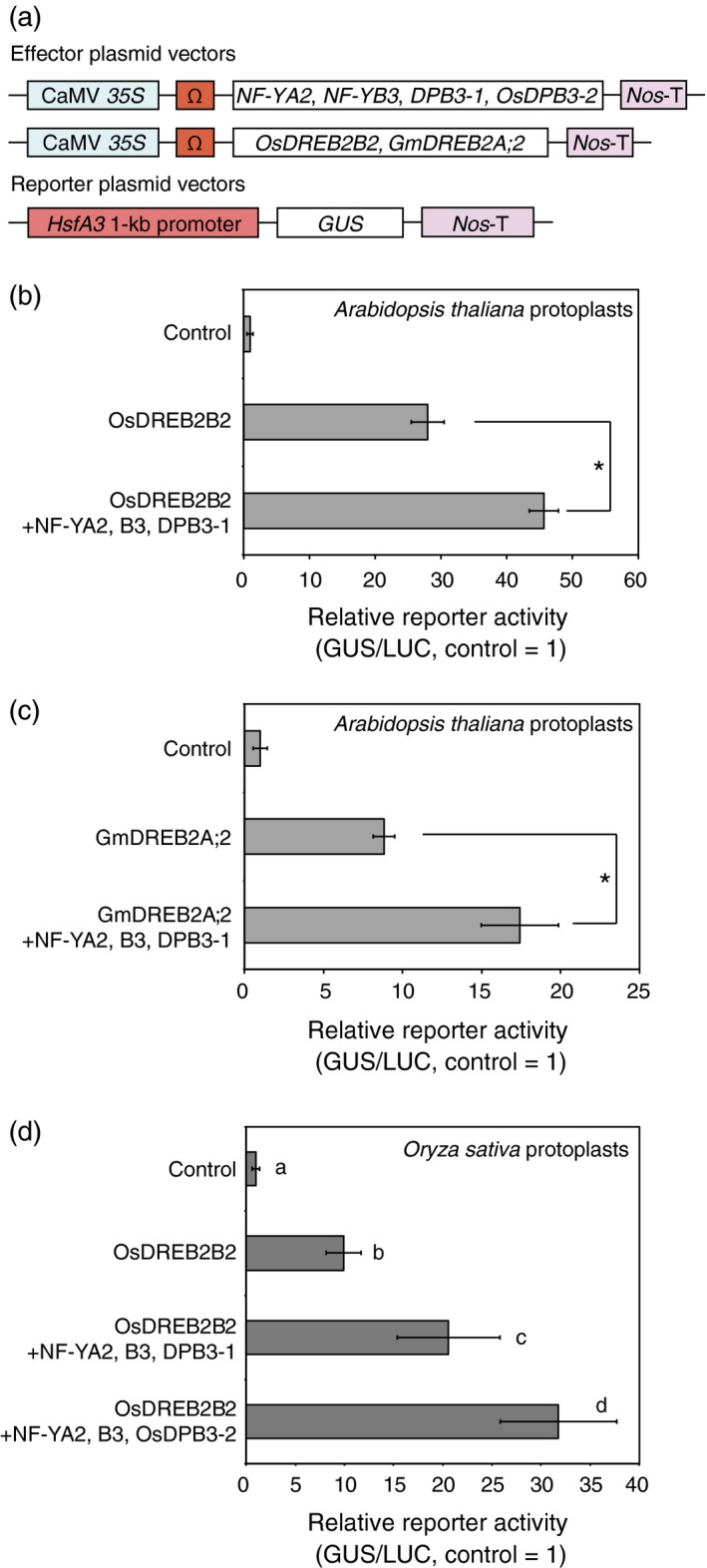
Transactivation of the *HsfA3* promoter by DREB2A homologues with NF‐YA2, NF‐YB3, DPB3‐1 and OsDPB3‐2. (a) Schematic diagram of the effector and reporter constructs for the transactivation analysis. The plasmids containing the *CaMV 35S* promoter and the tobacco mosaic virus Ω sequence fused to the *NF‐YA2*,*NF‐YB3*,*DPB3‐1*,*OsDPB3‐2*,*OsDREB2B2* and *GmDREB2A;2* coding sequence were cotransfected into protoplasts with the reporter plasmids harbouring the *HsfA3* 1‐kb promoter:*GUS* fusion gene. *Nos*‐T indicates the terminator sequence of the gene for nopaline synthetase. (b–d) Transactivation analysis of the *HsfA3* 1‐kb promoter:*GUS* reporter gene by DREB2A homologous proteins in cooperation with NF‐YA2, NF‐YB3, DPB3‐1 and OsDPB3‐2. Bars indicate mean and SD values from assays performed in triplicate, and asterisks and letters indicate significant differences between the reporter activities (*P* < 0.05 according to Student's *t*‐test or Tukey's multiple range test). The *35S:LUC* plasmid was also cotransfected in each experiment as an internal control. Transactivation analysis using OsDREB2B2 (b) or GmDREB2A;2 (c) with the trimer of NF‐YA2, NF‐YB3 and DPB3‐1 in *Arabidopsis* mesophyll protoplasts. (d) Transactivation analysis with OsDREB2B2 and the trimer of NF‐YA2, NF‐YB3 and DPB3‐1, or the trimer of NF‐YA2, NF‐YB3 and OsDPB3‐2 in rice mesophyll protoplasts.

To analyse the effect of DPB3‐1 on the transcriptional activity of the DREB2A homologues, we performed transactivation assays using *Arabidopsis* protoplasts. A trimer composed of *Arabidopsis* NF‐YA2, NF‐YB3 and DPB3‐1 has been reported to act as a positive regulator of DREB2A (Sato *et al*., 2014). The three subunits were coexpressed with OsDREB2B2 or GmDREB2A;2 in *Arabidopsis* mesophyll protoplasts using an *Arabidopsis HsfA3* promoter‐*GUS* reporter (Figure 4a). The results showed that the coexpression of DPB3‐1, NF‐YA2 and NF‐YB3 with the DREB2A homologues significantly increased the transactivation of the reporter gene (Figure 4b,c). These data suggest that DPB3‐1 positively regulates the transcriptional activity of OsDREB2B2 and GmDREB2A;2 via the formation of trimers. A similar transactivation assay was also performed in rice mesophyll protoplasts using the same effector and reporter genes. Again, the coexpression of DPB3‐1, NF‐YA2 and NF‐YB3 significantly enhanced transactivation of the reporter gene together with OsDREB2B2 (Figure 4d). This result implies that DPB3‐1 functions as a positive regulator of OsDREB2B2 in rice cells in cooperation with NF‐YA2 and NF‐YB3 homologues. Moreover, the trimer formed by NF‐YA2, NF‐YB3 and rice OsDPB3‐2 also enhanced the reporter activity with OsDREB2B2 (Figure 4d). This result confirmed that the functions of DPB3 family proteins were conserved among land plants. Interestingly, the trimer formed by NF‐YA2, NF‐YB3 and OsDPB3‐2 enhanced reporter activity with OsDREB2B2 significantly more than the trimer formed by NF‐YA2, NF‐YB3 and DPB3‐1 (Figure 4d).

### Overexpression of *DPB3‐1* does not have negative effects on normal vegetative, reproductive growth or yield in rice

**Figure 5:**
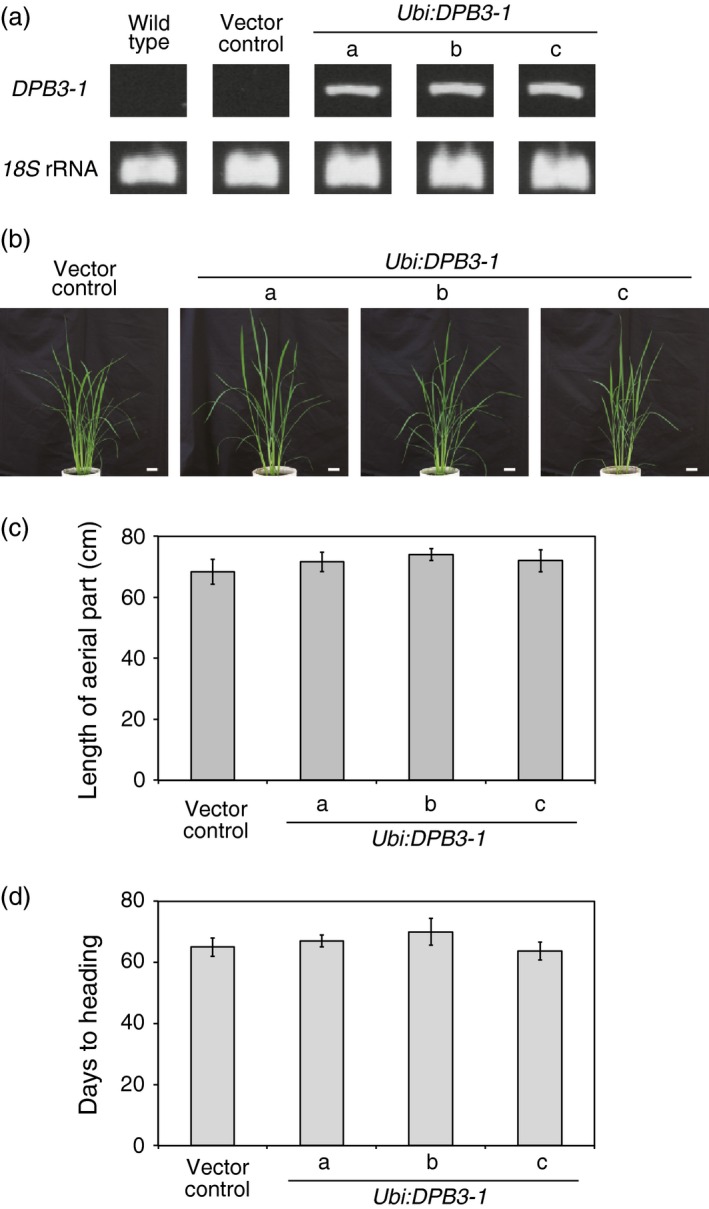
Phenotype of *DPB3‐1*‐overexpressing rice under nonstress conditions. (a) Confirmation of the expression of *DPB3‐1* in wild‐type, vector control and *Ubi:DPB3‐1* plants by RT‐PCR analysis. *18S* rRNA was amplified as an internal control. The primers used for PCR are shown in Table S11. (b) Growth of *DPB3‐1*‐overexpressing plants under normal conditions. Photographs of 50‐day‐old plants are shown. Scale bars represent 5 cm. (c) Average length of the aerial portion of the vector control and *DPB3‐1*‐overexpressing plants calculated from the plants grown as in (b). The error bars indicate the SD (*n* = 12). The data were evaluated using one‐way ANOVA, and no significant differences were detected (*P* > 0.05). (d) Average days to heading in the vector control and *DPB3‐1*‐overexpressing plant under normal conditions. Days to heading were scored from sowing to emergence of panicles from the main culms. The error bars indicate the SD (*n* = 12). The data were evaluated using one‐way ANOVA, and no significant differences were detected (*P* > 0.05).

To examine the effects of *DPB3‐1* on growth and heat stress tolerance in rice, *DPB3‐1*‐overexpressing rice plants were generated (*Ubi:DPB3‐1*‐a, b, c; Figure 5a). The vegetative growth of the *DPB3‐1*‐overexpressing rice was compared to that of vector control plants at several developmental stages under nonstress conditions (Figure 5b,c, also see Figure S3a–d). No visible phenotypic differences were observed between the transgenic and control plants, and there were no differences in the heading date (Figure 5d). We measured the yield parameters of the *DPB3‐1*‐overexpressing rice under nonstress conditions. The tiller, panicle and effective grain numbers per plant of the *DPB3‐1*‐overexpressing rice were not significantly different from those of the vector control rice (Table 1, also see Figure S4a). The seed morphology was also not significantly changed in response to *DPB3‐1* overexpression (Table 1, also see Figure S4a,b). These data suggest that the overexpression of *DPB3‐1* in rice does not have negative effects on plant vegetative growth and reproductive development under nonstress conditions.

**Table 1:** Yield parameters of vector control and *DPB3‐1*‐overexpressing rice under nonstress conditions

Traits	Vector control (%)	*Ubi:DPB3‐1*‐a (%)	*Ubi:DPB3‐1*‐b (%)
Tiller number per plant	31 ± 5 (100)	36 ± 3 (117)	28 ± 2 (92)
Panicle number per plant	27 ± 5 (100)	30 ± 4 (111)	26 ± 2 (94)
Effective grain number per plant	516 ± 136 (100)	518 ± 146 (100)	594 ± 127 (115)
Grain length (mm)	4.9 ± 0.2 (100)	5.0 ± 0.2 (102)	4.7 ± 0.2 (96)
Grain width (mm)	2.8 ± 0.1 (100)	2.7 ± 0.1 (95)	2.6 ± 0.1 (93)
Grain thickness (mm)	2.0 ± 0.2 (100)	2.1 ± 0.2 (104)	2.1 ± 0.2 (104)
1000‐grain weight (g)	45.7 ± 2.3 (100)	45.0 ± 2.1 (99)	44.7 ± 3.7 (98)

Various parameters involved in the yield and seed morphology were measured. Values represent the means and SD (*n* = 12). Relative percentages are shown in brackets. The data were evaluated using one‐way ANOVA, and no significant differences were detected (*P* > 0.05).

### *DPB3‐1*‐overexpressing rice exhibit enhanced heat stress tolerance

**Figure 6:**
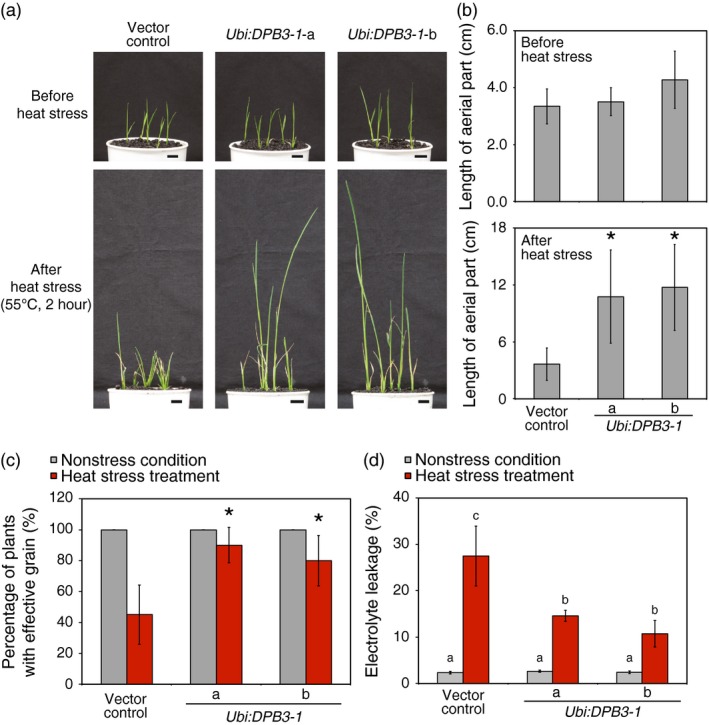
Heat stress tolerance of *DPB3‐1*‐overexpressing plants. (a) Photographs of plants before and after heat stress treatment. The seeds were germinated in water for 7 days at 28 °C, and after germination, the seedlings were grown on soil for 2 days. After heat stress treatment (55 °C, 2 h), the plants were grown under normal conditions. Photographs were taken before stress treatment and after a 10‐day recovery period at 28 °C. Scale bars represent 1 cm. (b) Average length of the aerial portion of the vector control and *DPB3‐1*‐overexpressing plants before and after heat stress treatment calculated from the plants grown as shown in (a). The error bars indicate the SD (*n* = 15). Asterisks indicate significant differences between the plants (*P* < 0.05 according to the Bonferroni‐corrected Student's *t*‐test). (c) Percentage of plants that grew to the reproductive stage under normal conditions or after heat stress treatment. The stress‐treated plants were grown continuously under normal conditions, and the percentages of plants that produced grains were scored. All of the plants without heat stress treatment produced grains. The error bars indicate the SD (*n* = 15). Asterisks indicate significant differences between the plants after heat stress treatment (*P* < 0.05 according to the Bonferroni‐corrected Student's *t*‐test). (d) Electrolyte leakage of plants with or without heat stress treatment (42 °C, 24 h). The error bars indicate the SD (*n* = 12). There were no significant differences between plants without heat stress (*P* > 0.05 according to one‐way ANOVA), and letters indicate significant differences between the plants after heat stress treatment (*P* < 0.05 according to Tukey's multiple range test).

We evaluated whether *DPB3‐1*‐overexpressing rice possessed enhanced heat stress tolerance. The vector control and *DPB3‐1*‐overexpressing plants were subjected to heat stress at 55 °C for 2 h. The length of the aerial portion of each plant was measured after 10 days under nonstress conditions. The growth of the vector control plants was clearly suppressed by heat stress even after 10 days of recovery, while the *DPB3‐1*‐overexpressing plants displayed significantly better growth (Figure 6a,b). Almost all of the *DPB3‐1*‐overexpressing plants grew to reproductive stage after heat stress treatment, while approximately half of the vector control plants did not generate reproductive organs (Figure 6c). These results suggest that the overexpression of *DPB3‐1* in rice enhances heat stress tolerance and contributes to improved growth after heat stress treatment. Moreover, electrolyte leakage in the vector control and *DPB3‐1*‐overexpressing rice was measured to evaluate the extent of cell membrane damage after heat stress treatment. The results of the electrolyte leakage assays were similar in the vector control and *DPB3‐1*‐overexpressing rice under nonstress conditions; however, the *DPB3‐1*‐overexpressing rice maintained significantly lower levels of electrolyte leakage after heat stress treatment (42 °C, 24 h) compared with the vector control rice (Figure 6d), suggesting that the overexpression of *DPB3‐1* increases heat stress tolerance at the cellular level.

### The expression levels of several heat stress‐inducible genes are increased in *DBP3‐1*‐overexpressing rice under heat stress conditions

**Figure 7:**
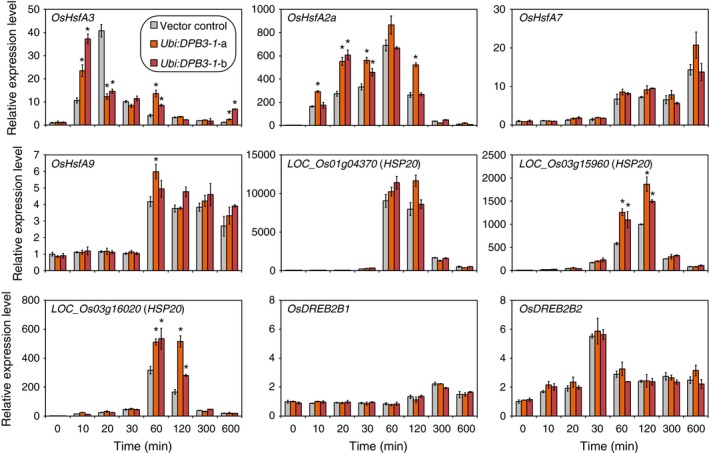
Gene expression patterns of the vector control and *Ubi:DPB3‐1* plants under heat stress conditions. The expression levels of four *HsfA* family genes (OsHsfA3,OsHsfA2a,OsHsfA7 and OsHsfA9), three *HSP20* family genes (*LOC_Os01g04370*,*LOC_Os03g15960* and *LOC_Os03g16020*) and two splicing variants of *OsDREB2B* in the vector control and *DPB3‐1*‐overexpressing plants (*Ubi:DPB3‐1*‐a and b) in response to heat stress conditions (42 °C) were calculated by quantitative RT‐PCR analysis. The expression levels of each gene in the vector control plants were defined as 1.0. The error bars indicate the SD (*n* = 3). Asterisks indicate significant differences between the plants at each time point (*P* < 0.05 according to the Bonferroni‐corrected Student's *t*‐test).

We analysed the expression levels of several heat stress‐inducible genes in *DBP3‐1*‐overexpressing plants under heat stress conditions (42 °C). A previous study revealed that the overexpression of *DPB3‐1* in *Arabidopsis* increases the expression levels of *Arabidopsis HsfA2*,* HsfA3* and *At1g75860*, the latter of which encodes an HSP20 family protein, under heat stress conditions (Sato *et al*., 2014). Therefore, the expression levels of several genes that encode proteins homologous to rice HsfA3, HsfA2 (Guo *et al*., 2008; Wang *et al*., 2009) and At1g75860 (Figure S5) were analysed. *LOC_Os02g32590* (*OsHsfA3*), *LOC_Os03g53340* (*OsHsfA2a*), *LOC_Os01g39020* (*OsHsfA7*) and *LOC_Os03g12370* (*OsHsfA9*) were examined as *HsfA* family genes, and *LOC_Os01g04370*,* LOC_Os03g15960* and *LOC_Os03g16020* were examined as *HSP20* family genes based on their phylogenetic profiles. The expression levels of *OsDREB2B1* and *OsDREB2B2* were also analysed as controls. The results revealed that the expression levels of *OsHsfA3*,* OsHsfA2a*,* LOC_Os03g15960* and *LOC_Os03g16020* were significantly enhanced in two lines of *DPB3‐1*‐overexpressing plants (*Ubi:DPB3‐1*‐a and b) at several time points in comparison with those of the vector control, while the expression levels of *OsHsfA7*,* OsHsfA9*,* LOC_Os01g04370*,* OsDREB2B1* and *OsDREB2B2* were similar between *DPB3‐1*‐overexpressing and vector control plants (Figure 7). It was suggested that DPB3‐1 positively regulates the expression of those up‐regulated genes under heat stress conditions in rice. The number of abiotic stress‐related *cis* elements, DREs, CCAAT motifs (Sato *et al*., 2014), heat shock elements (HSEs; Yoshida *et al*., 2011) and ABA‐responsive elements (ABREs; Yoshida *et al*., 2015) on the 1‐kb promoters of each gene is shown in Table S2. The expression levels of several heat stress‐inducible genes might be enhanced by the overexpression of DPB3‐1 through these *cis* elements.

### Microarray analysis reveals that the overexpression of *DPB3‐1* enhances the expression levels of heat stress‐responsive genes in rice

**Figure 8:**
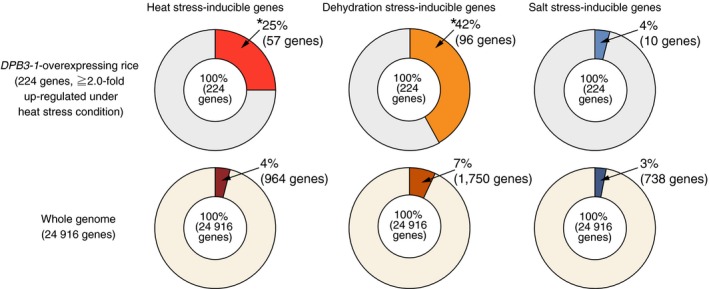
Proportions of abiotic stress‐inducible genes among the up‐regulated genes in *DPB3‐1*‐overexpressing rice under heat stress conditions or in the whole rice genome. The proportions of abiotic stress‐inducible genes were calculated according to the microarray analysis (Table S3) and previous papers (Maruyama *et al*., 2012; Venu *et al*., 2013). Asterisks indicate significant differences between the proportions of abiotic stress‐inducible genes among the up‐regulated genes in *DPB3‐1*‐overexpressing rice under heat stress conditions or in the whole rice genome (*P* < 0.0001, Fisher's exact test).

**Figure 9:**
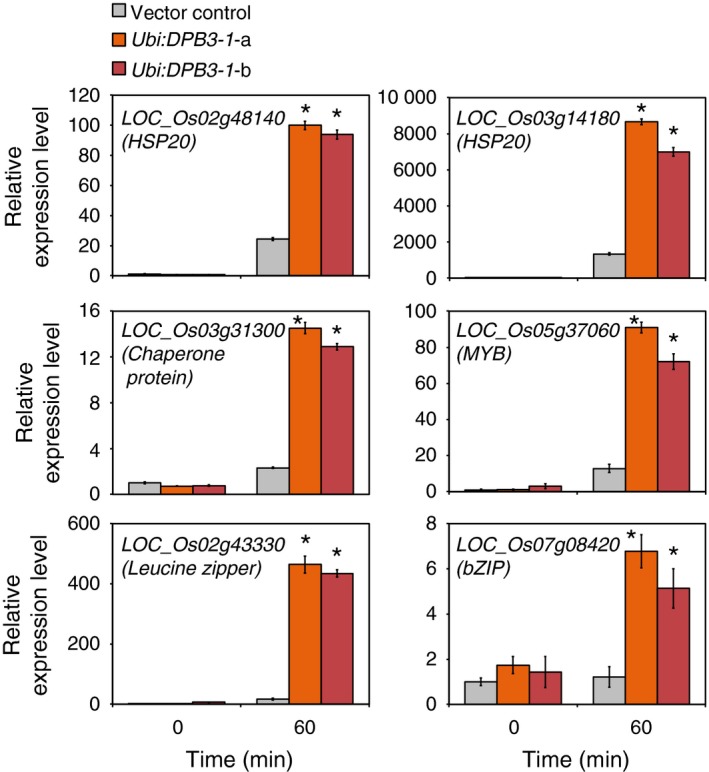
Confirmation of up‐regulated gene expression in *Ubi:DPB3‐1* by microarray analysis. The expression levels of six up‐regulated genes in response to the overexpression of *DPB3‐1* under heat stress conditions were analysed by quantitative RT‐PCR analysis. Five genes other than *LOC_Os07g08420* (*bZIP* family gene) could be induced by heat stress in the vector control rice. The expression levels of each gene in the vector control plants were defined as 1.0. The error bars indicate the SD (*n* = 3). Asterisks indicate significant differences between the plants at each time point (*P* < 0.05 according to the Bonferroni‐corrected Student's *t*‐test).

To reveal the effects of *DPB3‐1* overexpression on the rice transcriptome under control and heat stress conditions, we performed microarray analyses at 0‐ and 60‐min time points under heat stress conditions (42 °C) using vector control and *DPB3‐1*‐overexpressing rice (*Ubi:DPB3‐1*‐a). In the vector control rice, 962 and 918 genes were induced (Table S3) and repressed (Table S4), respectively, more than twofold in response to heat stress [VC (heat) vs. VC (control)]. Compared with the vector control rice, one gene was down‐regulated (Table S5) and no genes were up‐regulated more than twofold in the *Ubi:DPB3‐1* plants under control conditions [*Ubi:DPB3‐1* (control) vs. VC (control)]. Under heat stress conditions, however, 224 genes were up‐regulated (Table S6) and 161 genes down‐regulated (Table S7) more than twofold in *Ubi:DPB3‐1* plants [*Ubi:DPB3‐1* (heat) vs. VC (control)]. Among the 224 up‐regulated genes in *Ubi:DPB3‐1* rice, 57 genes were heat stress‐inducible genes (Table S5). The proportion of heat stress‐inducible genes among the 224 up‐regulated genes in *Ubi:DPB3‐1* rice (25%) was significantly higher than that in the entire rice genome (4%; *P* < 0.0001; Figure 8), while 8 among the 161 down‐regulated genes in *Ubi:DPB3‐1* rice were heat stress‐suppressed genes (Table S6). There were no significant differences between the proportions of heat stress‐suppressed genes among the 161 down‐regulated genes (5%) and the entire rice genome (4%; *P* > 0.5; Figure S6). The expression levels of several heat stress‐inducible (Figure 9) or stress‐suppressed (Figure S7) genes identified by microarray were confirmed by quantitative RT‐PCR.

We also performed gene ontology (GO) enrichment analysis using the GO term enrichment tool available at the Gene Ontology Consortium (http://geneontology.org/; Blake *et al*., 2013; Gene Ontology Consortium, 2015). When the 224 up‐regulated genes were compared to the *Oryza sativa* genome background, we found that the term ‘response to heat’ was significantly enriched (*P* < 0.001; Table S8). This result coincides with a significant increase in heat stress‐inducible genes in the up‐regulated genes in *Ubi:DPB3‐1* rice. The GO analysis using the 161 down‐regulated genes in *Ubi:DPB3‐1* under heat stress conditions revealed the significant enrichment of terms related to several metabolic processes (*P* < 0.001; Table S9).

To analyse the expression patterns of genes affected by the overexpression of *DPB3‐1* under heat stress conditions, a metaprofile analysis was performed using a public microarray database (Genevestigator; https://genevestigator.com/gv/) based on the top 100 up‐regulated or down‐regulated genes in *Ubi:DPB3‐1* plants in response to heat stress conditions. The results indicated that many of the genes that were up‐regulated in *Ubi:DPB3‐1* were induced by drought as well as heat stress treatment (Figure S8a). The results also revealed that many of the genes that were up‐regulated in *Ubi:DPB3‐1* plants under heat stress conditions were down‐regulated in germination (Figure S8a), while the genes that were down‐regulated in *Ubi:DPB3‐1* plants showed the opposite trend (Figure S8b). In comparison with a previous transcriptome analysis of rice treated with dehydration stress, we found that 96 genes among the 224 up‐regulated genes were induced in response to dehydration stress (Maruyama *et al*., 2012). The proportions of these drought stress‐inducible genes among the 224 up‐regulated genes (42%) were significantly higher than their proportions in the entire *Oryza sativa* genome (7%; *P* < 0.0001), while the proportion of salt stress‐inducible genes among the 224 up‐regulated genes (4%) did not differ significantly from that of the entire *Oryza sativa* genome (3%; *P* > 0.1; Venu *et al*., 2013; Figure 8).

To reveal the mechanisms by which DPB3‐1 controls the expression levels of its target genes under heat stress conditions, we compared the frequency of each hexamer sequence in the promoter of the top 100 up‐regulated or down‐regulated genes in *DBP3‐1*‐overexpressing rice in response to heat stress conditions with their normalized frequencies in the promoters of the entire *Oryza sativa* genome. The Z‐scores of all hexamer sequences are presented in scatter plots. In the promoters of the up‐regulated genes in *DPB3‐1*‐overexpressing plants, the ABRE sequence (ACGTGT) was the most conserved sequence, and other ABRE (CCACGT) and ABRE‐like sequences (GCCACG and CGTGTC) were highly conserved (Figure S8c). The G box sequence (CACGTG), coupling element 3 (CE3; CACGCG) and CE3‐like sequences (ACGCGT) were also highly conserved in the up‐regulated genes (Figure S8c). However, these *cis* elements were not conserved in the down‐regulated genes in *Ubi:DPB3‐1* plants (Figure S8d). The ABRE was identified on the promoter of ABA‐responsive genes, and bZIP transcription factors are thought to bind to these sequences (Fujita *et al*., 2005; Yang *et al*., 2011; Yoshida *et al*., 2015). Moreover, the bZIP transcription factors also bound to the G box (De Jong, 2013; Jakoby *et al*., 2002), and CE3 has been reported to strongly co‐localize with the ABRE motif on rice promoters of ABA‐ or drought‐responsive genes (Gómez‐Porras *et al*., 2007; Maruyama *et al*., 2012). These results suggest that DPB3‐1 positively regulates the transcriptional activation of bZIP transcription factors as a coactivator or that DPB3‐1 increases the expression levels of genes encoding a bZIP transcription factor as a downstream target (Table S6; Figure 9). Considering the enhanced expression levels of drought stress‐inducible genes in *Ubi:DPB3‐1* rice under heat stress conditions and the significant enrichment of ABRE‐related *cis* elements on the promoters of up‐regulated genes, we evaluated the drought stress tolerance of *Ubi:DPB3‐1* rice. However, there were no differences between the vector control and *Ubi:DPB3‐1* plants in terms of plant growth, leaf wilting and electrolyte leakage after drought stress treatment (Figure S9a,b).

We also assessed the frequency of the DRE, CCAAT and HSE motifs in the promoters of the top 100 up‐regulated genes in *DPB3‐1*‐overexpressing plants under heat stress conditions. One of the DRE motifs (ACCGAC) was relatively highly conserved (*Z*‐score > 3, *P* < 0.001) among the DRE sequences (Table S10). However, the CCAAT and HSE motifs were not significantly enriched in the promoters of the up‐regulated genes in *Ubi:DPB3‐1* rice. It is possible that DPB3‐1 interacts with OsDREB2B2 and positively regulates its transcriptional activity. They may then enhance the expression levels of target genes through the DRE (ACCGAC) sequences in the promoters of rice under heat stress conditions.

## Discussion

Losses of crop production as a consequence of extreme heat stress events have been reported in various regions of the world (Ciais *et al*., 2005; Smith and Katz, 2013; Tian *et al*., 2009). The generation of heat‐tolerant crops by genetic engineering is a key challenge to improving global food security. The utility of DPB3‐1 was suggested in a previous study because the overexpression of *DPB3‐1* improved heat stress tolerance in *Arabidopsis* without growth retardation. In the present study, we generated *DPB3‐1*‐overexpressing transgenic rice and evaluated tolerance to heat stress. *DPB3‐1*‐overexpressing rice displayed enhanced heat stress tolerance compared to vector control rice (Figure 6a,b). Moreover, a greater proportion of *DPB3‐1*‐overexpressing rice plants grew to the reproductive stage after the heat stress treatment (Figure 6c). Because the overexpression of *DPB3‐1* showed no negative effects on vegetative and reproductive growth or yield in rice under control conditions (Figure 5; Table 1, and also see Figures S3 and S4), the *DPB3‐1* gene was considered to be a useful target for generating crops with enhanced heat stress tolerance.

One of the advantages associated with increasing heat stress tolerance via DPB3‐1 is the high conservation of DPB3 family proteins among land plants. A previous study revealed that DPB3‐1 belongs to a monophyletic subgroup that is conserved among land plants, including rice and soya bean (Sato *et al*., 2014). The rice gene *OsDPB3‐2* also had heat stress‐inducible expression similar to *DPB3‐1* in *Arabidopsis* (Figure 1). This result suggests that DPB3‐1 and its homologues share similar functions in land plants. Interaction and transactivation analyses using DREB2 family proteins in rice and soya bean further support this hypothesis (Figures 1, 2, 3). These findings raise the possibility of increasing heat stress tolerance in various crop plants, including soya bean, by overexpressing *DPB3‐1*. Moreover, further analyses of DPB3‐1 homologues in rice, soya bean (GmDPB3‐3; Glyma12g07390, GmDPB3‐4; Glyma11g20740) and other plants will reveal conserved roles and species‐specific functions of DPB3 family proteins. *OsDPB3‐2* showed specific expression profiles (Figure 1), and the trimer including OsDPB3‐2 enhanced the transcriptional activity of OsDREB2B2 more than the trimer including DPB3‐1 (Figure 4d). A comparison of heat stress tolerances between *Ubi:DPB3‐1* and *Ubi:OsDPB3‐2* rice should provide interesting insight into the enhancement of heat stress tolerance in monocots.

Our approach is unique in that DPB3‐1 is not a transcriptional activator; instead, it acts as a coactivator. The DPB3‐1 protein itself does not possess transcriptional activity. Therefore, the overexpression of *DPB3‐1* had almost no effect on gene expression patterns under nonstress conditions (Table S5). The DPB3‐1 protein affected gene expression only in the presence of its interacting transcription activators, which were induced under heat stress conditions (Tables S6 and S7). This characteristic of DPB3‐1 should be advantageous in agricultural applications. The overexpression of functional transcriptional activators often causes unexpected and negative effects on plant growth and agricultural productivity under normal growth conditions (Ito *et al*., 2006; Tang *et al*., 2012; Wu *et al*., 2009a). DPB3‐1 and its homologous proteins should be useful in enhancing heat stress tolerance in crops without growth retardation.

A microarray analysis of *DPB3‐1*‐overexpressing rice under heat stress conditions clearly indicated that the overexpression of *DPB3‐1* contributed to heat stress tolerance through enhanced gene expression of many heat stress‐inducible genes (Figure 8; Table S6). Moreover, GO analysis also confirmed that the heat stress‐related genes were significantly enriched among the up‐regulated genes (Table S8). However, the GO analysis suggested that genes related to primary metabolic processes were enriched among the down‐regulated genes in the *DPB3‐1*‐overexpressing plants (Table S9), and the expression profile analysis revealed that many of the down‐regulated genes in *DPB3‐1*‐overexpressing rice under heat stress conditions were induced during germination (Figure S8b). Numerous previous works have suggested that there is a trade‐off relationship between the stress response and growth in plants (Koehler *et al*., 2012; Lozano‐Durán *et al*., 2013; Zhang *et al*., 2014). It is possible that the suppression of metabolic‐ or growth‐related genes under heat stress conditions might contribute to the enhanced heat stress tolerance, but further analysis is required to reveal the effect of the down‐regulated genes during heat stress in *DPB3‐1*‐overexpressing rice.

In a previous work, DPB3‐1 was identified as a coactivator of DREB2A under heat stress conditions (Sato *et al*., 2014). Similarly, DPB3‐1 interacted with OsDREB2B2 (Figures 2 and 3) and significantly increased reporter activity in rice protoplasts together with OsDREB2B2 (Figure 4). Moreover, our over‐representation analysis of all hexamers revealed that the DRE sequence (ACCGAC) was highly enriched on the promoters of the up‐regulated genes in *DPB3‐1*‐overexpressing rice under heat stress conditions (Table S10). Quantitative RT‐PCR also indicated that the expression levels of genes homologous to DREB2A target genes in *Arabidopsis* were significantly enhanced in *DPB3‐1*‐overexpressing rice, and DRE sequences were found in their promoters (Figure 7; Table S2). DPB3‐1 was predicted to enhance the transcriptional activity of OsDREB2B2 and increase the expression levels of its target genes through DRE. The future identification of OsDREB2B2 target genes under heat stress conditions by microarray or ChIP‐seq analyses might aid in assessing the detailed role of OsDREB2B2 and DPB3‐1 during the heat stress response in rice.

Our microarray analysis also suggested that the overexpression of *DPB3‐1* enhanced heat stress tolerance through unknown transcription factors other than OsDREB2B2. The promoter analysis clearly indicated that ABRE and ABRE‐related *cis* elements were highly conserved in the up‐regulated genes in *DPB3‐1*‐overexpressing rice under heat stress conditions (Figure S8c). The ABRE motif is known to be recognized by several bZIP transcription factors in plants (Fujita *et al*., 2005; Yang *et al*., 2011; Yoshida *et al*., 2015). These results suggest that DPB3‐1 positively regulates bZIP transcription factors under heat stress conditions. Our microarray analysis of vector control rice (Table S3) and several previous studies (Jung *et al*., 2012; Zhang *et al*., 2013) indicate that *bZIP* family genes are induced under heat stress conditions. These heat stress‐responsive bZIP transcription factors might be candidates for the cofactor of DPB3‐1. The search for novel interacting bZIP transcription factors with DPB3‐1 may be necessary to reveal the mechanisms by which DPB3‐1 increases heat stress tolerance in rice.

Although our results clearly demonstrate that the overexpression of *DPB3‐1* enhances heat stress tolerance in rice without growth retardation, additional experiments are important to confirm its usability for agricultural applications. First, the heat stress response and tolerance of reproductive stages should be analysed in future studies. Many previous works have reported that plants are more sensitive to abiotic stresses, including high temperature, during the reproductive stage (Kim *et al*., 2001; Omae *et al*., 2012; Tunc‐Ozdemir *et al*., 2013; Zinn *et al*., 2010). The expression profiles of *HSF*,* HSP* and *DREB* family genes were conserved between the vegetative and reproductive stages in rice, and the ABRE motif has been suggested to be important for gene induction in response to heat stress in reproductive organs (Chauhan *et al*., 2011; Jagadish *et al*., 2010; Zhang *et al*., 2012). These data imply that the overexpression of *DPB3‐1* may also confer enhanced heat stress tolerance during reproductive stages. Furthermore, microarray analysis revealed that the expression levels of drought stress‐responsive genes were also enhanced in the *DPB3‐1*‐overexpressing rice under heat stress conditions (Figure 8 and Figure S8a); however, there were no significant differences between the vector control and *DPB3‐1*‐overexpressing rice in response to drought stress alone (Figure S9), which suggests that DPB3‐1 could contribute to the induction of drought stress‐responsive genes only under heat stress conditions. Previous studies have revealed that the combination of heat and drought stress had more severe effects on plant growth and development (Mittler, 2006; Rizhsky *et al*., 2004; Smith and Katz, 2013). Further studies are required to elucidate the mechanisms by which DPB3‐1 enhances the expression levels of drought stress‐inducible genes under heat stress conditions and whether DPB3‐1 might be a useful factor for increasing combined heat and drought stress tolerance in crops.

## Experimental procedures

### Plant materials, growth conditions and the generation of transgenic rice

Rice (*Oryza sativa* cv. Nipponbare) was grown as described previously (Ito *et al*., 2006) with minor modifications. After the seeds were exposed to 42 °C for 3 days, they were selected and germinated in water containing 50 mg/L hygromycin for 7–9 days at 28 °C day/25 °C night under 12‐h light/12‐h dark cycles at a photon flux density of 150 μmol/m^2^/s. After germination, the plants were transferred to soil in a plastic pot with a diameter of 6 cm and grown for 14 days under the conditions described above. Subsequently, the plants were transferred to a plant pot with a size of 1/50 m^2^ and grown at 28 °C day/25 °C night under natural light conditions. Transgenic rice was generated using *Agrobacterium*‐mediated transformation as described previously (Hiei *et al*., 1994). T1 and T2 seeds were used for subsequent experiments.

### Yeast two‐hybrid assays

Yeast two‐hybrid assays were performed as described in the user manual supplied with the Matchmaker Gold Two‐Hybrid System using strain AH109 (Clontech, Palo Alto, CA, USA).

### Transient expression in *Arabidopsis* and rice mesophyll protoplasts

Transient transformation of *Arabidopsis* and rice mesophyll protoplasts was performed as described previously (Chen *et al*., 2006; Yoo *et al*., 2007). Fluorescence observation using *Arabidopsis* or rice mesophyll protoplasts and transactivation assays using *Arabidopsis* protoplasts were performed as described previously (Sato *et al*., 2014). The transactivation assays using rice protoplasts were performed with minor modifications compared with the *Arabidopsis* protoplasts. The transformed rice protoplasts were disrupted in 50 μL of lysis buffer [50 mm Tris‐HCl (pH 7.5), 30% (v/v) glycerol and 10 mm 2‐mercaptoethanol] with a vortex mixer, and the solution was centrifuged at 20 000 g for 5 min. Luciferase activity was measured with a Picagene BrillianStar‐LT Luminescence Kit (Toyo B‐Net, Tokyo, Japan) according to the user manual. For the GUS assays, 30 μL of the protoplast lysate was added to 30 μL of reaction buffer [50 mm Tris‐HCl (pH 7.5), 30% (v/v) glycerol, 10 mm 2‐mercaptoethanol and 0.04% (w/v) MU glucuronide] and incubated at 37 °C for 2 h. To stop the reaction, 60 μL of 1 m Na_2_CO_3_ was added to the mixture, and LUC luminescence and MU fluorescence were measured using an ARVO MX plate reader (Perkin‐Elmer, Waltham, MA, USA).

### Heat and dehydration stress treatments

For the heat stress tolerance test, 9‐day‐old plants on soil were subjected to 55 °C for 2 h in an incubator (Yamato Scientific Co., Tokyo, Japan). For the electrolyte leakage assays, 16‐day‐old plants on soil were transferred to 42 °C for 24 h in an incubator. For the gene expression analysis, 9‐day‐old plants in sterilized water were transferred to an incubator at 42 °C for heat stress treatment; plants were transferred to petri dishes for dehydration stress treatment.

### RNA preparation and analysis of gene expression

Total RNA was isolated from 9‐day‐old plants using RNAiso plus (TaKaRa, Otsu, Shiga, Japan) according to the supplier's instructions. Syntheses of cDNA and quantitative RT‐PCR analysis were conducted as described previously (Sato *et al*., 2014).

### Microarray analysis

Microarray analysis was performed using a custom gene expression microarray for rice (Maruyama *et al*., 2014; Agilent Technologies, Palo Alto, CA, USA) as described previously (Mizoi *et al*., 2013) with minor modifications. Total RNA (200 ng) was labelled with a Low Input Quick Amp Labeling Kit (Agilent Technologies), and two sets of microarray analysis were performed using a Cy3 and Cy5 dye swap. After hybridization, the microarray slides were scanned with a G2505 scanner using scan control software, version A.8.4 (Agilent Technologies) and processed using Feature Extraction software (11.5.1.1; Agilent Technologies). Integration, normalization and statistical analysis of the data were performed using Subio Platform 1.16 (Subio, Tokyo, Japan), and global normalization of the ratios was performed using the Lowess method. The *P*‐values for differences in gene expression were corrected according to the Benjamini Hochberg false discovery rate (FDR) method, and probes with a *P*‐value of less than 0.05 were used for the analysis. Because the array was designed according to a former gene mode, probes that perfectly matched current gene models were used for the analysis (release 7 of the MSU Rice Genome Annotation Project). All of the microarray data are available at array express (http://www.ebi.ac.uk/arrayexpress/) under accession numbers E‐MTAB‐3756.

### Electrolyte leakage assays

Electrolyte leakage assays were performed as described previously (Lee *et al*., 2007) with modifications. One gram of rice leaves was cut into a width of 5 cm and submerged into 10 mL of sterilized water, and the samples were incubated at room temperature for 3 h with gentle rotation. The electrical conductivity of the solution with (*E*_*a*_) or without (*E*_*b*_) rice leaves was measured after incubation. Next, the samples were autoclaved, and the electrical conductivity of the solution with (*E*_1_) or without (*E*_2_) rice leaves was measured again. The relative electrolyte leakage was calculated according to the following formula: (*E*_*a*_ − *E*_*b*_)/(*E*_1_ − *E*_2_) × 100 (%).

## Supporting information


**Table S1** Yield parameters of the vector control and *DPB3‐1*‐overexpressing *Arabidopsis* under nonstress conditions.

**Table S2** Number of *cis* elements on the promoters of genes which expression levels were analysed in Figure 6.

**Table S3** Up‐regulated genes in the vector control rice under the heat stress condition.

**Table S4** Down‐regulated genes in the vector control rice under the heat stress condition.

**Table S5** Down‐regulated gene in *DPB3‐1*‐overexpressing rice under the nonstress condition.

**Table S6** Up‐regulated gene in *DPB3‐1*‐overexpressing rice under the heat stress condition.

**Table S7** Down‐regulated gene in *DPB3‐1*‐overexpressing rice under the heat stress condition.

**Table S8** GO analysis of the genes up‐regulated in the *DPB3‐1*‐overexpressing plants under the heat stress condition.

**Table S9** GO analysis of the genes down‐regulated in the *DPB3‐1*‐overexpressing plants under the heat stress condition.

**Table S10** Overrepresentation analysis of DRE, CCAAT and HSE sequences in the promoters of the top 100 up‐regulated genes in *Ubi:DPB3‐1* rice under the heat stress condition.

**Table S11** Sequences of primers used in this study.

**Figure S1** Phenotypes of the vector control and *DPB3‐1*‐overexpressing *Arabidopsis* under non‐stress conditions after ripening.
**Figure S2** Alignment of DREB2A, OsDREB2B2 and GmDREB2A;2.
**Figure S3** Additional phenotypic analysis of the *DPB3‐1*‐overexpressing rice under nonstress conditions.
**Figure S4** Plant phenotype and seed morphology of the *DPB3‐1*‐overexpressing rice after desiccation.
**Figure S5** Phylogenetic tree lf HSP20 family proteins in *Arabidopsis thaliana* and *Oryza sativa* based on amino acids sequences of the conserved domain.
**Figure S6** Proportions of abiotic stress‐repressive genes among the down‐regulated genes in the *DPB3‐1*‐overexpressing rice under heat stress condition or rice whole genome.
**Figure S7** Confirmation of down‐regulated gene expression in *Ubi:DPB3‐1* identified by microarray analysis.
**Figure S8** Microarray analysis of up‐regulated or down‐regulated genes in *Ubi:DPB3‐1* plants under heat stress conditions.
**Figure S9** Drought stress tolerance of the *DPB3‐1*‐overexpressing rice.
