# Supplementary material for: The Arabidopsis transcriptional regulator DPB3‐1 enhances heat stress tolerance without growth retardation in rice
Source: Plant Biotechnol J. 2016 Feb 3;14(8):1756–67. doi: 10.1111/pbi.12535 (PMC5067654; doi:10.1111/pbi.12535)
Supplement: Supplementary file 1 — Table S1 Yield parameters of the vector control and DPB3‐1‐overexpressing Arabidopsis under nonstress conditions. [file PBI-14-1756-s006.docx]

**Table S1** Yield parameters of the vector control and *DPB3-1*-overexpressing *Arabidopsis* under non-stress conditions.

| Traits | Vector control (%) | *35S:DPB3-1*-a (%) | *35S:DPB3-1*-b (%) | *35S:DPB3-1*-c (%) |
| --- | --- | --- | --- | --- |
| Silique number per plant | 107 ± 7 (100) | 108 ± 9 (101) | 102 ± 9 (95) | 103 ± 5 (97) |
| Seed weight per plant (mg) | 46.5 ± 5.3 (100) | 43.2 ± 5.3 (93) | 52.2 ± 4.1 (112) | 56.6 ± 5.3 (121) |
| Silique length (mm) | 11.1 ± 1.4 (100) | 10.5 ± 1.3 (95) | 10.3 ± 1.1 (93) | 10.4 ± 1.1 (94) |
| Seed number per silique | 43 ± 5 (100) | 40 ± 5 (93) | 43 ± 5 (100) | 40 ± 2 (93) |
| Seed weight per 1000 seeds (mg) | 15.1 ± 1.4 (100) | 14.6 ± 1.5 (96) | 15.8 ± 1.5 (104) | 16.1 ± 0.4 (106) |

Various parameters involved in the yield were measured. Values are the means and SD (n = 15). Relative percentages are shown in brackets. The data were evaluated using one-way ANOVA, and no significant differences were detected (P > 0.05).
