# Supplementary material for: The Arabidopsis transcriptional regulator DPB3‐1 enhances heat stress tolerance without growth retardation in rice
Source: Plant Biotechnol J. 2016 Feb 3;14(8):1756–67. doi: 10.1111/pbi.12535 (PMC5067654; doi:10.1111/pbi.12535)
Supplement: Supplementary file 2 — Table S2 Number of cis elements on the promoters of genes which expression levels were analysed in Figure 6. [file PBI-14-1756-s010.docx]

**Table S2** Number of *cis* elements on the promoters of genes which expression levels were analyzed in Figure 6.

| Gene code | Annotation | DRE | CCAAT | HSE | ABRE |
| --- | --- | --- | --- | --- | --- |
| LOC_Os02g32590 | OsHsfA3 | 1 | 0 | 0 | 1 |
| LOC_Os03g53340 | OsHsfA2a | 0 | 2 | 5 | 1 |
| LOC_Os01g39020 | OsHsfA7 | 0 | 2 | 0 | 1 |
| LOC_Os03g12370 | OsHsfA9 | 4 | 2 | 0 | 1 |
| LOC_Os01g04370 | HSP20 family | 0 | 1 | 1 | 1 |
| LOC_Os03g15960 | HSP20 family | 3 | 3 | 1 | 0 |
| LOC_Os03g16020 | HSP20 family | 0 | 1 | 2 | 2 |
| LOC_Os05g27930 | OsDREB2B | 1 | 2 | 2 | 0 |

The sequences of 1-kb promoters of each gene were obtained from Phytozome (Phytozome v10.1, http://www.phytozome.net/). The numbers of DRE (A/GCCGAC), CCAAT, HSE (GAAnnTTC; “n” means an arbitrary nucleotide) and ABRE (ACGTGG/T) on the 1-kb promoters are shown.
