# Supplementary material for: The Arabidopsis transcriptional regulator DPB3‐1 enhances heat stress tolerance without growth retardation in rice
Source: Plant Biotechnol J. 2016 Feb 3;14(8):1756–67. doi: 10.1111/pbi.12535 (PMC5067654; doi:10.1111/pbi.12535)
Supplement: Supplementary file 3 — Table S3 Up‐regulated genes in the vector control rice under the heat stress condition. [file PBI-14-1756-s009.pdf]

Table S3 Upregulated genes in the vector control rice under the heat stress condition.

[a] Description as given by the MSU 7.0 database.

MSU7_locus	Fold Change	Q-Value	Average (log2)	SD (log2)	Regulation	Description[a]
LOC_Os02g15930	207.0	0.00029	7.7	0.0	up	expressed protein
LOC_Os04g28420	128.7	0.00067	7.0	0.2	up	peptidyl-prolyl isomerase, putative, expressed
LOC_Os04g45480	70.9	0.00065	6.1	0.1	up	heat shock protein STI, putative, expressed
LOC_Os06g36930	48.7	0.00079	5.6	0.2	up	HSF-type DNA-binding domain containing protein, expressed
LOC_Os03g14180	45.9	0.00147	5.5	0.3	up	hsp20/alpha crystallin family protein, putative, expressed
LOC_Os10g28340	29.3	0.00060	4.9	0.1	up	heat stress transcription factor, putative, expressed
LOC_Os02g40900	28.6	0.00029	4.8	0.0	up	RNA recognition motif containing protein, putative, expressed
LOC_Os03g61150	28.2	0.00081	4.8	0.2	up	expressed protein
LOC_Os08g43334	28.0	0.00057	4.8	0.1	up	HSF-type DNA-binding domain containing protein, expressed
LOC_Os01g46740	26.9	0.00148	4.7	0.3	up	expressed protein
LOC_Os03g49430	25.4	0.00113	4.7	0.2	up	pre-mRNA-splicing factor, putative, expressed
LOC_Os06g36390	24.4	0.00083	4.6	0.1	up	expressed protein
LOC_Os01g42190	23.8	0.00080	4.6	0.1	up	heat shock protein DnaJ, putative, expressed
LOC_Os03g19270	22.4	0.00120	4.5	0.2	up	universal stress protein domain containing protein, putative, expressed
LOC_Os05g44340	22.0	0.00234	4.5	0.4	up	heat shock protein 101, putative, expressed
LOC_Os06g06490	20.9	0.00057	4.4	0.1	up	U-box domain containing heat shock protein, putative, expressed
LOC_Os03g18200	20.5	0.00047	4.4	0.0	up	heat shock protein DnaJ, putative, expressed
LOC_Os11g47500	18.7	0.00064	4.2	0.1	up	glycosyl hydrolase, putative, expressed
LOC_Os02g09880	18.3	0.00200	4.2	0.3	up	hypothetical protein
LOC_Os06g39240	18.3	0.00135	4.2	0.2	up	endothelial differentiation-related factor 1, putative, expressed
LOC_Os07g37280	18.2	0.00326	4.2	0.4	up	hypothetical protein
LOC_Os12g36840	18.2	0.00133	4.2	0.2	up	pathogenesis-related Bet v I family protein, putative, expressed
LOC_Os03g11910	17.5	0.00079	4.1	0.1	up	DnaK family protein, putative, expressed
LOC_Os02g54140	17.0	0.00207	4.1	0.3	up	hsp20/alpha crystallin family protein, putative, expressed
LOC_Os02g48140	16.9	0.00286	4.1	0.4	up	hsp20/alpha crystallin family protein, putative, expressed
LOC_Os06g14240	16.5	0.00235	4.0	0.3	up	hsp20/alpha crystallin family protein, putative, expressed
LOC_Os01g68960	16.5	0.00086	4.0	0.1	up	expressed protein
LOC_Os04g33115	15.8	0.00062	4.0	0.1	up	expressed protein
LOC_Os01g55270	15.6	0.00026	4.0	0.0	up	SGS domain containing protein, expressed
LOC_Os04g36750	15.6	0.00245	4.0	0.3	up	hsp20/alpha crystallin family protein, putative, expressed
LOC_Os12g05210	15.1	0.00130	3.9	0.2	up	expressed protein
LOC_Os03g51920	14.8	0.00365	3.9	0.4	up	peptidase, M50 family, putative, expressed
LOC_Os06g09560	14.6	0.00206	3.9	0.3	up	heat shock protein DnaJ, putative, expressed
LOC_Os02g08490	14.6	0.00151	3.9	0.2	up	chaperone protein clpB 1, putative, expressed
LOC_Os06g39370	14.4	0.00145	3.9	0.2	up	OsFBK16 - F-box domain and kelch repeat containing protein, expressed
LOC_Os03g16460	13.0	0.00062	3.7	0.1	up	expressed protein
LOC_Os01g12820	12.9	0.00131	3.7	0.2	up	harpin-induced protein, putative, expressed
LOC_Os08g03420	12.7	0.00067	3.7	0.1	up	kelch repeat protein, putative, expressed
LOC_Os08g05590	12.6	0.00173	3.7	0.2	up	aquaporin protein, putative, expressed
LOC_Os06g46900	12.5	0.00071	3.6	0.1	up	phosphosulfolactate synthase-related protein, putative, expressed
LOC_Os02g53250	12.4	0.00064	3.6	0.1	up	expressed protein
LOC_Os09g25945	12.3	0.00129	3.6	0.2	up	expressed protein
LOC_Os06g32650	11.9	0.00126	3.6	0.2	up	expressed protein
LOC_Os05g45020	11.6	0.00112	3.5	0.2	up	zinc finger/CCCH transcription factor, putative, expressed
LOC_Os09g33820	11.5	0.00081	3.5	0.1	up	lecithine cholesterol acyltransferase, putative, expressed
LOC_Os03g56540	11.5	0.00137	3.5	0.2	up	heat shock protein DnaJ, putative, expressed
LOC_Os08g39560	11.4	0.00200	3.5	0.3	up	OTU-like cysteine protease family protein, putative, expressed
LOC_Os06g04240	11.1	0.00114	3.5	0.2	up	expressed protein
LOC_Os03g55670	10.9	0.00095	3.4	0.1	up	expressed protein
LOC_Os01g08860	10.9	0.00924	3.4	0.7	up	hsp20/alpha crystallin family protein, putative, expressed
LOC_Os01g19820	10.8	0.00065	3.4	0.1	up	universal stress protein domain containing protein, putative, expressed
LOC_Os04g54210	10.8	0.00098	3.4	0.1	up	expressed protein
LOC_Os08g24400	10.8	0.00163	3.4	0.2	up	SWP, putative, expressed
LOC_Os01g52140	10.8	0.00088	3.4	0.1	up	expressed protein
LOC_Os03g13450	10.6	0.00071	3.4	0.1	up	expressed protein
LOC_Os11g47510	10.6	0.00074	3.4	0.1	up	glycosyl hydrolase, putative, expressed
LOC_Os01g52110	10.6	0.00204	3.4	0.3	up	RING finger and CHY zinc finger domain-containing protein 1, putative, expressed
LOC_Os06g09550	10.4	0.00090	3.4	0.1	up	expressed protein
LOC_Os09g28210	10.2	0.00200	3.4	0.3	up	bHelix-loop-helix transcription factor, putative, expressed
LOC_Os07g43740	9.8	0.00519	3.3	0.5	up	zinc finger, C3HC4 type domain containing protein, expressed
LOC_Os05g48810	9.7	0.00148	3.3	0.2	up	dnaJ domain containing protein, expressed
LOC_Os02g34250	9.7	0.00151	3.3	0.2	up	expressed protein
LOC_Os06g06400	9.6	0.00173	3.3	0.2	up	NBS-LRR type disease resistance protein, putative, expressed
LOC_Os03g02300	9.5	0.00062	3.2	0.1	up	expressed protein
LOC_Os03g60560	9.3	0.00095	3.2	0.1	up	ZOS3-21 - C2H2 zinc finger protein, expressed
LOC_Os02g32770	9.3	0.00464	3.2	0.4	up	cytochrome P450, putative, expressed
LOC_Os11g15060	9.1	0.00099	3.2	0.1	up	SAM dependent carboxyl methyltransferase, putative, expressed
LOC_Os02g04650	9.1	0.00057	3.2	0.1	up	activator of 90 kDa heat shock protein ATPase homolog, putative, expressed
LOC_Os01g21120	9.1	0.00062	3.2	0.1	up	AP2 domain containing protein, expressed
LOC_Os06g03520	9.0	0.00179	3.2	0.2	up	DUF581 domain containing protein, expressed
LOC_Os04g58280	8.6	0.00192	3.1	0.2	up	stem-specific protein TSJT1, putative, expressed
LOC_Os03g55770	8.5	0.00086	3.1	0.1	up	expressed protein
LOC_Os03g58890	8.5	0.00121	3.1	0.1	up	oxidoreductase, putative, expressed
LOC_Os02g49720	8.3	0.01154	3.1	0.7	up	aldehyde dehydrogenase, putative, expressed
LOC_Os05g45830	8.2	0.00108	3.0	0.1	up	expressed protein
LOC_Os01g65830	8.2	0.00037	3.0	0.0	up	acyl-desaturase, chloroplast precursor, putative, expressed
LOC_Os01g67150	8.2	0.00057	3.0	0.0	up	expressed protein
LOC_Os03g08460	8.1	0.00101	3.0	0.1	up	AP2 domain containing protein, expressed
LOC_Os05g43040	8.1	0.00071	3.0	0.1	up	tetratricopeptide repeat domain containing protein, expressed
LOC_Os04g55170	8.1	0.00132	3.0	0.2	up	LTPL126 - Protease inhibitor/seed storage/LTP family protein precursor, expressed
LOC_Os02g43330	8.1	0.00124	3.0	0.1	up	homeobox associated leucine zipper, putative, expressed
LOC_Os03g60780	8.0	0.00069	3.0	0.1	up	armadillo/beta-catenin-like repeat containing protein, expressed
LOC_Os11g31060	8.0	0.00394	3.0	0.4	up	IQ calmodulin-binding and BAG domain containing protein, putative, expressed
LOC_Os08g37470	8.0	0.00349	3.0	0.3	up	2-aminoethanethiol dioxygenase, putative, expressed
LOC_Os09g23595	7.9	0.00179	3.0	0.2	up	expressed protein
LOC_Os06g05420	7.8	0.00089	3.0	0.1	up	expressed protein
LOC_Os08g40690	7.8	0.00090	3.0	0.1	up	glycosyl hydrolase, putative, expressed
LOC_Os07g02330	7.8	0.00158	3.0	0.2	up	protein phosphatase 2C, putative, expressed
LOC_Os06g05010	7.7	0.00128	2.9	0.1	up	early nodulin 93 ENOD93 protein, putative, expressed
LOC_Os07g09630	7.6	0.00128	2.9	0.1	up	oxidoreductase, putative, expressed
LOC_Os02g57280	7.6	0.00057	2.9	0.0	up	CBS domain containing membrane protein, putative, expressed
LOC_Os10g23050	7.6	0.00079	2.9	0.1	up	helix-loop-helix DNA-binding protein, putative, expressed
LOC_Os11g15040	7.5	0.00142	2.9	0.2	up	S-adenosyl-L-methionine:benzoic acid/salicylic acid carboxyl methyltransferase, putative, expressed
LOC_Os08g09690	7.5	0.00116	2.9	0.1	up	nuclear transcription factor Y subunit, putative, expressed
LOC_Os05g39250	7.5	0.00278	2.9	0.3	up	phosphatidylethanolamine-binding protein, putative, expressed
LOC_Os10g31850	7.5	0.00114	2.9	0.1	up	RING finger and CHY zinc finger domain-containing protein 1, putative, expressed
LOC_Os08g04740	7.4	0.00237	2.9	0.2	up	expressed protein
LOC_Os08g36920	7.3	0.00071	2.9	0.1	up	AP2 domain containing protein, expressed
LOC_Os03g18870	7.3	0.00190	2.9	0.2	up	heat shock protein DnaJ, putative, expressed
LOC_Os12g40330	7.3	0.00148	2.9	0.2	up	expressed protein
LOC_Os06g49640	7.2	0.00111	2.9	0.1	up	uncharacterized UPF0114 domain containing protein, expressed
LOC_Os05g28210	7.2	0.00296	2.8	0.3	up	small hydrophilic plant seed protein, putative, expressed

LOC_Os02g38386	7.1	0.00204	2.8	0.2	up	NBS-LRR disease resistance protein, putative, expressed
LOC_Os02g06720	7.1	0.00240	2.8	0.2	up	WD domain containing protein, putative, expressed
LOC_Os03g12820	7.0	0.00057	2.8	0.0	up	ATP8, putative, expressed
LOC_Os05g03910	7.0	0.00139	2.8	0.2	up	RNA polymerase II-associated protein 3, putative, expressed
LOC_Os08g32930	7.0	0.00185	2.8	0.2	up	expressed protein
LOC_Os07g47630	6.9	0.00064	2.8	0.1	up	RNA recognition motif containing protein, expressed
LOC_Os10g28440	6.8	0.00227	2.8	0.2	up	sulfate transporter 3.1, putative, expressed
LOC_Os08g39150	6.8	0.00047	2.8	0.0	up	expressed protein
LOC_Os01g72370	6.7	0.00070	2.7	0.1	up	helix-loop-helix DNA-binding domain containing protein, expressed
LOC_Os03g25770	6.7	0.00094	2.7	0.1	up	RNA recognition motif containing protein, putative, expressed
LOC_Os02g09810	6.7	0.00065	2.7	0.1	up	amino acid transporter, putative, expressed
LOC_Os09g33800	6.7	0.00188	2.7	0.2	up	arabinogalactan protein, putative, expressed
LOC_Os03g63950	6.6	0.00065	2.7	0.1	up	plastid-specific 30S ribosomal protein 1, chloroplast precursor, putative, expressed
LOC_Os07g16364	6.6	0.00262	2.7	0.2	up	expressed protein
LOC_Os09g04160	6.5	0.00468	2.7	0.4	up	expressed protein
LOC_Os03g26460	6.3	0.00108	2.7	0.1	up	CS domain containing protein, putative, expressed
LOC_Os02g53320	6.2	0.00062	2.6	0.1	up	universal stress protein domain containing protein, putative, expressed
LOC_Os01g58960	6.2	0.00055	2.6	0.0	up	cytochrome P450, putative, expressed
LOC_Os08g23754	6.2	0.00098	2.6	0.1	up	expressed protein
LOC_Os01g50400	6.2	0.00213	2.6	0.2	up	STE_MEKK_ste11_MAP3K.5 - STE kinases include homologs to sterile 7, sterile 11 and sterile 20 from yeast, expressed
LOC_Os01g47840	6.2	0.00245	2.6	0.2	up	S-locus-like receptor protein kinase, putative, expressed
LOC_Os06g48500	6.2	0.00139	2.6	0.1	up	expressed protein
LOC_Os12g05200	6.2	0.00088	2.6	0.1	up	DNA binding protein, putative, expressed
LOC_Os05g13300	6.1	0.00166	2.6	0.2	up	helicase, putative, expressed
LOC_Os01g62970	6.1	0.00154	2.6	0.2	up	expressed protein
LOC_Os03g30300	6.0	0.00115	2.6	0.1	up	6-phosphogluconolactonase, putative, expressed
LOC_Os05g37060	6.0	0.00167	2.6	0.2	up	MYB family transcription factor, putative, expressed
LOC_Os01g38530	6.0	0.00071	2.6	0.1	up	ELF3 protein, putative, expressed
LOC_Os03g47860	5.9	0.00197	2.6	0.2	up	transferase family protein, putative, expressed
LOC_Os06g15810	5.9	0.00070	2.6	0.1	up	integral membrane protein, putative, expressed
LOC_Os08g41400	5.8	0.00104	2.5	0.1	up	expressed protein
LOC_Os09g26840	5.8	0.00097	2.5	0.1	up	expressed protein
LOC_Os12g35610	5.8	0.00091	2.5	0.1	up	respiratory burst oxidase, putative, expressed
LOC_Os04g05010	5.8	0.00104	2.5	0.1	up	CBS domain containing membrane protein, putative, expressed
LOC_Os12g24020	5.8	0.00029	2.5	0.0	up	rhodanese-like domain containing protein, putative, expressed
LOC_Os05g27780	5.7	0.00412	2.5	0.3	up	expressed protein
LOC_Os06g04220	5.7	0.00071	2.5	0.1	up	expressed protein
LOC_Os04g09580	5.7	0.00440	2.5	0.3	up	expressed protein
LOC_Os01g12460	5.7	0.00173	2.5	0.2	up	expressed protein
LOC_Os03g19250	5.7	0.00310	2.5	0.3	up	AMP-binding enzyme, putative, expressed
LOC_Os09g35030	5.6	0.00097	2.5	0.1	up	dehydration-responsive element-binding protein, putative, expressed
LOC_Os12g42860	5.6	0.00126	2.5	0.1	up	2-aminoethanethiol dioxygenase, putative, expressed
LOC_Os02g44770	5.6	0.00103	2.5	0.1	up	uncharacterized mscS family protein, putative, expressed
LOC_Os04g49370	5.6	0.00071	2.5	0.1	up	expressed protein
LOC_Os04g48350	5.6	0.00083	2.5	0.1	up	dehydration-responsive element-binding protein, putative, expressed
LOC_Os01g63690	5.6	0.00352	2.5	0.3	up	hs1, putative, expressed
LOC_Os05g33960	5.6	0.00088	2.5	0.1	up	peptide transporter PTR2, putative, expressed
LOC_Os07g46700	5.5	0.00073	2.5	0.1	up	zinc finger, RING-type, putative, expressed
LOC_Os09g23595	5.5	0.00060	2.5	0.0	up	expressed protein
LOC_Os09g11480	5.5	0.00098	2.5	0.1	up	AP2 domain containing protein, expressed
LOC_Os07g41310	5.5	0.00069	2.5	0.1	up	COBRA, putative, expressed
LOC_Os01g47190	5.5	0.00185	2.5	0.2	up	phosphoglycerate mutase, putative, expressed
LOC_Os02g44990	5.4	0.00096	2.4	0.1	up	OsFBDUF13 - F-box and DUF domain containing protein, expressed
LOC_Os05g29810	5.4	0.00216	2.4	0.2	up	AP2 domain containing protein, expressed
LOC_Os02g43020	5.4	0.00092	2.4	0.1	up	heat shock protein STI, putative, expressed
LOC_Os01g69840	5.4	0.00103	2.4	0.1	up	expressed protein
LOC_Os09g39090	5.4	0.00278	2.4	0.2	up	vignain precursor, putative, expressed
LOC_Os05g34510	5.4	0.00136	2.4	0.1	up	expressed protein
LOC_Os01g56740	5.3	0.00242	2.4	0.2	up	OsFBDUF7 - F-box and DUF domain containing protein, expressed
LOC_Os03g16960	5.3	0.00148	2.4	0.1	up	cysteine-rich repeat secretory protein 55 precursor, putative, expressed
LOC_Os06g48500	5.2	0.00356	2.4	0.3	up	expressed protein
LOC_Os01g06740	5.2	0.00163	2.4	0.2	up	ribosome inactivating protein, putative, expressed
LOC_Os05g34530	5.2	0.00258	2.4	0.2	up	auxin-inducible protein, putative, expressed
LOC_Os01g64120	5.2	0.00057	2.4	0.0	up	2Fe-2S iron-sulfur cluster binding domain containing protein, expressed
LOC_Os02g32590	5.1	0.00145	2.4	0.1	up	HSF-type DNA-binding domain containing protein, expressed
LOC_Os01g66544	5.1	0.00069	2.4	0.1	up	expressed protein
LOC_Os05g07940	5.1	0.00395	2.3	0.3	up	glyoxalase family protein, putative, expressed
LOC_Os04g31760	5.1	0.00230	2.3	0.2	up	expressed protein
LOC_Os01g10640	5.0	0.00185	2.3	0.2	up	expressed protein
LOC_Os03g20870	5.0	0.00251	2.3	0.2	up	zinc finger, C3HC4 type domain containing protein, expressed
LOC_Os05g11140	5.0	0.00065	2.3	0.1	up	CK1_CaseinKinase_1a.5 - CK1 includes the casein kinase 1 kinases, expressed
LOC_Os02g33070	4.9	0.00176	2.3	0.2	up	expressed protein
LOC_Os10g26150	4.9	0.00288	2.3	0.2	up	expressed protein
LOC_Os05g38040	4.9	0.00067	2.3	0.1	up	expressed protein
LOC_Os08g17680	4.9	0.00102	2.3	0.1	up	stromal cell-derived factor 2-like protein precursor, putative, expressed
LOC_Os03g17200	4.9	0.00102	2.3	0.1	up	plant-specific domain TIGR01589 family protein, expressed
LOC_Os06g12530	4.8	0.00184	2.3	0.2	up	CS domain containing protein, putative, expressed
LOC_Os03g50870	4.8	0.00069	2.3	0.1	up	expressed protein
LOC_Os03g04080	4.8	0.00535	2.3	0.3	up	expressed protein
LOC_Os09g26500	4.8	0.00244	2.3	0.2	up	CXE carboxylesterase, putative, expressed
LOC_Os03g19290	4.8	0.00274	2.3	0.2	up	mitochondrial import inner membrane translocase subunit Tim17, putative, expressed
LOC_Os05g38710	4.8	0.00207	2.3	0.2	up	lipin, N-terminal conserved region family protein, expressed
LOC_Os03g07180	4.8	0.00162	2.2	0.1	up	embryonic protein DC-8, putative, expressed
LOC_Os03g46052	4.7	0.00740	2.2	0.4	up	expressed protein
LOC_Os12g08550	4.7	0.00326	2.2	0.2	up	expressed protein
LOC_Os03g57340	4.7	0.00120	2.2	0.1	up	chaperone protein dnaJ, putative, expressed
LOC_Os10g13700	4.7	0.00110	2.2	0.1	up	phosphoenolpyruvate carboxykinase, putative, expressed
LOC_Os01g64360	4.7	0.00114	2.2	0.1	up	MYB family transcription factor, putative, expressed
LOC_Os08g36150	4.7	0.00079	2.2	0.1	up	activator of 90 kDa heat shock protein ATPase homolog, putative, expressed
LOC_Os05g48200	4.7	0.00159	2.2	0.1	up	glutamate synthase, chloroplast precursor, putative, expressed
LOC_Os06g39260	4.6	0.00060	2.2	0.0	up	solute carrier family 35 member B1, putative, expressed
LOC_Os07g43160	4.6	0.00135	2.2	0.1	up	uncharacterized glycosyl hydrolase Rv2006/MT2062, putative, expressed
LOC_Os07g43950	4.5	0.00079	2.2	0.1	up	RNA recognition motif containing protein, putative, expressed
LOC_Os02g28980	4.5	0.00071	2.2	0.1	up	peptidyl-prolyl isomerase, putative, expressed
LOC_Os05g41220	4.5	0.00057	2.2	0.0	up	SNF1-related protein kinase regulatory subunit beta-1, putative, expressed
LOC_Os07g39860	4.5	0.00074	2.2	0.1	up	expressed protein
LOC_Os03g60080	4.5	0.00422	2.2	0.3	up	NAC domain-containing protein 67, putative, expressed
LOC_Os09g27830	4.5	0.00110	2.2	0.1	up	OsPDIL2-3 protein disulfide isomerase PDIL2-3, expressed
LOC_Os01g13760	4.5	0.00231	2.2	0.2	up	dnaJ domain containing protein, expressed
LOC_Os01g37470	4.4	0.00070	2.2	0.1	up	asp/Glu racemase, putative, expressed
LOC_Os01g53730	4.4	0.00153	2.2	0.1	up	expressed protein
LOC_Os02g38392	4.4	0.00214	2.1	0.2	up	NBS-LRR disease resistance protein, putative, expressed
LOC_Os01g32780	4.4	0.00060	2.1	0.0	up	universal stress protein domain containing protein, putative, expressed
LOC_Os05g38264	4.4	0.00057	2.1	0.0	up	expressed protein
LOC_Os03g51010	4.4	0.00137	2.1	0.1	up	hydrolase, alpha/beta fold family domain containing protein, expressed

LOC_Os02g34580	4.3	0.00434	2.1	0.3	up	ammonium transporter protein, putative, expressed
LOC_Os03g26870	4.3	0.00259	2.1	0.2	up	WD-40 repeat family protein, putative, expressed
LOC_Os09g23350	4.3	0.00408	2.1	0.3	up	trehalose synthase, putative, expressed
LOC_Os10g37640	4.3	0.00225	2.1	0.2	up	HIT zinc finger domain containing protein, expressed
LOC_Os05g38710	4.3	0.00126	2.1	0.1	up	lipin, N-terminal conserved region family protein, expressed
LOC_Os07g44950	4.2	0.00086	2.1	0.1	up	bZIP transcription factor domain containing protein, expressed
LOC_Os12g31860	4.2	0.00170	2.1	0.1	up	ureide permease, putative, expressed
LOC_Os06g21330	4.2	0.00093	2.1	0.1	up	ABB1 - Ankyrin repeat region with 2 Bric-a-Brac, Tramtrack, Broad Complex BTB domains, expressed
LOC_Os01g56470	4.2	0.00083	2.1	0.1	up	mal d 1-associated protein, putative, expressed
LOC_Os03g03980	4.2	0.00185	2.1	0.1	up	coiled-coil domain-containing protein 124, putative, expressed
LOC_Os07g34570	4.2	0.00095	2.1	0.1	up	FAD dependent oxidoreductase domain containing protein, expressed
LOC_Os09g31482	4.2	0.00113	2.1	0.1	up	splicing factor U2AF, putative, expressed
LOC_Os01g08560	4.2	0.00077	2.1	0.1	up	DnaK family protein, putative, expressed
LOC_Os06g50230	4.2	0.00359	2.1	0.2	up	expressed protein
LOC_Os06g04390	4.2	0.00247	2.1	0.2	up	expressed protein
LOC_Os03g31300	4.2	0.00101	2.1	0.1	up	chaperone protein clpB 1, putative, expressed
LOC_Os07g35330	4.2	0.00200	2.1	0.2	up	TKL_IRAK_DUF26-lc.13 - DUF26 kinases have homology to DUF26 containing loci, expressed
LOC_Os08g37660	4.1	0.00138	2.1	0.1	up	plastocyanin-like domain containing protein, putative, expressed
LOC_Os01g43540	4.1	0.00160	2.1	0.1	up	suppressor of G2 allele of SKP1, putative, expressed
LOC_Os04g57760	4.1	0.00071	2.0	0.1	up	expressed protein
LOC_Os12g09540	4.1	0.00145	2.0	0.1	up	phosphoribosylamine--glycine ligase, putative, expressed
LOC_Os09g29840	4.1	0.00079	2.0	0.1	up	heat shock protein, putative, expressed
LOC_Os03g45250	4.1	0.00201	2.0	0.2	up	2-aminoethanethiol dioxygenase, putative, expressed
LOC_Os04g34150	4.1	0.00157	2.0	0.1	up	expressed protein
LOC_Os08g37456	4.1	0.00459	2.0	0.3	up	flavonol synthase/flavanone 3-hydroxylase, putative, expressed
LOC_Os08g02460	4.1	0.00062	2.0	0.0	up	expressed protein
LOC_Os02g54820	4.1	0.00439	2.0	0.3	up	trehalose-6-phosphate synthase, putative, expressed
LOC_Os03g59180	4.0	0.00067	2.0	0.0	up	expressed protein
LOC_Os04g41260	4.0	0.00253	2.0	0.2	up	amine oxidase, flavin-containing, domain containing protein, expressed
LOC_Os01g70570	4.0	0.00161	2.0	0.1	up	acyltransferase, putative, expressed
LOC_Os09g31478	4.0	0.00237	2.0	0.2	up	auxin efflux carrier component, putative, expressed
LOC_Os08g15460	4.0	0.00326	2.0	0.2	up	preprotein translocase subunit secY, putative, expressed
LOC_Os03g55320	4.0	0.00135	2.0	0.1	up	protein phosphatase 2C, putative, expressed
LOC_Os01g41770	4.0	0.00677	2.0	0.3	up	leucine rich repeat protein, putative, expressed
LOC_Os11g18830	4.0	0.00065	2.0	0.0	up	survival motor neuron containing protein, expressed
LOC_Os02g45450	3.9	0.00473	2.0	0.3	up	dehydration-responsive element-binding protein, putative, expressed
LOC_Os03g22050	3.9	0.00121	2.0	0.1	up	CAMK_KIN1/SNF1/Nim1_like.16 - CAMK includes calcium/calmodulin depedent protein kinases, expressed
LOC_Os06g13580	3.9	0.00290	2.0	0.2	up	expressed protein
LOC_Os01g34080	3.9	0.00060	2.0	0.0	up	armadillo/beta-catenin-like repeat family protein, expressed
LOC_Os03g55290	3.9	0.00538	2.0	0.3	up	GASR3 - Gibberellin-regulated GASA/GAST/Snakin family protein precursor, expressed
LOC_Os03g41060	3.9	0.00065	2.0	0.0	up	GASR2 - Gibberellin-regulated GASA/GAST/Snakin family protein precursor, putative, expressed
LOC_Os02g01540	3.9	0.00142	2.0	0.1	up	ribosomal protein, putative, expressed
LOC_Os05g14370	3.9	0.00467	1.9	0.3	up	WRKY82, expressed
LOC_Os01g65920	3.8	0.00391	1.9	0.2	up	F-box/LRR-repeat protein 2, putative, expressed
LOC_Os02g52010	3.8	0.00272	1.9	0.2	up	phosphate-induced protein 1 conserved region domain containing protein, expressed
LOC_Os12g40180	3.8	0.00213	1.9	0.2	up	expressed protein
LOC_Os01g59180	3.8	0.00219	1.9	0.2	up	OsFBX27 - F-box domain containing protein, expressed
LOC_Os03g50130	3.8	0.00265	1.9	0.2	up	microsomal glutathione S-transferase 3, putative, expressed
LOC_Os09g26880	3.8	0.00177	1.9	0.1	up	aldehyde dehydrogenase, putative, expressed
LOC_Os01g06876	3.8	0.00410	1.9	0.2	up	Cf-2, putative, expressed
LOC_Os01g48250	3.8	0.00216	1.9	0.2	up	OsFBDUF5 - F-box and DUF domain containing protein, expressed
LOC_Os10g28350	3.8	0.00115	1.9	0.1	up	1,2-dihydroxy-3-keto-5-methylthiopentene dioxygenase protein, putative, expressed
LOC_Os04g38320	3.8	0.00117	1.9	0.1	up	expressed protein
LOC_Os07g30300	3.8	0.00057	1.9	0.0	up	small G protein family protein, putative, expressed
LOC_Os05g30140	3.8	0.00128	1.9	0.1	up	RNA recognition motif containing protein, expressed
LOC_Os06g28194	3.8	0.00353	1.9	0.2	up	expressed protein
LOC_Os04g40990	3.8	0.00290	1.9	0.2	up	malate synthase, glyoxysomal, putative, expressed
LOC_Os09g31180	3.8	0.00186	1.9	0.1	up	ribosomal protein L6, putative, expressed
LOC_Os01g01340	3.8	0.00163	1.9	0.1	up	light-induced protein 1-like, putative, expressed
LOC_Os01g60600	3.8	0.00969	1.9	0.4	up	WRKY108, expressed
LOC_Os05g49164	3.8	0.00383	1.9	0.2	up	expressed protein
LOC_Os04g29680	3.8	0.00172	1.9	0.1	up	OsWAK38 - OsWAK receptor-like protein kinase, expressed
LOC_Os04g27190	3.7	0.00128	1.9	0.1	up	terpene synthase, putative, expressed
LOC_Os06g02620	3.7	0.00065	1.9	0.0	up	chaperone protein dnaJ, putative, expressed
LOC_Os06g50300	3.7	0.00173	1.9	0.1	up	heat shock protein, putative, expressed
LOC_Os02g43360	3.7	0.00188	1.9	0.1	up	cytochrome b5-like Heme/Steroid binding domain containing protein, expressed
LOC_Os06g41770	3.7	0.00047	1.9	0.0	up	bZIP transcription factor domain containing protein, expressed
LOC_Os11g02080	3.7	0.00071	1.9	0.1	up	expressed protein
LOC_Os05g07940	3.7	0.00348	1.9	0.2	up	glyoxalase family protein, putative, expressed
LOC_Os09g27330	3.7	0.00247	1.9	0.2	up	oxidoreductase/ transition metal ion binding protein, putative, expressed
LOC_Os03g05750	3.7	0.01425	1.9	0.5	up	heavy-metal-associated domain-containing protein, putative, expressed
LOC_Os01g15000	3.7	0.00333	1.9	0.2	up	lipase, putative, expressed
LOC_Os06g50300	3.7	0.00743	1.9	0.3	up	heat shock protein, putative, expressed
LOC_Os07g37400	3.7	0.00114	1.9	0.1	up	OsFBX257 - F-box domain containing protein, expressed
LOC_Os07g41280	3.7	0.00154	1.9	0.1	up	6-phosphogluconolactonase, putative, expressed
LOC_Os06g48200	3.7	0.00554	1.9	0.3	up	glycosyl hydrolases family 16, putative, expressed
LOC_Os11g14544	3.7	0.00547	1.9	0.3	up	expressed protein
LOC_Os07g03260	3.7	0.00108	1.9	0.1	up	CSLC10 - cellulose synthase-like family C, expressed
LOC_Os07g08840	3.7	0.00130	1.9	0.1	up	thioredoxin, putative, expressed
LOC_Os02g02410	3.7	0.00553	1.9	0.3	up	DnaK family protein, putative, expressed
LOC_Os10g35460	3.7	0.00276	1.9	0.2	up	COBRA, putative, expressed
LOC_Os03g51610	3.7	0.00170	1.9	0.1	up	Inositol 1, 3, 4-trisphosphate 5/6-kinase, putative, expressed
LOC_Os02g44090	3.7	0.00694	1.9	0.3	up	zinc finger protein, putative, expressed
LOC_Os07g40240	3.6	0.00064	1.9	0.0	up	GASR9 - Gibberellin-regulated GASA/GAST/Snakin family protein precursor, expressed
LOC_Os01g38530	3.6	0.00122	1.9	0.1	up	ELF3 protein, putative, expressed
LOC_Os01g64730	3.6	0.00064	1.9	0.0	up	bZIP transcription factor domain containing protein, expressed
LOC_Os01g61990	3.6	0.00071	1.9	0.1	up	ankyrin repeat-containing protein, putative, expressed
LOC_Os06g09820	3.6	0.00311	1.9	0.2	up	cupin superfamily protein, putative, expressed
LOC_Os03g38745	3.6	0.00047	1.9	0.0	up	serine/arginine repetitive matrix protein 1, putative, expressed
LOC_Os07g38230	3.6	0.00077	1.9	0.1	up	expressed protein
LOC_Os02g11790	3.6	0.00091	1.9	0.1	up	OsFBK4 - F-box domain and kelch repeat containing protein, expressed
LOC_Os02g12380	3.6	0.00095	1.9	0.1	up	histone deacetylase, putative, expressed
LOC_Os09g27670	3.6	0.00121	1.9	0.1	up	expressed protein
LOC_Os03g04020	3.6	0.00263	1.9	0.2	up	expansin precursor, putative, expressed
LOC_Os07g05365	3.6	0.00093	1.9	0.1	up	photosystem II 10 kDa polypeptide, chloroplast precursor, putative, expressed
LOC_Os02g52210	3.6	0.00377	1.9	0.2	up	zinc finger, C3HC4 type domain containing protein, expressed
LOC_Os01g08700	3.6	0.00230	1.8	0.2	up	GIGANTEA, putative, expressed
LOC_Os02g37250	3.6	0.00079	1.8	0.1	up	hypothetical protein
LOC_Os09g27680	3.5	0.00079	1.8	0.1	up	expressed protein
LOC_Os04g45270	3.5	0.00185	1.8	0.1	up	aspartyl protease family protein, putative, expressed
LOC_Os02g39884	3.5	0.00197	1.8	0.1	up	expressed protein
LOC_Os03g59225	3.5	0.00164	1.8	0.1	up	expressed protein
LOC_Os10g30410	3.5	0.00562	1.8	0.3	up	cytochrome P450 71D7, putative, expressed
LOC_Os10g42190	3.5	0.00204	1.8	0.1	up	leucine rich repeat containing protein, expressed
LOC_Os06g49020	3.5	0.00944	1.8	0.4	up	26S proteasome non-ATPase regulatory subunit 14, putative, expressed

LOC_Os10g39640	3.5	0.00384	1.8	0.2	up	expansin precursor, putative, expressed
LOC_Os02g56900	3.5	0.00098	1.8	0.1	up	thioredoxin family protein, putative, expressed
LOC_Os01g52900	3.5	0.00357	1.8	0.2	up	expressed protein
LOC_Os01g46600	3.5	0.00199	1.8	0.1	up	seed maturation protein PM41, putative, expressed
LOC_Os06g22620	3.5	0.00225	1.8	0.1	up	hypothetical protein
LOC_Os03g56160	3.5	0.00348	1.8	0.2	up	lectin-like receptor kinase 7, putative, expressed
LOC_Os09g28500	3.5	0.00575	1.8	0.3	up	EF hand family protein, putative, expressed
LOC_Os07g49114	3.4	0.00875	1.8	0.3	up	wound-induced protein WI12, putative, expressed
LOC_Os04g34530	3.4	0.00202	1.8	0.1	up	integral membrane protein DUF6 containing protein, expressed
LOC_Os01g64430	3.4	0.00408	1.8	0.2	up	DUF623 domain containing protein, expressed
LOC_Os03g25400	3.4	0.00057	1.8	0.0	up	kinase, putative, expressed
LOC_Os02g04860	3.4	0.00173	1.8	0.1	up	expressed protein
LOC_Os09g25934	3.4	0.00101	1.8	0.1	up	expressed protein
LOC_Os05g51420	3.4	0.00173	1.8	0.1	up	hypersensitive-induced response protein, putative, expressed
LOC_Os02g55610	3.4	0.00548	1.8	0.3	up	serine-rich protein, putative, expressed
LOC_Os04g38400	3.4	0.00359	1.8	0.2	up	ethylene-insensitive 3, putative, expressed
LOC_Os06g03570	3.4	0.00093	1.8	0.1	up	pentatricopeptide, putative, expressed
LOC_Os05g08860	3.4	0.00292	1.8	0.2	up	expressed protein
LOC_Os12g32986	3.4	0.00301	1.8	0.2	up	heat shock protein, putative, expressed
LOC_Os05g35970	3.4	0.00110	1.8	0.1	up	expressed protein
LOC_Os06g05440	3.4	0.00200	1.8	0.1	up	expressed protein
LOC_Os09g25070	3.4	0.00207	1.8	0.1	up	WRKY62, expressed
LOC_Os03g06390	3.4	0.00194	1.8	0.1	up	expressed protein
LOC_Os02g53500	3.4	0.00250	1.8	0.2	up	RFC5 - Putative clamp loader of PCNA, replication factor C subunit 5, expressed
LOC_Os01g22900	3.4	0.00297	1.8	0.2	up	neutral/alkaline invertase, putative, expressed
LOC_Os08g38020	3.4	0.00124	1.7	0.1	up	bZIP transcription factor domain containing protein, expressed
LOC_Os07g49140	3.4	0.00072	1.7	0.0	up	expressed protein
LOC_Os08g34320	3.4	0.00574	1.7	0.3	up	expressed protein
LOC_Os01g67420	3.4	0.00174	1.7	0.1	up	lipase, putative, expressed
LOC_Os09g03810	3.4	0.00247	1.7	0.2	up	expressed protein
LOC_Os02g33380	3.3	0.00091	1.7	0.1	up	pectinesterase inhibitor domain containing protein, putative, expressed
LOC_Os01g12464	3.3	0.00117	1.7	0.1	up	CHCH domain containing protein, expressed
LOC_Os01g68300	3.3	0.00111	1.7	0.1	up	expressed protein
LOC_Os02g54130	3.3	0.00094	1.7	0.1	up	heat shock protein DnaJ, putative, expressed
LOC_Os03g49820	3.3	0.00704	1.7	0.3	up	expressed protein
LOC_Os02g10310	3.3	0.00154	1.7	0.1	up	fumarylacetoacetase, putative, expressed
LOC_Os01g71140	3.3	0.00169	1.7	0.1	up	xylanase inhibitor, putative, expressed
LOC_Os04g02530	3.3	0.00225	1.7	0.1	up	expressed protein
LOC_Os09g28390	3.3	0.00179	1.7	0.1	up	cytochrome P450, putative, expressed
LOC_Os01g16170	3.3	0.00473	1.7	0.2	up	PQ loop repeat domain containing protein, expressed
LOC_Os07g05360	3.3	0.00079	1.7	0.1	up	photosystem II 10 kDa polypeptide, chloroplast precursor, putative, expressed
LOC_Os02g52270	3.3	0.00067	1.7	0.0	up	heat shock protein DnaJ, putative, expressed
LOC_Os08g25690	3.3	0.00330	1.7	0.2	up	expressed protein
LOC_Os12g40780	3.3	0.00238	1.7	0.1	up	ankyrin repeat domain-containing protein 44, putative, expressed
LOC_Os11g34690	3.3	0.00130	1.7	0.1	up	expressed protein
LOC_Os10g32550	3.3	0.00288	1.7	0.2	up	T-complex protein, putative, expressed
LOC_Os12g14540	3.3	0.00185	1.7	0.1	up	expressed protein
LOC_Os03g52340	3.3	0.00102	1.7	0.1	up	endoplasmic oxidoreductin-1 precursor, putative, expressed
LOC_Os08g26870	3.3	0.00057	1.7	0.0	up	wound responsive protein, putative, expressed
LOC_Os11g14140	3.3	0.00586	1.7	0.3	up	OsFBK25 - F-box domain and kelch repeat containing protein, expressed
LOC_Os10g35070	3.3	0.00200	1.7	0.1	up	alpha-galactosidase precursor, putative, expressed
LOC_Os02g12360	3.3	0.00130	1.7	0.1	up	nuclear protein ZAP-related, putative, expressed
LOC_Os06g51260	3.3	0.00091	1.7	0.1	up	MYB family transcription factor, putative, expressed
LOC_Os01g68770	3.3	0.00421	1.7	0.2	up	selenium-binding protein, putative, expressed
LOC_Os04g53830	3.3	0.00230	1.7	0.1	up	3-beta hydroxysteroid dehydrogenase/isomerase family protein, putative, expressed
LOC_Os01g09030	3.3	0.00162	1.7	0.1	up	2-aminoethanethiol dioxygenase, putative, expressed
LOC_Os06g16060	3.3	0.00202	1.7	0.1	up	zinc finger, C3HC4 type domain containing protein, expressed
LOC_Os03g21210	3.3	0.00180	1.7	0.1	up	endoglucanase, putative, expressed
LOC_Os05g06440	3.2	0.00277	1.7	0.2	up	dnaJ homolog subfamily B member 11 precursor, putative, expressed
LOC_Os04g44820	3.2	0.00169	1.7	0.1	up	zinc finger, C3HC4 type domain containing protein, expressed
LOC_Os11g44940	3.2	0.00164	1.7	0.1	up	seed maturation protein PM27, putative, expressed
LOC_Os01g29330	3.2	0.00388	1.7	0.2	up	expressed protein
LOC_Os03g47500	3.2	0.00083	1.7	0.1	up	zinc finger, C3HC4 type domain containing protein, expressed
LOC_Os06g11660	3.2	0.00680	1.7	0.3	up	phosphate-induced protein 1 conserved region domain containing protein, expressed
LOC_Os11g40249	3.2	0.00067	1.7	0.0	up	expressed protein
LOC_Os11g37230	3.2	0.00077	1.7	0.0	up	zinc finger, C3HC4 type domain containing protein, expressed
LOC_Os07g49470	3.2	0.00529	1.7	0.2	up	protein kinase APK1B, chloroplast precursor, putative, expressed
LOC_Os03g03034	3.2	0.00333	1.7	0.2	up	flavonol synthase/flavanone 3-hydroxylase, putative, expressed
LOC_Os06g49030	3.2	0.00128	1.7	0.1	up	activator of 90 kDa heat shock protein ATPase homolog, putative, expressed
LOC_Os03g15720	3.2	0.00172	1.7	0.1	up	expressed protein
LOC_Os01g15270	3.2	0.00057	1.7	0.0	up	expressed protein
LOC_Os01g48940	3.2	0.00269	1.7	0.2	up	expressed protein
LOC_Os01g13570	3.2	0.00029	1.7	0.0	up	phosphoglycerate mutase, putative, expressed
LOC_Os01g16140	3.2	0.00107	1.7	0.1	up	expressed protein
LOC_Os06g48160	3.2	0.00365	1.7	0.2	up	glycosyl hydrolases family 16, putative, expressed
LOC_Os02g13370	3.2	0.00295	1.7	0.2	up	expressed protein
LOC_Os12g11840	3.2	0.00353	1.7	0.2	up	hypothetical protein
LOC_Os08g38880	3.2	0.00292	1.7	0.2	up	WD-40 repeat family protein, putative, expressed
LOC_Os09g13470	3.1	0.00753	1.7	0.3	up	plant protein of unknown function domain containing protein, expressed
LOC_Os06g48160	3.1	0.00199	1.7	0.1	up	glycosyl hydrolases family 16, putative, expressed
LOC_Os01g13560	3.1	0.00211	1.6	0.1	up	membrane associated DUF588 domain containing protein, putative, expressed
LOC_Os02g13360	3.1	0.00235	1.6	0.1	up	expressed protein
LOC_Os04g47640	3.1	0.00710	1.6	0.3	up	RWP-RK domain-containing protein, putative, expressed
LOC_Os01g41240	3.1	0.00230	1.6	0.1	up	hydrolase, alpha/beta fold family domain containing protein, expressed
LOC_Os08g02520	3.1	0.00123	1.6	0.1	up	OsSAUR31 - Auxin-responsive SAUR gene family member, expressed
LOC_Os05g51470	3.1	0.00060	1.6	0.0	up	2-aminoethanethiol dioxygenase, putative, expressed
LOC_Os02g51510	3.1	0.00755	1.6	0.3	up	expressed protein
LOC_Os08g44390	3.1	0.00387	1.6	0.2	up	EF hand family protein, putative, expressed
LOC_Os06g01200	3.1	0.00156	1.6	0.1	up	zinc finger, C3HC4 type domain containing protein, expressed
LOC_Os01g64790	3.1	0.00130	1.6	0.1	up	AP2 domain containing protein, expressed
LOC_Os03g56250	3.1	0.00145	1.6	0.1	up	LRR receptor-like protein kinase, putative, expressed
LOC_Os05g09550	3.1	0.00469	1.6	0.2	up	Der1-like family domain containing protein, expressed
LOC_Os03g41330	3.1	0.00358	1.6	0.2	up	DUF260 domain containing protein, putative, expressed
LOC_Os02g46460	3.1	0.00510	1.6	0.2	up	peptide transporter PTR2, putative, expressed
LOC_Os01g45659	3.1	0.00348	1.6	0.2	up	expressed protein
LOC_Os02g20540	3.1	0.00204	1.6	0.1	up	fasciclin domain containing protein, expressed
LOC_Os04g28410	3.1	0.00310	1.6	0.2	up	expressed protein
LOC_Os10g42610	3.0	0.00480	1.6	0.2	up	expressed protein
LOC_Os03g61960	3.0	0.00071	1.6	0.0	up	2Fe-2S iron-sulfur cluster binding domain containing protein, expressed
LOC_Os07g31540	3.0	0.00482	1.6	0.2	up	ubiquitin family protein, putative, expressed
LOC_Os08g29530	3.0	0.00064	1.6	0.0	up	double-stranded RNA binding motif containing protein, expressed
LOC_Os01g65520	3.0	0.00243	1.6	0.1	up	Sad1 / UNC-like C-terminal domain containing protein, putative, expressed
LOC_Os05g06970	3.0	0.00353	1.6	0.2	up	peroxidase precursor, putative, expressed
LOC_Os09g39730	3.0	0.00158	1.6	0.1	up	Core histone H2A/H2B/H3/H4 domain containing protein, putative, expressed
LOC_Os03g51390	3.0	0.00383	1.6	0.2	up	expressed protein

LOC_Os03g07880	3.0	0.00065	1.6	0.0	up	nuclear transcription factor Y subunit, putative, expressed
LOC_Os06g38294	3.0	0.00439	1.6	0.2	up	peptide transporter PTR2, putative, expressed
LOC_Os05g48040	3.0	0.00029	1.6	0.0	up	MATE efflux family protein, putative, expressed
LOC_Os12g43840	3.0	0.00309	1.6	0.2	up	ankyrin repeat domain-containing protein, putative, expressed
LOC_Os01g56180	3.0	0.00079	1.6	0.0	up	expressed protein
LOC_Os03g20090	3.0	0.00503	1.6	0.2	up	MYB family transcription factor, putative, expressed
LOC_Os02g56680	3.0	0.00138	1.6	0.1	up	dehydrogenase, putative, expressed
LOC_Os11g34750	3.0	0.00514	1.6	0.2	up	ABC1 family domain containing protein, putative, expressed
LOC_Os12g29400	3.0	0.00163	1.6	0.1	up	GRAM domain containing protein, expressed
LOC_Os03g22270	3.0	0.00195	1.6	0.1	up	auxin-repressed protein, putative, expressed
LOC_Os12g40470	3.0	0.00394	1.6	0.2	up	DC1 domain-containing protein, putative, expressed
LOC_Os07g29330	3.0	0.01446	1.6	0.4	up	serine/threonine-protein kinase CTR1, putative, expressed
LOC_Os04g49260	3.0	0.00178	1.6	0.1	up	heavy metal-associated domain containing protein, expressed
LOC_Os11g44810	3.0	0.00322	1.6	0.2	up	auxin-repressed protein, putative, expressed
LOC_Os07g47510	3.0	0.00863	1.6	0.3	up	stress-related protein, putative, expressed
LOC_Os04g31120	3.0	0.00225	1.6	0.1	up	OsFBK14 - F-box domain and kelch repeat containing protein, expressed
LOC_Os11g09160	3.0	0.00296	1.6	0.2	up	B3 DNA binding domain containing protein, expressed
LOC_Os05g30500	2.9	0.00181	1.6	0.1	up	expressed protein
LOC_Os08g44590	2.9	0.00334	1.6	0.2	up	gibberellin 20 oxidase 2, putative, expressed
LOC_Os08g42440	2.9	0.00067	1.6	0.0	up	CCT/B-box zinc finger protein, putative, expressed
LOC_Os01g68770	2.9	0.00194	1.5	0.1	up	selenium-binding protein, putative, expressed
LOC_Os01g57470	2.9	0.00384	1.5	0.2	up	EF hand family protein, putative, expressed
LOC_Os02g41840	2.9	0.00071	1.5	0.0	up	DUF584 domain containing protein, putative, expressed
LOC_Os03g55590	2.9	0.00292	1.5	0.2	up	MYB family transcription factor, putative, expressed
LOC_Os03g08000	2.9	0.00064	1.5	0.0	up	expressed protein
LOC_Os12g36110	2.9	0.00069	1.5	0.0	up	calmodulin binding protein, putative, expressed
LOC_Os03g22120	2.9	0.00173	1.5	0.1	up	sucrose synthase, putative, expressed
LOC_Os02g56460	2.9	0.00148	1.5	0.1	up	dehydrogenase, putative, expressed
LOC_Os12g39320	2.9	0.00074	1.5	0.0	up	DUF221 domain containing protein, expressed
LOC_Os02g39140	2.9	0.00356	1.5	0.2	up	helix-loop-helix DNA-binding domain containing protein, expressed
LOC_Os03g33090	2.9	0.00141	1.5	0.1	up	DUF260 domain containing protein, putative, expressed
LOC_Os02g37260	2.9	0.00130	1.5	0.1	up	hypothetical protein
LOC_Os10g41330	2.9	0.00311	1.5	0.2	up	AP2 domain containing protein, expressed
LOC_Os01g12280	2.9	0.00142	1.5	0.1	up	hAT dimerisation domain-containing protein, putative, expressed
LOC_Os10g39220	2.9	0.00209	1.5	0.1	up	tobamovirus multiplication protein, putative, expressed
LOC_Os01g03980	2.9	0.00220	1.5	0.1	up	expressed protein
LOC_Os01g03890	2.9	0.00178	1.5	0.1	up	DUF260 domain containing protein, putative, expressed
LOC_Os03g17310	2.9	0.00095	1.5	0.1	up	calcium-transporting ATPase, endoplasmic reticulum-type, putative, expressed
LOC_Os01g04330	2.9	0.00067	1.5	0.0	up	OsCML16 - Calmodulin-related calcium sensor protein, expressed
LOC_Os05g02390	2.9	0.00060	1.5	0.0	up	ZOS5-02 - C2H2 zinc finger protein, expressed
LOC_Os08g41720	2.9	0.00339	1.5	0.2	up	auxin efflux carrier component, putative, expressed
LOC_Os10g10130	2.9	0.00064	1.5	0.0	up	OsWAK112d - OsWAK receptor-like protein kinase, expressed
LOC_Os03g28400	2.8	0.00720	1.5	0.3	up	transport protein particle component, Bet3, domain containing protein, expressed
LOC_Os07g10620	2.8	0.00616	1.5	0.2	up	expressed protein
LOC_Os06g40360	2.8	0.00296	1.5	0.1	up	OsFBL30 - F-box domain and LRR containing protein, expressed
LOC_Os11g03820	2.8	0.00365	1.5	0.2	up	D-mannose binding lectin family protein, expressed
LOC_Os03g07830	2.8	0.00777	1.5	0.3	up	AP2 domain containing protein, expressed
LOC_Os05g40060	2.8	0.00703	1.5	0.3	up	WRKY48, expressed
LOC_Os05g33820	2.8	0.00030	1.5	0.0	up	lipase, putative, expressed
LOC_Os05g01310	2.8	0.00159	1.5	0.1	up	ankyrin repeat family protein, putative, expressed
LOC_Os06g12876	2.8	0.00623	1.5	0.2	up	expressed protein
LOC_Os11g34720	2.8	0.00161	1.5	0.1	up	Ser/Thr protein phosphatase family protein, putative, expressed
LOC_Os01g54890	2.8	0.00385	1.5	0.2	up	ethylene-responsive transcription factor 2, putative, expressed
LOC_Os12g36640	2.8	0.02140	1.5	0.5	up	universal stress protein domain containing protein, putative, expressed
LOC_Os06g46950	2.8	0.00697	1.5	0.3	up	EF hand family protein, putative, expressed
LOC_Os03g18130	2.8	0.01481	1.5	0.4	up	asparagine synthetase, putative, expressed
LOC_Os02g52010	2.8	0.00146	1.5	0.1	up	phosphate-induced protein 1 conserved region domain containing protein, expressed
LOC_Os05g39770	2.8	0.00552	1.5	0.2	up	aminotransferase, putative, expressed
LOC_Os07g06644	2.8	0.00083	1.5	0.0	up	expressed protein
LOC_Os03g06330	2.8	0.00088	1.5	0.1	up	tyrosine protein kinase domain containing protein, putative, expressed
LOC_Os03g44780	2.8	0.00337	1.5	0.2	up	elongation factor Tu family protein, putative, expressed
LOC_Os01g07300	2.8	0.00079	1.5	0.0	up	uncharacterized 50.6 kDa protein in the 5region of gyrA and gyrB, putative, expressed
LOC_Os12g04540	2.8	0.00679	1.5	0.2	up	expressed protein
LOC_Os01g11520	2.8	0.00252	1.5	0.1	up	RING-H2 finger protein, putative, expressed
LOC_Os10g32770	2.8	0.00154	1.5	0.1	up	WD repeat-containing protein, putative, expressed
LOC_Os02g32615	2.8	0.00451	1.5	0.2	up	expressed protein
LOC_Os03g29350	2.8	0.00889	1.5	0.3	up	von Willebrand factor type A domain containing protein, expressed
LOC_Os03g04370	2.8	0.00185	1.5	0.1	up	protein-methionine-S-oxide reductase, putative, expressed
LOC_Os06g45710	2.8	0.00101	1.5	0.1	up	phosphoglycerate kinase protein, putative, expressed
LOC_Os02g46690	2.8	0.00392	1.5	0.2	up	OsFBD8 - F-box and FBD domain containing protein, expressed
LOC_Os10g36260	2.8	0.00065	1.5	0.0	up	expressed protein
LOC_Os09g09920	2.8	0.00448	1.5	0.2	up	expressed protein
LOC_Os12g33165	2.8	0.00410	1.5	0.2	up	expressed protein
LOC_Os10g26940	2.8	0.00243	1.5	0.1	up	BURP domain containing protein, expressed
LOC_Os12g44070	2.8	0.00663	1.5	0.2	up	nodulin, putative, expressed
LOC_Os01g67820	2.8	0.00080	1.5	0.0	up	exo70 exocyst complex subunit domain containing protein, expressed
LOC_Os05g38270	2.8	0.00095	1.5	0.1	up	regulator of chromosome condensation, putative, expressed
LOC_Os04g32950	2.8	0.00430	1.5	0.2	up	calreticulin precursor protein, putative, expressed
LOC_Os02g37590	2.8	0.00391	1.5	0.2	up	glycerophosphoryl diester phosphodiesterase family protein, putative, expressed
LOC_Os03g08250	2.7	0.00067	1.5	0.0	up	expressed protein
LOC_Os02g52410	2.7	0.00151	1.5	0.1	up	expressed protein
LOC_Os01g57550	2.7	0.00590	1.5	0.2	up	nodulation protein-related, putative, expressed
LOC_Os10g03850	2.7	0.00067	1.5	0.0	up	OsFBX352 - F-box domain containing protein, expressed
LOC_Os05g41530	2.7	0.00179	1.5	0.1	up	ZOS5-11 - C2H2 zinc finger protein, expressed
LOC_Os02g41550	2.7	0.00211	1.4	0.1	up	FAD binding domain of DNA photolyase domain containing protein, expressed
LOC_Os07g44740	2.7	0.00128	1.4	0.1	up	chaperonin, putative, expressed
LOC_Os03g53020	2.7	0.00347	1.4	0.2	up	helix-loop-helix DNA-binding domain containing protein, expressed
LOC_Os06g47910	2.7	0.00812	1.4	0.3	up	GDSL-like lipase/acylhydrolase, putative, expressed
LOC_Os02g16040	2.7	0.00712	1.4	0.2	up	ubiquitin-conjugating enzyme, putative, expressed
LOC_Os10g30450	2.7	0.00250	1.4	0.1	up	heavy-metal-associated domain-containing protein, putative, expressed
LOC_Os03g51479	2.7	0.00199	1.4	0.1	up	maf, putative, expressed
LOC_Os03g47910	2.7	0.00294	1.4	0.1	up	expressed protein
LOC_Os03g18220	2.7	0.00353	1.4	0.2	up	pyruvate decarboxylase isozyme 2, putative, expressed
LOC_Os03g20949	2.7	0.00057	1.4	0.0	up	phospholipid-transporting ATPase, putative, expressed
LOC_Os07g38590	2.7	0.00269	1.4	0.1	up	carboxyl-terminal peptidase, putative, expressed
LOC_Os12g32580	2.7	0.00154	1.4	0.1	up	expressed protein
LOC_Os05g31040	2.7	0.00397	1.4	0.2	up	cytokinin dehydrogenase precursor, putative, expressed
LOC_Os12g02210	2.7	0.00358	1.4	0.2	up	RING finger protein, putative, expressed
LOC_Os10g21560	2.7	0.00383	1.4	0.2	up	no apical meristem protein, putative, expressed
LOC_Os01g07730	2.7	0.00234	1.4	0.1	up	phosphate/phosphoenolpyruvate translocator-related protein, putative, expressed
LOC_Os01g09620	2.7	0.01127	1.4	0.3	up	zinc finger/CCCH transcription factor, putative, expressed
LOC_Os01g56530	2.7	0.00170	1.4	0.1	up	DUF260 domain containing protein, putative, expressed
LOC_Os12g36640	2.7	0.00204	1.4	0.1	up	universal stress protein domain containing protein, putative, expressed
LOC_Os02g26700	2.7	0.00188	1.4	0.1	up	cation transport regulator-like protein 1, putative, expressed
LOC_Os02g34970	2.7	0.00235	1.4	0.1	up	no apical meristem protein, putative, expressed

LOC_Os02g58150	2.7	0.00351	1.4	0.2	up	expressed protein
LOC_Os08g38990	2.7	0.01956	1.4	0.4	up	WRKY30, expressed
LOC_Os05g41550	2.7	0.00480	1.4	0.2	up	expressed protein
LOC_Os03g39270	2.7	0.00206	1.4	0.1	up	expressed protein
LOC_Os10g32680	2.6	0.00288	1.4	0.1	up	expressed protein
LOC_Os03g13840	2.6	0.00128	1.4	0.1	up	senescence-associated protein, putative, expressed
LOC_Os01g51430	2.6	0.00207	1.4	0.1	up	green ripe-like, putative, expressed
LOC_Os01g61044	2.6	0.00230	1.4	0.1	up	transmembrane amino acid transporter protein, putative, expressed
LOC_Os03g55090	2.6	0.00259	1.4	0.1	up	alpha-glucan phosphorylast isozyme, putative, expressed
LOC_Os01g01610	2.6	0.00130	1.4	0.1	up	IscA-like iron-sulfur assembly protein, mitochondrial precursor, putative, expressed
LOC_Os09g27040	2.6	0.00973	1.4	0.3	up	GEX1, putative, expressed
LOC_Os02g28830	2.6	0.00390	1.4	0.2	up	tetratricopeptide repeat domain containing protein, expressed
LOC_Os04g40470	2.6	0.00456	1.4	0.2	up	cytochrome P450, putative, expressed
LOC_Os02g52140	2.6	0.00126	1.4	0.1	up	RNA recognition motif containing protein, putative, expressed
LOC_Os02g03280	2.6	0.00180	1.4	0.1	up	transmembrane BAX inhibitor motif-containing protein, putative, expressed
LOC_Os03g02550	2.6	0.00088	1.4	0.0	up	OsFBX76 - F-box domain containing protein, expressed
LOC_Os08g38086	2.6	0.00084	1.4	0.0	up	heat shock protein, putative, expressed
LOC_Os02g32610	2.6	0.00740	1.4	0.2	up	protein kinase domain containing protein, expressed
LOC_Os05g34325	2.6	0.00128	1.4	0.1	up	cytochrome P450 51, putative, expressed
LOC_Os08g16740	2.6	0.00128	1.4	0.1	up	expressed protein
LOC_Os04g48270	2.6	0.00531	1.4	0.2	up	OsFBX148 - F-box domain containing protein, expressed
LOC_Os04g51080	2.6	0.00109	1.4	0.1	up	scramblase, putative, expressed
LOC_Os01g08160	2.6	0.00181	1.4	0.1	up	MYB family transcription factor, putative, expressed
LOC_Os04g58190	2.6	0.00130	1.4	0.1	up	dof zinc finger domain containing protein, putative, expressed
LOC_Os04g31340	2.6	0.00103	1.4	0.1	up	CBS domain containing membrane protein, putative, expressed
LOC_Os01g33784	2.6	0.00107	1.4	0.1	up	lipase family protein, putative, expressed
LOC_Os09g11460	2.6	0.00186	1.4	0.1	up	AP2 domain containing protein, expressed
LOC_Os02g18390	2.6	0.00226	1.4	0.1	up	expressed protein
LOC_Os01g21590	2.6	0.00172	1.4	0.1	up	homeodomain, putative, expressed
LOC_Os01g39860	2.6	0.00157	1.4	0.1	up	1-aminocyclopropane-1-carboxylate oxidase protein, putative, expressed
LOC_Os12g38270	2.6	0.00234	1.4	0.1	up	metallothionein, putative, expressed
LOC_Os12g33150	2.6	0.00290	1.4	0.1	up	expressed protein
LOC_Os07g47530	2.6	0.00191	1.4	0.1	up	AAA-type ATPase family protein, putative, expressed
LOC_Os03g08270	2.6	0.00495	1.4	0.2	up	ataxin-2 C-terminal region family protein, expressed
LOC_Os10g22520	2.6	0.00104	1.4	0.1	up	cellulase, putative, expressed
LOC_Os03g51760	2.6	0.00227	1.4	0.1	up	OsFBX109 - F-box domain containing protein, expressed
LOC_Os11g04954	2.6	0.00383	1.4	0.2	up	DNA repair protein Rad51, putative, expressed
LOC_Os01g46350	2.6	0.00230	1.4	0.1	up	proteins of unknown function domain containing protein, expressed
LOC_Os03g42600	2.6	0.00233	1.4	0.1	up	expressed protein
LOC_Os09g11250	2.6	0.00842	1.4	0.3	up	co-chaperone GrpE protein, putative, expressed
LOC_Os09g33780	2.6	0.00230	1.4	0.1	up	expressed protein
LOC_Os11g08050	2.6	0.00349	1.4	0.1	up	expressed protein
LOC_Os01g60020	2.6	0.00353	1.4	0.1	up	NAC domain transcription factor, putative, expressed
LOC_Os02g54350	2.6	0.00572	1.4	0.2	up	hypothetical protein
LOC_Os04g55840	2.6	0.00725	1.4	0.2	up	expressed protein
LOC_Os01g50840	2.5	0.00677	1.3	0.2	up	expressed protein
LOC_Os03g57320	2.5	0.00560	1.3	0.2	up	expp1 protein precursor, putative, expressed
LOC_Os03g63970	2.5	0.00179	1.3	0.1	up	gibberellin 20 oxidase 1, putative, expressed
LOC_Os02g44500	2.5	0.00380	1.3	0.2	up	glutathione peroxidase, putative, expressed
LOC_Os05g10670	2.5	0.00320	1.3	0.1	up	zinc finger CCCH type family protein, putative, expressed
LOC_Os01g48950	2.5	0.00102	1.3	0.1	up	expressed protein
LOC_Os04g34970	2.5	0.00394	1.3	0.2	up	AP2 domain containing protein, expressed
LOC_Os01g16330	2.5	0.00173	1.3	0.1	up	OsRhmbd2 - Putative Rhomboid homologue, expressed
LOC_Os03g45740	2.5	0.00359	1.3	0.1	up	transferase family protein, putative, expressed
LOC_Os03g08624	2.5	0.00642	1.3	0.2	up	dihydroflavonol-4-reductase, putative, expressed
LOC_Os10g02920	2.5	0.00108	1.3	0.1	up	cytochrome b561, putative, expressed
LOC_Os02g55560	2.5	0.00101	1.3	0.1	up	protein phosphatase 2C, putative, expressed
LOC_Os03g51380	2.5	0.00405	1.3	0.2	up	expressed protein
LOC_Os02g44870	2.5	0.00086	1.3	0.0	up	dehydrin, putative, expressed
LOC_Os01g16350	2.5	0.00196	1.3	0.1	up	hydroxymethylglutaryl-CoA lyase, putative, expressed
LOC_Os12g36430	2.5	0.00250	1.3	0.1	up	expressed protein
LOC_Os03g20460	2.5	0.00102	1.3	0.1	up	guanylate kinase, putative, expressed
LOC_Os01g36820	2.5	0.02374	1.3	0.4	up	expressed protein
LOC_Os03g04840	2.5	0.04617	1.3	0.6	up	expressed protein
LOC_Os03g05900	2.5	0.00186	1.3	0.1	up	expressed protein
LOC_Os12g41930	2.5	0.00202	1.3	0.1	up	SRP40, C-terminal domain containing protein, expressed
LOC_Os10g28040	2.5	0.00243	1.3	0.1	up	histone acetyltransferase GCN5, putative, expressed
LOC_Os02g45160	2.5	0.00183	1.3	0.1	up	aluminum-activated malate transporter, putative, expressed
LOC_Os12g21734	2.5	0.00250	1.3	0.1	up	expressed protein
LOC_Os01g06660	2.5	0.00245	1.3	0.1	up	thiamine pyrophosphate enzyme, C-terminal TPP binding domain containing protein, expressed
LOC_Os01g54670	2.5	0.00213	1.3	0.1	up	coiled-coil domain-containing protein 25, putative, expressed
LOC_Os04g41920	2.5	0.00740	1.3	0.2	up	protein FAM133, putative, expressed
LOC_Os03g58530	2.5	0.00367	1.3	0.1	up	ES43 protein, putative, expressed
LOC_Os04g43820	2.5	0.00170	1.3	0.1	up	expressed protein
LOC_Os11g34270	2.5	0.00483	1.3	0.2	up	ubiquitin carboxyl-terminal hydrolase domain containing protein, expressed
LOC_Os07g43470	2.5	0.00111	1.3	0.1	up	GTP-binding protein, putative, expressed
LOC_Os01g50370	2.5	0.00192	1.3	0.1	up	STE_MEKK_ste11_MAP3K.4 - STE kinases include homologs to sterile 7, sterile 11 and sterile 20 from yeast, expressed
LOC_Os03g58160	2.5	0.00111	1.3	0.1	up	heat stress transcription factor, putative, expressed
LOC_Os06g03580	2.5	0.00116	1.3	0.1	up	zinc RING finger protein, putative, expressed
LOC_Os03g52940	2.5	0.00290	1.3	0.1	up	expressed protein
LOC_Os09g04370	2.5	0.00328	1.3	0.1	up	expressed protein
LOC_Os06g45060	2.5	0.00071	1.3	0.0	up	expressed protein
LOC_Os03g59070	2.5	0.00110	1.3	0.1	up	phosphatase, putative, expressed
LOC_Os05g04700	2.5	0.00400	1.3	0.2	up	OsRCI2-6 - Hydrophobic protein LTI6B, expressed
LOC_Os01g61180	2.5	0.00375	1.3	0.1	up	exo70 exocyst complex subunit domain containing protein, expressed
LOC_Os04g39110	2.5	0.00126	1.3	0.1	up	GASR4 - Gibberellin-regulated GASA/GAST/Snakin family protein precursor, expressed
LOC_Os08g33640	2.4	0.00290	1.3	0.1	up	expressed protein
LOC_Os02g33060	2.4	0.00767	1.3	0.2	up	expressed protein
LOC_Os09g28440	2.4	0.00359	1.3	0.1	up	AP2 domain containing protein, expressed
LOC_Os01g44050	2.4	0.00343	1.3	0.1	up	siroheme synthase, putative, expressed
LOC_Os03g49930	2.4	0.00621	1.3	0.2	up	pentatricopeptide, putative, expressed
LOC_Os11g10480	2.4	0.00102	1.3	0.1	up	dehydrogenase, putative, expressed
LOC_Os02g35160	2.4	0.00065	1.3	0.0	up	ligatin, putative, expressed
LOC_Os04g57739	2.4	0.00170	1.3	0.1	up	expressed protein
LOC_Os12g06550	2.4	0.00107	1.3	0.1	up	expressed protein
LOC_Os09g19930	2.4	0.00657	1.3	0.2	up	HOTHEAD precursor, putative, expressed
LOC_Os05g10754	2.4	0.00762	1.3	0.2	up	expressed protein
LOC_Os11g10470	2.4	0.00225	1.3	0.1	up	expressed protein
LOC_Os05g34050	2.4	0.00083	1.3	0.0	up	bZIP transcription factor domain containing protein, expressed
LOC_Os11g39370	2.4	0.00163	1.3	0.1	up	BRASSINOSTEROID INSENSITIVE 1-associated receptor kinase 1 precursor, putative, expressed
LOC_Os02g46473	2.4	0.00057	1.3	0.0	up	expressed protein
LOC_Os06g02440	2.4	0.00267	1.3	0.1	up	expressed protein
LOC_Os03g07190	2.4	0.00118	1.3	0.1	up	expressed protein
LOC_Os01g09430	2.4	0.00089	1.3	0.0	up	expressed protein
LOC_Os01g40760	2.4	0.00447	1.3	0.2	up	expressed protein

LOC_Os05g49420	2.4	0.00313	1.3	0.1	up	transcription factor, putative, expressed
LOC_Os03g53750	2.4	0.00413	1.3	0.2	up	nuclear prelamin A recognition factor, putative, expressed
LOC_Os10g37210	2.4	0.00173	1.3	0.1	up	FAD dependent oxidoreductase domain containing protein, expressed
LOC_Os12g04980	2.4	0.00223	1.3	0.1	up	DNA repair protein Rad51, putative, expressed
LOC_Os04g32740	2.4	0.00356	1.3	0.1	up	hydrolase, NUDIX family, domain containing protein, expressed
LOC_Os01g68160	2.4	0.00311	1.3	0.1	up	ZOS1-22 - C2H2 zinc finger protein, expressed
LOC_Os09g21230	2.4	0.00343	1.3	0.1	up	AMP-binding enzyme, putative, expressed
LOC_Os01g03480	2.4	0.00406	1.3	0.2	up	expressed protein
LOC_Os12g38490	2.4	0.00439	1.3	0.2	up	SCARECROW, putative, expressed
LOC_Os04g43440	2.4	0.00178	1.3	0.1	up	NB-ARC/LRR disease resistance protein, putative, expressed
LOC_Os01g52740	2.4	0.00718	1.3	0.2	up	expressed protein
LOC_Os11g03910	2.4	0.03081	1.3	0.5	up	zinc finger DHHC domain-containing protein, putative, expressed
LOC_Os06g03860	2.4	0.00470	1.2	0.2	up	uncharacterized membrane protein, putative, expressed
LOC_Os01g04660	2.4	0.01168	1.2	0.3	up	lipid phosphatase protein, putative, expressed
LOC_Os06g04020	2.4	0.00306	1.2	0.1	up	histone H1, putative, expressed
LOC_Os01g01660	2.4	0.00104	1.2	0.1	up	isoflavone reductase, putative, expressed
LOC_Os04g38970	2.4	0.00163	1.2	0.1	up	protein binding protein, putative, expressed
LOC_Os02g09690	2.4	0.00210	1.2	0.1	up	expressed protein
LOC_Os01g32770	2.4	0.00256	1.2	0.1	up	DUF260 domain containing protein, putative, expressed
LOC_Os04g56990	2.4	0.00282	1.2	0.1	up	Myb-like DNA-binding domain containing protein, putative, expressed
LOC_Os12g10750	2.4	0.00347	1.2	0.1	up	ARGOS, putative, expressed
LOC_Os01g63720	2.4	0.00436	1.2	0.2	up	hypothetical protein
LOC_Os02g16909	2.4	0.00856	1.2	0.2	up	dynein light chain type 1 domain containing protein, expressed
LOC_Os01g09280	2.4	0.00707	1.2	0.2	up	myb-related transcription activator, putative, expressed
LOC_Os07g48560	2.4	0.00088	1.2	0.0	up	homeobox domain containing protein, expressed
LOC_Os01g58890	2.4	0.00181	1.2	0.1	up	cysteine proteinase inhibitor precursor protein, putative, expressed
LOC_Os06g15560	2.4	0.00765	1.2	0.2	up	expressed protein
LOC_Os07g34710	2.4	0.00599	1.2	0.2	up	peroxidase precursor, putative, expressed
LOC_Os05g15530	2.4	0.00588	1.2	0.2	up	aminotransferase domain containing protein, putative, expressed
LOC_Os07g48040	2.4	0.02264	1.2	0.4	up	peroxidase precursor, putative, expressed
LOC_Os01g70710	2.4	0.00111	1.2	0.1	up	heavy metal-associated domain containing protein, expressed
LOC_Os01g46340	2.4	0.00554	1.2	0.2	up	chloroplast unusual positioning protein, putative, expressed
LOC_Os10g41820	2.4	0.00353	1.2	0.1	up	dynamin family protein, putative, expressed
LOC_Os06g49500	2.3	0.00454	1.2	0.2	up	integral membrane protein, putative, expressed
LOC_Os09g26490	2.3	0.00347	1.2	0.1	up	alpha/beta hydrolase fold, putative, expressed
LOC_Os01g05960	2.3	0.00173	1.2	0.1	up	receptor kinase, putative, expressed
LOC_Os07g35740	2.3	0.00219	1.2	0.1	up	TKL_IRAK_DUF26-ld.2 - DUF26 kinases have homology to DUF26 containing loci, expressed
LOC_Os09g39860	2.3	0.00060	1.2	0.0	up	protein kinase family protein, putative, expressed
LOC_Os02g55600	2.3	0.00173	1.2	0.1	up	expressed protein
LOC_Os09g30090	2.3	0.00515	1.2	0.2	up	WD domain, G-beta repeat domain containing protein, expressed
LOC_Os02g50710	2.3	0.00230	1.2	0.1	up	expressed protein
LOC_Os10g22520	2.3	0.00655	1.2	0.2	up	cellulase, putative, expressed
LOC_Os04g52550	2.3	0.00553	1.2	0.2	up	PAZ domain-containing protein, putative, expressed
LOC_Os01g43090	2.3	0.00372	1.2	0.1	up	oxidoreductase, aldo/keto reductase family protein, putative, expressed
LOC_Os06g03810	2.3	0.00433	1.2	0.2	up	expressed protein
LOC_Os05g45750	2.3	0.00445	1.2	0.2	up	ATP-dependent Clp protease ATP-binding subunit clpX, putative, expressed
LOC_Os02g52040	2.3	0.01095	1.2	0.3	up	phosphate-induced protein 1 conserved region domain containing protein, expressed
LOC_Os02g47390	2.3	0.00195	1.2	0.1	up	expressed protein
LOC_Os11g05494	2.3	0.00765	1.2	0.2	up	expressed protein
LOC_Os08g43390	2.3	0.00520	1.2	0.2	up	cytochrome P450, putative, expressed
LOC_Os01g39600	2.3	0.00195	1.2	0.1	up	expressed protein
LOC_Os09g23780	2.3	0.01421	1.2	0.3	up	expressed protein
LOC_Os11g09010	2.3	0.00660	1.2	0.2	up	lipase, putative, expressed
LOC_Os12g17804	2.3	0.00516	1.2	0.2	up	expressed protein
LOC_Os05g29050	2.3	0.00494	1.2	0.2	up	phospholipase D p1, putative, expressed
LOC_Os04g51920	2.3	0.00607	1.2	0.2	up	protein disulfide isomerase, putative, expressed
LOC_Os03g64219	2.3	0.00102	1.2	0.0	up	OTU-like cysteine protease family protein, putative, expressed
LOC_Os05g44100	2.3	0.00484	1.2	0.2	up	trehalose synthase, putative, expressed
LOC_Os01g45830	2.3	0.00098	1.2	0.0	up	sulfate transporter, putative, expressed
LOC_Os01g54550	2.3	0.00802	1.2	0.2	up	HSF-type DNA-binding domain containing protein, expressed
LOC_Os11g47310	2.3	0.01955	1.2	0.4	up	receptor kinase-like protein, identical, putative, expressed
LOC_Os03g58800	2.3	0.00065	1.2	0.0	up	ATPase, putative, expressed
LOC_Os01g68020	2.3	0.00235	1.2	0.1	up	BTBZ2 - Bric-a-Brac, Tramtrack, and Broad Complex BTB domain with TAZ zinc finger and Calmodulin-binding domains, expressed
LOC_Os07g37290	2.3	0.00174	1.2	0.1	up	expressed protein
LOC_Os03g52690	2.3	0.00280	1.2	0.1	up	CBS domain containing membrane protein, putative, expressed
LOC_Os08g07720	2.3	0.00074	1.2	0.0	up	transferase family protein, putative, expressed
LOC_Os08g10150	2.3	0.00261	1.2	0.1	up	SHR5-receptor-like kinase, putative, expressed
LOC_Os04g53998	2.3	0.00305	1.2	0.1	up	kinase, putative, expressed
LOC_Os01g04540	2.3	0.00169	1.2	0.1	up	serine/threonine-protein kinase At1g18390 precursor, putative, expressed
LOC_Os11g40249	2.3	0.00413	1.2	0.1	up	expressed protein
LOC_Os04g45730	2.3	0.00150	1.2	0.1	up	protein kinase domain containing protein, expressed
LOC_Os06g14670	2.3	0.00459	1.2	0.2	up	ODORANT1, putative, expressed
LOC_Os06g21550	2.3	0.00101	1.2	0.0	up	thioredoxin domain-containing protein 17, putative, expressed
LOC_Os03g19850	2.3	0.01634	1.2	0.3	up	expressed protein
LOC_Os04g54830	2.3	0.00194	1.2	0.1	up	expressed protein
LOC_Os11g47580	2.3	0.02151	1.2	0.4	up	glycosyl hydrolase, putative, expressed
LOC_Os01g52730	2.3	0.00168	1.2	0.1	up	DUF584 domain containing protein, putative, expressed
LOC_Os11g29630	2.3	0.00409	1.2	0.1	up	expressed protein
LOC_Os03g47280	2.3	0.00754	1.2	0.2	up	VQ domain containing protein, putative, expressed
LOC_Os05g50470	2.3	0.00777	1.2	0.2	up	expressed protein
LOC_Os10g30850	2.3	0.00351	1.2	0.1	up	zinc finger, RING-type, putative, expressed
LOC_Os03g19260	2.3	0.00280	1.2	0.1	up	expressed protein
LOC_Os08g13000	2.3	0.01050	1.2	0.2	up	MBTB20 - Bric-a-Brac, Tramtrack, Broad Complex BTB domain with Meprin and TRAF Homology MATH domain, expressed
LOC_Os02g32520	2.3	0.00349	1.2	0.1	up	ERD1 protein, chloroplast precursor, putative, expressed
LOC_Os04g30250	2.3	0.00150	1.2	0.1	up	wall-associated receptor kinase-like 5 precursor, putative, expressed
LOC_Os01g56480	2.3	0.00543	1.2	0.2	up	expressed protein
LOC_Os02g10440	2.3	0.00568	1.2	0.2	up	protein with a conserved N-terminal region, putative, expressed
LOC_Os03g28080	2.3	0.01090	1.2	0.3	up	ring-H2 zinc finger protein, putative, expressed
LOC_Os02g33720	2.3	0.00128	1.2	0.1	up	RING-H2 finger protein, putative, expressed
LOC_Os05g44900	2.3	0.00222	1.2	0.1	up	expressed protein
LOC_Os03g63150	2.3	0.00283	1.2	0.1	up	powdery mildew resistance protein PM3b, putative, expressed
LOC_Os08g36030	2.3	0.00513	1.2	0.2	up	plant viral response family protein, putative, expressed
LOC_Os08g34190	2.3	0.00235	1.2	0.1	up	stromal cell-derived factor 2-like protein precursor, putative, expressed
LOC_Os11g34700	2.3	0.01050	1.2	0.2	up	ZOS11-04 - C2H2 zinc finger protein, expressed
LOC_Os05g23320	2.3	0.00182	1.2	0.1	up	expressed protein
LOC_Os02g52490	2.2	0.00239	1.2	0.1	up	expressed protein
LOC_Os01g64020	2.2	0.00369	1.2	0.1	up	transcription factor, putative, expressed
LOC_Os06g12370	2.2	0.00192	1.2	0.1	up	OsFtsH6 FtsH protease, homologue of AtFtsH6, expressed
LOC_Os08g33150	2.2	0.00138	1.2	0.1	up	MYB family transcription factor, putative, expressed
LOC_Os02g56820	2.2	0.01202	1.2	0.3	up	OsFBX69 - F-box domain containing protein, expressed
LOC_Os01g59910	2.2	0.03576	1.2	0.5	up	OsFBX29 - F-box domain containing protein, expressed
LOC_Os02g33770	2.2	0.00147	1.2	0.1	up	homeodomain, putative, expressed
LOC_Os03g39230	2.2	0.00340	1.2	0.1	up	OTU-like cysteine protease family protein, putative, expressed
LOC_Os03g22170	2.2	0.00079	1.2	0.0	up	AP2 domain containing protein, expressed
LOC_Os03g03410	2.2	0.00230	1.2	0.1	up	serine/threonine-protein kinase, putative, expressed

LOC_Os04g28805	2.2	0.00234	1.2	0.1	up	expressed protein
LOC_Os03g08830	2.2	0.00116	1.2	0.1	up	WD domain, G-beta repeat domain containing protein, expressed
LOC_Os05g39310	2.2	0.00362	1.2	0.1	up	thiamine pyrophosphate enzyme, C-terminal TPP binding domain containing protein, expressed
LOC_Os01g55240	2.2	0.00356	1.2	0.1	up	gibberellin 2-beta-dioxygenase, putative, expressed
LOC_Os03g04050	2.2	0.00634	1.2	0.2	up	protein kinase family protein, putative, expressed
LOC_Os02g09830	2.2	0.02242	1.2	0.4	up	bZIP transcription factor domain containing protein, expressed
LOC_Os02g55840	2.2	0.00145	1.2	0.1	up	expressed protein
LOC_Os03g61670	2.2	0.00165	1.2	0.1	up	calreticulin precursor, putative, expressed
LOC_Os06g12090	2.2	0.00380	1.2	0.1	up	miro, putative, expressed
LOC_Os06g22960	2.2	0.00293	1.2	0.1	up	aquaporin protein, putative, expressed
LOC_Os08g19670	2.2	0.00200	1.2	0.1	up	expressed protein
LOC_Os01g04800	2.2	0.00194	1.2	0.1	up	B3 DNA binding domain containing protein, expressed
LOC_Os10g40420	2.2	0.00092	1.2	0.0	up	LTPL138 - Protease inhibitor/seed storage/LTP family protein precursor, expressed
LOC_Os11g40070	2.2	0.00126	1.2	0.1	up	expressed protein
LOC_Os07g46555	2.2	0.00660	1.1	0.2	up	F-box domain containing protein, expressed
LOC_Os01g34790	2.2	0.00259	1.1	0.1	up	expressed protein
LOC_Os01g10110	2.2	0.00732	1.1	0.2	up	cytokinin dehydrogenase precursor, putative, expressed
LOC_Os03g06400	2.2	0.00767	1.1	0.2	up	expressed protein
LOC_Os11g38790	2.2	0.02437	1.1	0.4	up	expressed protein
LOC_Os03g12879	2.2	0.00509	1.1	0.2	up	expressed protein
LOC_Os05g01140	2.2	0.00571	1.1	0.2	up	methyltransferase, putative, expressed
LOC_Os04g35560	2.2	0.00057	1.1	0.0	up	glutathione S-transferase, putative, expressed
LOC_Os05g04150	2.2	0.00356	1.1	0.1	up	expressed protein
LOC_Os01g09700	2.2	0.00178	1.1	0.1	up	aminotransferase, classes I and II, domain containing protein, expressed
LOC_Os10g03760	2.2	0.00583	1.1	0.2	up	OsFBX350 - F-box domain containing protein, expressed
LOC_Os02g42250	2.2	0.00465	1.1	0.2	up	5-azacytidine resistance protein related, putative, expressed
LOC_Os12g38051	2.2	0.00096	1.1	0.0	up	metallothionein, putative, expressed
LOC_Os02g42350	2.2	0.00102	1.1	0.0	up	nitrilase, putative, expressed
LOC_Os04g42720	2.2	0.00872	1.1	0.2	up	TMS membrane protein/tumour differentially expressed protein, putative, expressed
LOC_Os03g55630	2.2	0.00481	1.1	0.2	up	expressed protein
LOC_Os07g17560	2.2	0.00363	1.1	0.1	up	expressed protein
LOC_Os06g38430	2.2	0.00507	1.1	0.2	up	expressed protein
LOC_Os03g17670	2.2	0.00149	1.1	0.1	up	expressed protein
LOC_Os05g39690	2.2	0.00113	1.1	0.1	up	oxidoreductase, aldo/keto reductase family protein, putative, expressed
LOC_Os08g40919	2.2	0.01013	1.1	0.2	up	expressed protein
LOC_Os05g50470	2.2	0.00139	1.1	0.1	up	expressed protein
LOC_Os02g13640	2.2	0.00227	1.1	0.1	up	protein phosphatase 1 regulatory subunit SDS22, putative, expressed
LOC_Os07g38740	2.2	0.01735	1.1	0.3	up	expressed protein
LOC_Os11g32380	2.2	0.00261	1.1	0.1	up	expressed protein
LOC_Os06g24190	2.2	0.00102	1.1	0.0	up	expressed protein
LOC_Os12g42620	2.2	0.01432	1.1	0.3	up	hypothetical protein
LOC_Os04g40020	2.2	0.00867	1.1	0.2	up	expressed protein
LOC_Os11g32780	2.2	0.00196	1.1	0.1	up	expressed protein
LOC_Os02g07690	2.2	0.00155	1.1	0.1	up	VQ domain containing protein, putative, expressed
LOC_Os08g11760	2.2	0.04431	1.1	0.5	up	expressed protein
LOC_Os01g15010	2.2	0.00160	1.1	0.1	up	miro, putative, expressed
LOC_Os01g57420	2.2	0.00064	1.1	0.0	up	diacylglycerol kinase, putative, expressed
LOC_Os03g22390	2.2	0.00328	1.1	0.1	up	universal stress protein domain containing protein, putative, expressed
LOC_Os03g21480	2.2	0.00138	1.1	0.1	up	HAD superfamily phosphatase, putative, expressed
LOC_Os07g07480	2.2	0.00093	1.1	0.0	up	HNH endonuclease domain-containing protein, putative, expressed
LOC_Os03g06410	2.2	0.00446	1.1	0.1	up	protein kinase domain containing protein, expressed
LOC_Os09g36770	2.2	0.00552	1.1	0.2	up	NTMC2Type1.2 protein, putative, expressed
LOC_Os01g09800	2.2	0.00151	1.1	0.1	up	BTBA1 - Bric-a-Brac,Tramtrack, Broad Complex BTB domain with Ankyrin repeat region, expressed
LOC_Os07g47510	2.2	0.00333	1.1	0.1	up	stress-related protein, putative, expressed
LOC_Os11g05070	2.2	0.00238	1.1	0.1	up	sodium/calcium exchanger protein, putative, expressed
LOC_Os08g44015	2.2	0.00361	1.1	0.1	up	growth regulator related protein, putative, expressed
LOC_Os08g37950	2.2	0.00073	1.1	0.0	up	copper-transporting ATPase PAA1, putative, expressed
LOC_Os01g67330	2.2	0.00145	1.1	0.1	up	nucleotide-sugar transporter family protein, putative, expressed
LOC_Os05g35650	2.2	0.01458	1.1	0.3	up	peptide transporter PTR2, putative, expressed
LOC_Os02g02780	2.2	0.00761	1.1	0.2	up	protein kinase family protein, putative, expressed
LOC_Os01g39850	2.2	0.00573	1.1	0.2	up	histone-like transcription factor and archaeal histone, putative, expressed
LOC_Os09g37500	2.2	0.00355	1.1	0.1	up	OsSAUR55 - Auxin-responsive SAUR gene family member, expressed
LOC_Os02g02524	2.2	0.00113	1.1	0.0	up	canopy homolog 2 precursor, putative, expressed
LOC_Os02g49770	2.2	0.00083	1.1	0.0	up	NHL repeat-containing protein, putative, expressed
LOC_Os06g23740	2.2	0.03829	1.1	0.5	up	expressed protein
LOC_Os06g43960	2.2	0.00278	1.1	0.1	up	expressed protein
LOC_Os12g40770	2.2	0.00202	1.1	0.1	up	ankyrin-1, putative, expressed
LOC_Os05g06410	2.2	0.00446	1.1	0.1	up	expressed protein
LOC_Os11g07230	2.2	0.00440	1.1	0.1	up	receptor kinase, putative, expressed
LOC_Os05g37450	2.2	0.00887	1.1	0.2	up	starch binding domain containing protein, putative, expressed
LOC_Os07g47350	2.2	0.03717	1.1	0.4	up	potassium transporter, putative, expressed
LOC_Os03g15270	2.2	0.00172	1.1	0.1	up	gibberellin receptor GID1L2, putative, expressed
LOC_Os04g42910	2.2	0.00234	1.1	0.1	up	expressed protein
LOC_Os04g57810	2.2	0.00040	1.1	0.0	up	GA18008-PA, putative, expressed
LOC_Os05g34710	2.2	0.00575	1.1	0.2	up	expressed protein
LOC_Os05g33080	2.2	0.00271	1.1	0.1	up	serine/threonine-protein kinase, putative, expressed
LOC_Os04g32580	2.2	0.00126	1.1	0.1	up	expressed protein
LOC_Os01g63410	2.1	0.00535	1.1	0.2	up	expressed protein
LOC_Os10g20470	2.1	0.00114	1.1	0.0	up	MATE efflux family protein, putative, expressed
LOC_Os09g32330	2.1	0.00668	1.1	0.2	up	expressed protein
LOC_Os03g17060	2.1	0.00314	1.1	0.1	up	RNA recognition motif containing protein, putative, expressed
LOC_Os06g35500	2.1	0.00311	1.1	0.1	up	expressed protein
LOC_Os02g45780	2.1	0.00513	1.1	0.2	up	zinc finger, C3HC4 type domain containing protein, expressed
LOC_Os02g02920	2.1	0.00194	1.1	0.1	up	HVA22, putative, expressed
LOC_Os10g39090	2.1	0.00894	1.1	0.2	up	hydrolase, alpha/beta fold family protein, putative, expressed
LOC_Os03g25050	2.1	0.00211	1.1	0.1	up	chaperonin, putative, expressed
LOC_Os07g48820	2.1	0.00071	1.1	0.0	up	transcription factor, putative, expressed
LOC_Os09g27050	2.1	0.00573	1.1	0.2	up	HD domain containing protein, putative, expressed
LOC_Os05g35110	2.1	0.00806	1.1	0.2	up	OsFBL22 - F-box domain and LRR containing protein, expressed
LOC_Os04g48830	2.1	0.00814	1.1	0.2	up	DUF623 domain containing protein, expressed
LOC_Os01g35230	2.1	0.00786	1.1	0.2	up	1-aminocyclopropane-1-carboxylate oxidase homolog 1, putative, expressed
LOC_Os02g46910	2.1	0.01479	1.1	0.3	up	glycosyl hydrolases family 16, putative, expressed
LOC_Os09g27010	2.1	0.00057	1.1	0.0	up	tyrosine protein kinase domain containing protein, putative, expressed
LOC_Os03g58250	2.1	0.00161	1.1	0.1	up	bZIP transcription factor domain containing protein, expressed
LOC_Os01g14090	2.1	0.00455	1.1	0.1	up	kinesin motor domain containing protein, putative, expressed
LOC_Os05g38000	2.1	0.00670	1.1	0.2	up	ATROPGEF7/ROPGEF7, putative, expressed
LOC_Os12g39710	2.1	0.00227	1.1	0.1	up	expressed protein
LOC_Os11g08950	2.1	0.00102	1.1	0.0	up	protein kinase family protein, putative, expressed
LOC_Os12g06020	2.1	0.00417	1.1	0.1	up	mRNA-decapping enzyme, putative, expressed
LOC_Os12g32610	2.1	0.01091	1.1	0.2	up	expressed protein
LOC_Os09g27260	2.1	0.01699	1.1	0.3	up	plant viral response family protein, putative, expressed
LOC_Os01g64520	2.1	0.00185	1.1	0.1	up	uricase, putative, expressed
LOC_Os03g57120	2.1	0.00345	1.1	0.1	up	ferredoxin--NADP reductase, chloroplast precursor, putative, expressed
LOC_Os02g09060	2.1	0.02794	1.1	0.4	up	BRCA1-associated protein, putative, expressed
LOC_Os05g12481	2.1	0.00334	1.1	0.1	up	expressed protein

LOC_Os07g42510	2.1	0.00570	1.1	0.2	up	AP2 domain containing protein, expressed
LOC_Os11g08380	2.1	0.00728	1.1	0.2	up	1-aminocyclopropane-1-carboxylate oxidase, putative, expressed
LOC_Os01g47280	2.1	0.01098	1.1	0.2	up	expressed protein
LOC_Os01g48446	2.1	0.00147	1.1	0.1	up	no apical meristem protein, putative, expressed
LOC_Os05g19970	2.1	0.00211	1.1	0.1	up	ZOS5-06 - C2H2 zinc finger protein, expressed
LOC_Os04g47190	2.1	0.00181	1.1	0.1	up	aminotransferase domain containing protein, putative, expressed
LOC_Os08g43210	2.1	0.00878	1.1	0.2	up	AP2 domain containing protein, expressed
LOC_Os01g64250	2.1	0.00138	1.1	0.1	up	hemerythrin family protein, expressed
LOC_Os02g48110	2.1	0.00169	1.1	0.1	up	DnaK family protein, putative, expressed
LOC_Os08g02690	2.1	0.01377	1.1	0.3	up	MA3 domain containing protein, expressed
LOC_Os07g07550	2.1	0.00598	1.1	0.2	up	galactose-1-phosphate uridyl transferase, putative, expressed
LOC_Os04g51880	2.1	0.00261	1.1	0.1	up	GHMP kinases ATP-binding protein, putative, expressed
LOC_Os07g20340	2.1	0.00645	1.1	0.2	up	heavy metal-associated domain containing protein, expressed
LOC_Os02g10830	2.1	0.00150	1.1	0.1	up	serine acetyltransferase protein, putative, expressed
LOC_Os03g19375	2.1	0.00114	1.1	0.0	up	expressed protein
LOC_Os04g31270	2.1	0.00255	1.1	0.1	up	EMB1688, putative, expressed
LOC_Os10g08026	2.1	0.00176	1.1	0.1	up	lecithin:cholesterol acyltransferase, putative, expressed
LOC_Os03g60850	2.1	0.03272	1.1	0.4	up	peptide transporter PTR2, putative, expressed
LOC_Os08g40940	2.1	0.01470	1.1	0.3	up	expressed protein
LOC_Os05g45320	2.1	0.00194	1.1	0.1	up	ubiquitin domain-containing protein 1, putative, expressed
LOC_Os04g51770	2.1	0.00154	1.1	0.1	up	expressed protein
LOC_Os01g05064	2.1	0.00186	1.1	0.1	up	expressed protein
LOC_Os09g28489	2.1	0.01053	1.1	0.2	up	expressed protein
LOC_Os02g35530	2.1	0.01095	1.1	0.2	up	OsFBK8 - F-box domain and kelch repeat containing protein, expressed
LOC_Os03g51690	2.1	0.02472	1.1	0.3	up	Homeobox domain containing protein, expressed
LOC_Os01g52410	2.1	0.00128	1.1	0.1	up	myb-like DNA-binding domain containing protein, putative, expressed
LOC_Os07g05510	2.1	0.02248	1.1	0.3	up	expressed protein
LOC_Os06g42850	2.1	0.00123	1.1	0.1	up	extracellular ligand-gated ion channel, putative, expressed
LOC_Os06g48300	2.1	0.01319	1.1	0.3	up	protein phosphatase 2C, putative, expressed
LOC_Os05g41120	2.1	0.00137	1.1	0.1	up	endoplasmic reticulum-Golgi intermediate compartment protein 3, putative, expressed
LOC_Os05g51420	2.1	0.02846	1.1	0.4	up	hypersensitive-induced response protein, putative, expressed
LOC_Os10g34450	2.1	0.00830	1.1	0.2	up	expressed protein
LOC_Os04g58810	2.1	0.01792	1.1	0.3	up	CAF1 family ribonuclease containing protein, putative, expressed
LOC_Os02g33020	2.1	0.00709	1.1	0.2	up	SOUL heme-binding protein, putative, expressed
LOC_Os01g40580	2.1	0.00506	1.1	0.1	up	hypersensitive-induced response protein, putative, expressed
LOC_Os01g43550	2.1	0.00488	1.1	0.1	up	WRKY12, expressed
LOC_Os12g17570	2.1	0.00667	1.1	0.2	up	GDSL-like lipase/acylhydrolase, putative, expressed
LOC_Os02g26160	2.1	0.00962	1.1	0.2	up	receptor lectin kinase like protein, putative, expressed
LOC_Os03g61540	2.1	0.00732	1.1	0.2	up	lipase class 3 family protein, putative, expressed
LOC_Os01g17010	2.1	0.00483	1.1	0.1	up	phospholipid-transporting ATPase, putative, expressed
LOC_Os09g27410	2.1	0.00101	1.1	0.0	up	expressed protein
LOC_Os07g10420	2.1	0.00121	1.1	0.0	up	expressed protein
LOC_Os01g53840	2.1	0.00217	1.0	0.1	up	protein kinase family protein, putative, expressed
LOC_Os12g10880	2.1	0.00635	1.0	0.2	up	expressed protein
LOC_Os09g02540	2.1	0.01278	1.0	0.2	up	expressed protein
LOC_Os05g39320	2.1	0.00353	1.0	0.1	up	thiamine pyrophosphate enzyme, C-terminal TPP binding domain containing protein, expressed
LOC_Os02g03410	2.1	0.00128	1.0	0.1	up	CAMK_CAMK_like.12 - CAMK includes calcium/calmodulin depedent protein kinases, expressed
LOC_Os10g37830	2.1	0.00211	1.0	0.1	up	OsFBX391 - F-box domain containing protein, expressed
LOC_Os09g25570	2.1	0.01373	1.0	0.3	up	aspartic proteinase, putative, expressed
LOC_Os11g39190	2.1	0.00643	1.0	0.2	up	NB-ARC domain containing protein, putative, expressed
LOC_Os02g10210	2.1	0.00590	1.0	0.2	up	expressed protein
LOC_Os09g29510	2.1	0.00538	1.0	0.2	up	OsWAK80 - OsWAK receptor-like protein kinase, expressed
LOC_Os07g14310	2.1	0.00397	1.0	0.1	up	expressed protein
LOC_Os07g26210	2.1	0.01573	1.0	0.3	up	non-lysosomal glucosylceramidase, putative, expressed
LOC_Os04g52500	2.1	0.00620	1.0	0.2	up	lecithine cholesterol acyltransferase, putative, expressed
LOC_Os01g39020	2.1	0.01012	1.0	0.2	up	HSF-type DNA-binding domain containing protein, expressed
LOC_Os07g07760	2.1	0.00722	1.0	0.2	up	expressed protein
LOC_Os08g08700	2.1	0.00249	1.0	0.1	up	ubiquitin family protein, putative, expressed
LOC_Os12g13340	2.1	0.00375	1.0	0.1	up	expressed protein
LOC_Os02g45380	2.1	0.00153	1.0	0.1	up	MATE domain containing protein, expressed
LOC_Os02g27590	2.1	0.01323	1.0	0.2	up	hypothetical protein
LOC_Os08g10310	2.1	0.00105	1.0	0.0	up	SHR5-receptor-like kinase, putative, expressed
LOC_Os12g37410	2.1	0.01801	1.0	0.3	up	CPuORF5 - conserved peptide uORF-containing transcript, expressed
LOC_Os02g33710	2.0	0.00877	1.0	0.2	up	decarboxylase, putative, expressed
LOC_Os01g67590	2.0	0.00795	1.0	0.2	up	oligopeptidase, putative, expressed
LOC_Os08g42750	2.0	0.00446	1.0	0.1	up	CAMK_CAMK_like.37 - CAMK includes calcium/calmodulin depedent protein kinases, expressed
LOC_Os08g39370	2.0	0.00692	1.0	0.2	up	citrate transporter, putative, expressed
LOC_Os03g03200	2.0	0.00271	1.0	0.1	up	hydrolase, alpha/beta fold family protein, putative, expressed
LOC_Os03g04530	2.0	0.00458	1.0	0.1	up	cytochrome P450, putative, expressed
LOC_Os11g14520	2.0	0.00718	1.0	0.2	up	expressed protein
LOC_Os04g41960	2.0	0.00392	1.0	0.1	up	NADP-dependent oxidoreductase, putative, expressed
LOC_Os01g65900	2.0	0.00067	1.0	0.0	up	chitin-inducible gibberellin-responsive protein, putative, expressed
LOC_Os09g15860	2.0	0.00145	1.0	0.1	up	hypothetical protein
LOC_Os07g08880	2.0	0.00102	1.0	0.0	up	ES43 protein, putative, expressed
LOC_Os11g11090	2.0	0.00864	1.0	0.2	up	expressed protein
LOC_Os07g18180	2.0	0.00110	1.0	0.0	up	expressed protein
LOC_Os01g63250	2.0	0.00225	1.0	0.1	up	josephin, putative, expressed
LOC_Os10g04020	2.0	0.00348	1.0	0.1	up	OsFBX356 - F-box domain containing protein, expressed
LOC_Os07g28820	2.0	0.00411	1.0	0.1	up	ribonuclease 2-5A family protein, expressed
LOC_Os07g05370	2.0	0.00120	1.0	0.0	up	TKL_IRAK_CrRLK1L-1.15 - The CrRLK1L-1 subfamily has homology to the CrRLK1L homolog, expressed
LOC_Os06g01360	2.0	0.00289	1.0	0.1	up	homogentisate 1,2-dioxygenase, putative, expressed
LOC_Os01g33400	2.0	0.01291	1.0	0.2	up	helix-loop-helix DNA-binding domain containing protein, expressed
LOC_Os03g06490	2.0	0.00185	1.0	0.1	up	expressed protein
LOC_Os07g37620	2.0	0.00413	1.0	0.1	up	fiber expressed protein, putative, expressed
LOC_Os09g31990	2.0	0.00586	1.0	0.2	up	expressed protein
LOC_Os06g19444	2.0	0.00430	1.0	0.1	up	CCT/B-box zinc finger protein, putative, expressed
LOC_Os07g40710	2.0	0.00724	1.0	0.2	up	circadian clock coupling factor-related, putative, expressed
LOC_Os09g26730	2.0	0.00312	1.0	0.1	up	chaperonin, putative, expressed
LOC_Os03g18490	2.0	0.00878	1.0	0.2	up	RPGR, putative, expressed
LOC_Os05g36290	2.0	0.00561	1.0	0.2	up	actin, putative, expressed
LOC_Os01g26130	2.0	0.00114	1.0	0.0	up	expressed protein
LOC_Os03g16350	2.0	0.00199	1.0	0.1	up	DNA binding protein, putative, expressed
LOC_Os10g03690	2.0	0.00124	1.0	0.0	up	OsFBX346 - F-box domain containing protein, expressed
LOC_Os11g13934	2.0	0.02608	1.0	0.3	up	expressed protein
LOC_Os11g10510	2.0	0.00814	1.0	0.2	up	dehydrogenase, putative, expressed
LOC_Os06g03690	2.0	0.00899	1.0	0.2	up	RNA recognition motif containing protein, putative, expressed
LOC_Os04g01960	2.0	0.00440	1.0	0.1	up	expressed protein
LOC_Os07g47350	2.0	0.01880	1.0	0.3	up	potassium transporter, putative, expressed
LOC_Os05g05940	2.0	0.00150	1.0	0.1	up	stress-related protein, putative, expressed
LOC_Os03g38540	2.0	0.00441	1.0	0.1	up	folic acid binding protein, putative, expressed
LOC_Os10g31780	2.0	0.00145	1.0	0.1	up	oxidoreductase, short chain dehydrogenase/reductase family domain containing protein, expressed
LOC_Os03g40260	2.0	0.00234	1.0	0.1	up	Regulator of chromosome condensation domain containing protein, expressed
LOC_Os02g55190	2.0	0.00470	1.0	0.1	up	expressed protein
LOC_Os07g40300	2.0	0.01324	1.0	0.2	up	ZOS7-10 - C2H2 zinc finger protein, expressed
LOC_Os08g34740	2.0	0.00265	1.0	0.1	up	SGT1 protein, putative, expressed

LOC_Os07g48510	2.0	0.00206	1.0	0.1	up	thioredoxin, putative, expressed
LOC_Os11g42800	2.0	0.00412	1.0	0.1	up	kinesin motor domain containing protein, putative, expressed
LOC_Os03g26180	2.0	0.00117	1.0	0.0	up	expressed protein
LOC_Os02g32110	2.0	0.00343	1.0	0.1	up	exostosin family domain containing protein, expressed
LOC_Os05g37140	2.0	0.00119	1.0	0.0	up	2Fe-2S iron-sulfur cluster binding domain containing protein, expressed
LOC_Os11g10770	2.0	0.00174	1.0	0.1	up	disease resistance protein RGA3, putative, expressed
LOC_Os01g12660	2.0	0.01015	1.0	0.2	up	AAA-type ATPase family protein, putative, expressed
LOC_Os01g03060	2.0	0.00440	1.0	0.1	up	splicing factor, putative, expressed
LOC_Os07g41200	2.0	0.00136	1.0	0.1	up	expressed protein
LOC_Os01g70590	2.0	0.00163	1.0	0.1	up	DUF567 domain containing protein, putative, expressed
LOC_Os01g58700	2.0	0.00128	1.0	0.1	up	expressed protein
LOC_Os05g07070	2.0	0.00138	1.0	0.1	up	zinc finger, C3HC4 type domain containing protein, expressed
LOC_Os06g45090	2.0	0.00181	1.0	0.1	up	expressed protein
LOC_Os03g14140	2.0	0.00284	1.0	0.1	up	POEI16 - Pollen Ole e I allergen and extensin family protein precursor, expressed
LOC_Os05g32474	2.0	0.00306	1.0	0.1	up	expressed protein
LOC_Os04g28710	2.0	0.00575	1.0	0.2	up	hypothetical protein
LOC_Os04g44354	2.0	0.00942	1.0	0.2	up	UDP-glucoronosyl and UDP-glucosyl transferase domain containing protein, expressed
LOC_Os05g34010	2.0	0.00185	1.0	0.1	up	peptide transporter PTR2, putative, expressed
LOC_Os01g09120	2.0	0.00159	1.0	0.1	up	Twin LOV 1, putative, expressed
LOC_Os08g29160	2.0	0.00170	1.0	0.1	up	5-AMP-activated protein kinase beta-1 subunit-related, putative, expressed
