# Supplementary material for: The Arabidopsis transcriptional regulator DPB3‐1 enhances heat stress tolerance without growth retardation in rice
Source: Plant Biotechnol J. 2016 Feb 3;14(8):1756–67. doi: 10.1111/pbi.12535 (PMC5067654; doi:10.1111/pbi.12535)
Supplement: Supplementary file 5 — Table S5 Down‐regulated gene in DPB3‐1‐overexpressing rice under the nonstress condition. [file PBI-14-1756-s001.docx]

**Table S5** Downregulated gene in *DPB3-1*-overexpressing rice under the non-stress condition.

| MSU7_locus | Fold Change | Q-Value | Average (log2) | SD (log2) | Regulation | Description^a)^ |
| --- | --- | --- | --- | --- | --- | --- |
| LOC_Os05g08910 | 3.8 | 0.04004 | -1.9 | 0.1 | down | expressed protein |

^a)^ Description as given by the MSU 7.0 database.
