# Supplementary material for: The Arabidopsis transcriptional regulator DPB3‐1 enhances heat stress tolerance without growth retardation in rice
Source: Plant Biotechnol J. 2016 Feb 3;14(8):1756–67. doi: 10.1111/pbi.12535 (PMC5067654; doi:10.1111/pbi.12535)
Supplement: Supplementary file 8 — Table S8 GO analysis of the genes up‐regulated in the DPB3‐1‐overexpressing plants under the heat stress condition. [file PBI-14-1756-s004.docx]

**Table S8** GO analysis of the genes upregulated in the *DPB3-1*-overexpressing plants under the heat stress condition.

| Term | Background frequency | Sample frequency | P-value |
| --- | --- | --- | --- |
| Cation transport (GO:0006812) | 529 | 11 | 4.68E-04 |
| Ion transport (GO:0006811) | 798 | 13 | 6.08E-04 |
| Response to heat (GO:0009408) | 122 | 6 | 2.04E-03 |
| Metal ion transport (GO:0030001) | 348 | 8 | 9.73E-03 |

GO analysis was performed using GO Term Enrichment tool on Gene Ontology Consortium (http://geneontology.org/). The terms that are significantly enriched compared with the entire *Oryza sativa* genes are listed (P < 0.01). The background frequency and sample frequency refer to the number of genes in the categories among the total *Oryza sativa* genes and upregulated genes in the *DPB3-1*-overexpressing plants, respectively.
