# Supplementary material for: The Arabidopsis transcriptional regulator DPB3‐1 enhances heat stress tolerance without growth retardation in rice
Source: Plant Biotechnol J. 2016 Feb 3;14(8):1756–67. doi: 10.1111/pbi.12535 (PMC5067654; doi:10.1111/pbi.12535)
Supplement: Supplementary file 9 — Table S9 GO analysis of the genes down‐regulated in the DPB3‐1‐overexpressing plants under the heat stress condition. [file PBI-14-1756-s005.docx]

**Table S9** GO analysis of the genes downregulated in the *DPB3-1*-overexpressing plants under the heat stress condition.

| Term | Background frequency | Sample frequency | P-value |
| --- | --- | --- | --- |
| Metabolic process (GO:0008152) | 16691 | 84 | 1.43E-15 |
| Biological_process (GO:0008150) | 20954 | 92 | 4.42E-14 |
| Primary metabolic process (GO:0044238) | 11238 | 53 | 3.87E-06 |
| Organic substance metabolic process (GO:0071704) | 11638 | 54 | 4.48E-06 |
| Carbohydrate metabolic process (GO:0005975) | 1383 | 16 | 7.48E-05 |
| Phosphorylation (GO:0016310) | 2440 | 20 | 3.82E-04 |
| Phosphate-containing compound metabolic process (GO:0006796) | 3375 | 23 | 1.10E-03 |
| Phosphorus metabolic process (GO:0006793) | 3401 | 23 | 1.26E-03 |
| Protein phosphorylation (GO:0006468) | 1949 | 17 | 1.44E-03 |
| Cellular metabolic process (GO:0044237) | 10346 | 43 | 8.34E-03 |

GO analysis was performed using GO Term Enrichment tool on Gene Ontology Consortium (http://geneontology.org/). The terms that are significantly enriched compared with the entire *Oryza sativa* genes are listed (P < 0.01). The background frequency and sample frequency refer to the number of genes in the categories among the total *Oryza sativa* genes and downregulated genes in the *DPB3-1*-overexpressing plants, respectively.
