# Supplementary material for: The Arabidopsis transcriptional regulator DPB3‐1 enhances heat stress tolerance without growth retardation in rice
Source: Plant Biotechnol J. 2016 Feb 3;14(8):1756–67. doi: 10.1111/pbi.12535 (PMC5067654; doi:10.1111/pbi.12535)
Supplement: Supplementary file 10 — Table S10 Overrepresentation analysis of DRE, CCAAT and HSE sequences in the promoters of the top 100 up‐regulated genes in Ubi:DPB3‐1 rice under the heat stress condition. [file PBI-14-1756-s012.docx]

**Table S10** Overrepresentation analysis of DRE, CCAAT and HSE sequences in the promoters of the top 100 upregulated genes in *Ubi:DPB3-1* rice under the heat stress condition.

| *cis* element | Sequence | Fold change | Z-score | P-value |
| --- | --- | --- | --- | --- |
| DRE | ACCGAC | 1.81 | 3.10540 | 0.00095 |
| DRE | GCCGAC | 1.35 | 1.35487 | 0.08773 |
| DRE | GTCGGT | 1.18 | 0.57578 | 0.28238 |
| DRE | GTCGGC | 1.00 | 0.01082 | 0.49568 |
| CCAAT | CCAAT | 1.11 | 1.07005 | 0.14230 |
| CCAAT | ATTGG | 1.00 | -0.00477 | 0.50190 |
| HSE | GAANNTTC | 1.12 | 0.71549 | 0.23715 |

The results of the frequency of each DRE (A/GCCGAC) motif were extracted from the overrepresentation analysis of all hexamer sequences shown in Figure S8b, and the frequency of CCAAT and HSE (GAAnnTTC) motifs in the promoter sequences encompassing 1000 bp before each transcriptional start site of the top 100 upregulated genes in *Ubi:DPB3-1* rice under the heat stress condition were compared with that in the 100 randomly selected promoters from the entire rice genome. Statistic analysis was performed similarly to the overrepresentation analysis of all hexamer sequences.
