# Supplementary material for: The Arabidopsis transcriptional regulator DPB3‐1 enhances heat stress tolerance without growth retardation in rice
Source: Plant Biotechnol J. 2016 Feb 3;14(8):1756–67. doi: 10.1111/pbi.12535 (PMC5067654; doi:10.1111/pbi.12535)
Supplement: Supplementary file 11 — Table S11 Sequences of primers used in this study. [file PBI-14-1756-s007.docx]

**Table S11** Sequences of primers used in this study.

| Target gene | Locus ID | Oligonucleotide name | Sequence (5' to 3') |
| --- | --- | --- | --- |
| Primers used for cloning of coding | | | |
| *AtDPB3-1* | At1g07980 | NFYC10_5'_BamHI | ATGGATCCATGGTGTCGTCAAAGAA |
|  |  | NFYC10_3'_NotI | ATGCGGCCGCTCAGCCTGCATCTGTCAT |
| *OsDREB2B2* | LOC_Os05g27930 | OsDREB2B_5'_EcoRI | TAGAATTCATGACGGTGGATCAGAGGACG |
|  |  | OsDREB2B_AP2_3'_BamHI | ATGGATCCGGCCAAAATTAGTGCGAGC |
|  |  | OsDREB2B_5'_ClaI | TAATCGATATGACGGTGGATCAGAGGACG |
|  |  | OsDREB2B_3'_XhoI_ns | ATCTCGAGTCCCAAGCCCTCAAAGAACTG |
| *GmDREB2A* | Glyma14g06080 | GmDREB2A_5'_EcoRI | GCGAATTCATGGGTGCTTATGATCAAGTTTC |
|  |  | GmDREB2A-AP2_3'_BamHI | ATGGATCCTTTGGGAAAATTGAGGCGTG |
|  |  | GmDREB2A_5'_ClaI | GCATCGATATGGGTGCTTATGATCAAGTTTC |
|  |  | GmDREB2A_3'_XhoI_ns | ATCTCGAGCTAGCCACCCTTCCTTGCTT |
| OsDPB3-2 | LOC_Os03g63530 | OsDPB3-2_5'_ClaI | ATATCGATATGGCCGGGAAGAAGAAGGCCC |
|  |  | OsDPB3-2_3'_XhoI_ns | ATCTCGAGTAATTGTGGTTGGTTCACTTGGCTG |
|  |  | OsDPB3-2_5'_XbaI | ATTCTAGAGATGGCCGGGAAGAAGAAGGCCCTAA |
|  |  | OsDPB3-2_3'_XhoI | CGCTCGAGTTATTGTGGTTGGTTCACTTGGCTG |
| Primers used for qRT-PCR | | | |
| OsDPB3-2 | LOC_Os03g63530 | OsDPB3-2_rt_5 | ATCAACAAGGCCACCGAGATATT |
|  |  | OsDPB3-2_rt_3 | GCACACTGCTGTTGAAAGGTTAT |
| *OsHsfA3* | LOC_Os02g32590 | OsHsfA3_rt_5 | GCTGCCAGAGAACATAGGACTT |
|  |  | OsHsfA3_rt_3 | CAAGTTCCTCCTGTGTGTCAAA |
| *OsHsfA2a* | LOC_Os03g53340 | OsHsfA2a_rt_5 | GCGTCCAGGAGAGTAACAGC |
|  |  | OsHsfA2a_rt_3 | GGGGCTGAGGTGATATATGCT |
| *OsHsfA7* | LOC_Os01g39020 | OsHsfA7_rt_5 | CCAATGTGCAATTTCCAGAATA |
|  |  | OsHsfA7_rt_3 | TCCATTCCAGTTTCAGGTAAGG |
| *OsHsfA9* | LOC_Os03g12370 | OsHsfA9_rt_5 | CCCTCCTTTGAGTGTTCAAGAT |
|  |  | OsHsfA9_rt_3 | TCAAGCTTCGGTAATGACATC |
| *HSP20* family gene | LOC_Os01g04370 | LOC_Os01g04370_rt_5 | GGTGAGGGAAGAAGTCATGTTT |
|  |  | LOC_Os01g04370_rt_3 | ACACCAGCAGCAGACCATACT |
| *HSP20* family gene | LOC_Os03g15960 | LOC_Os03g15960.1_rt_5 | TCTGTCGTGAAGGAGCAAATAA |
|  |  | LOC_Os03g15960.1_rt_3 | AACAACACACTGACCCAGTGAC |
| *HSP20* family gene | LOC_Os03g16020 | LOC_Os03g16020.1_rt_5 | TACTGGTGTTTTTGGTGTGCTC |
|  |  | LOC_Os03g16020.1_rt_3 | TGAGACAACAGGTTTTACCGTTT |
| *OsDREB2B1* | LOC_Os05g27930 | OsDREB2B1_rt_5 | TCCAGCCCGGAAGAAAATGT |
| *OsDREB2B2* | LOC_Os05g27930 | OsDREB2B2_rt_5 | CAGCCCGGAAGGAAAAAGCG |
|  |  | OsDREB2B2_rt_3 | GCTCCTGCTGATTGTTGAGC |
| *HSP20* family gene | LOC_Os02g48140 | LOC_Os02g48140_rt_5 | TAGTACATGTCAAGCCTACCCG |
|  |  | LOC_Os02g48140_rt_3 | AAGTGCACTCATGCGCCATA |
| *HSP20* family gene | LOC_Os03g14180 | LOC_Os03g14180_rt_5 | ATTGAAGCAAGCAATCAAGCGA |
|  |  | LOC_Os03g14180_rt_3 | GAACCTAAAAGCAGTGAGCTGG |
| *Chaperone protein* | LOC_Os03g31300 | LOC_Os03g31300_rt_5 | ATTTCAAGGACGAGGACAGCAT |
|  |  | LOC_Os03g31300_rt_3 | TGGAAAACGAGCTTTTGCTGAG |
| *MYB* family gene | LOC_Os05g37060 | LOC_Os05g37060_rt_5 | GCTATCTAAGCACCGGCATTTG |
|  |  | LOC_Os05g37060_rt_3 | TCTCTCACACACTCAGATTCGC |
| *Leucine zipper* family gene | LOC_Os02g43330 | LOC_Os02g43330_rt_5 | AGCTAGACGGGAGAGCAGATTA |
|  |  | LOC_Os02g43330_rt_3 | TGCATGTGTGGATTTGCATTGT |
| *bZIP* family gene | LOC_Os07g08420 | LOC_Os07g08420_rt_5 | ATGGCAGACATTGAAGCCCTAA |
|  |  | LOC_Os07g08420_rt_3 | ACATTGAGGGGAGATTGCATGT |
| *Cytokinin-O-glucosyltransferase* | LOC_Os10g09990 | LOC_Os10g09990_rt_5 | CAGATCCTGCCTTGTCAGTACA |
|  |  | LOC_Os10g09990_rt_3 | TGTAACAACCTAAATGTGCGCT |
| *Pyruvate, phosphate dikinase* | LOC_Os03g31750 | LOC_Os03g31750_rt_5 | AGAGGATGTTGGTGGCATGAAT |
|  |  | LOC_Os03g31750_rt_3 | CCACACAACATTTTCCCCATCC |
| *Thiamine pyrophosphate enzyme* | LOC_Os05g39310 | LOC_Os05g39310_rt_5 | ATGGAGCTTGCCCTGGTTG |
|  |  | LOC_Os05g39310_rt_3 | GGTTGATGACGGCGTTGGAG |
| *18S rRNA* |  | 18S rRNA_RT-PCR_F | AAACGGCTACCACATCCAAG |
|  |  | 18S rRNA_RT-PCR_R | CCTCCAATGGATCCTCGTTA |
