# Supplementary material for: The Arabidopsis transcriptional regulator DPB3‐1 enhances heat stress tolerance without growth retardation in rice
Source: Plant Biotechnol J. 2016 Feb 3;14(8):1756–67. doi: 10.1111/pbi.12535 (PMC5067654; doi:10.1111/pbi.12535)
Supplement: Supplementary file 12 — Figure S1 Phenotypes of the vector control and DPB3‐1‐overexpressing Arabidopsis under non‐stress conditions after ripening. Figure S2 Alignment of DREB2A, OsDREB2B2 and GmDREB2A;2. Figure S3 Additional phenotypic analysis of the DPB3‐1‐overexpressing rice under nonstress conditions. Figure S4 Plant phenotype and seed morphology of the DPB3‐1‐overexpressing rice after desiccation. Figure S5 Phylogenetic tree lf HSP20 family proteins in Arabidopsis thaliana and Oryza sativa based on amino acids sequences of the conserved domain. Figure S6 Proportions of abiotic stress‐repressive genes among the down‐regulated genes in the DPB3‐1‐overexpressing rice under heat stress condition or rice whole genome. Figure S7 Confirmation of down‐regulated gene expression in Ubi:DPB3‐1 identified by microarray analysis. Figure S8 Microarray analysis of up‐regulated or down‐regulated genes in Ubi:DPB3‐1 plants under heat stress conditions. Figure S9 Drought stress tolerance of the DPB3‐1‐overexpressing rice. [file PBI-14-1756-s008.pdf]

Supporting information

Additional figures

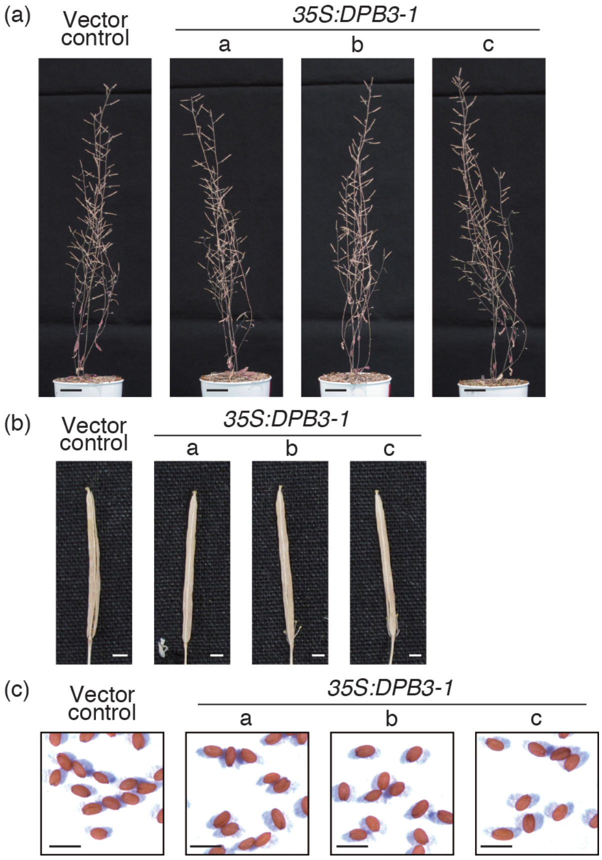

Figure S1 Phenotypes of the vector control and *DPB3-1*-overexpressing *Arabidopsis* under non-stress conditions after ripening. (a) Aerial parts of the vector control and *DPB3-1*-overexpressing *Arabidopsis* after ripening. Photographs were taken 60-d after sowing. Scale bars represent 1 cm. (b) Siliques of the vector control and *DPB3-1*-overexpressing *Arabidopsis* shown in (a) after ripening. Scale bars represent 1 mm. (c) Mature seeds of the vector control and *DPB3-1*-overexpressing *Arabidopsis* shown in (a). Scale bars represent 1 mm.

```
OsDREB2B2 :MTVDQRTTAKAIMPPVEMPPVQPGRKKRPRRSRDGPTSVAETIKRWAELNNQQELDPQGP  1-60
DREB2A    :MAVYDQSGDR------NRTQIDTSRKRKSRSRGDGTT-VAERLKRWKEYNETVE---EVS  1-50
GmDREB2A  :MGAYDQVSLK---------PLDSSRKRKSRSRGYGTGSVAETIAKWKEYNEHLYSGKDDS  1-51

OsDREB2B2 :KKARKAPAKGSKKGCMKGKGGPENTRCDFRGVRQRTWGKWVAEIREPNQQSRLWLGTFPT  61-120
DREB2A    :TKKRKVPAKGSKKGCMKGKGGPENSRCSFRGVRQRIWGKWVAEIREPNRGSRLWLGTFPT  51-110
GmDREB2A  :RTTRKAPAKGSKKGCMKGKGGPQNSQCNYRGVRQRTWGKWVGEIREPNRGSRLWLGTFSS  52-111

OsDREB2B2 :AEAAACAYDEAARAMYGPMARTNFG  121-145
DREB2A    :AQEAASAYDEAAKAMYGPLARLNFP  111-135
GmDREB2A  :AQEAALAYDEAARAMYGPCARLNFP  112-136
```

Figure S2 Alignment of DREB2A, OsDREB2B2 and GmDREB2A;2. The sequence of the interacting region of DREB2A with DPB3-1 (1-135 amino acids) was aligned with that of OsDREB2B2 and GmDREB2A;2. The numbers on the right side of the alignment correspond to the actual amino acid numbers of each protein.

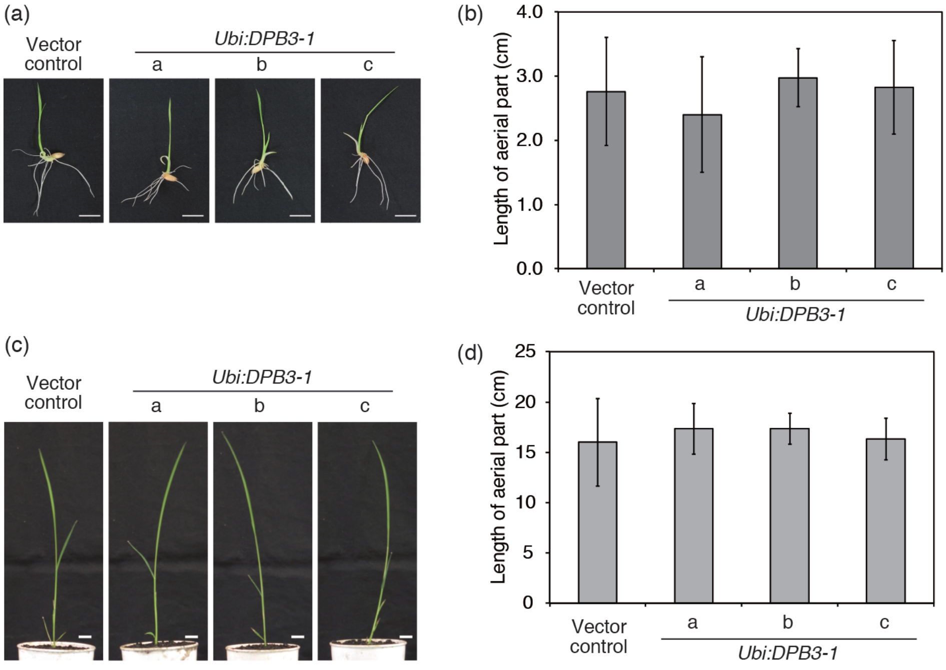

Figure S3 Additional phenotypic analysis of the *DPB3-1*-overexpressing rice under non-stress conditions. (a, c) Growth of the vector control and *DPB3-1*-overexpressing plants under normal conditions. Photographs of 7-d-old

(a) or 16-d-old (c) plants are shown. Scale bars represent 1 cm. (b, d) Average length of aerial part of the 7-d-old (b) or 16-d-old (d) vector control and *DPB3-1*-overexpressing plants calculated from the plants grown as shown in (a) or (c), respectively. The error bars indicated the SD (n = 12). The data were evaluated using one-way ANOVA, and no significant differences were detected (P > 0.05).

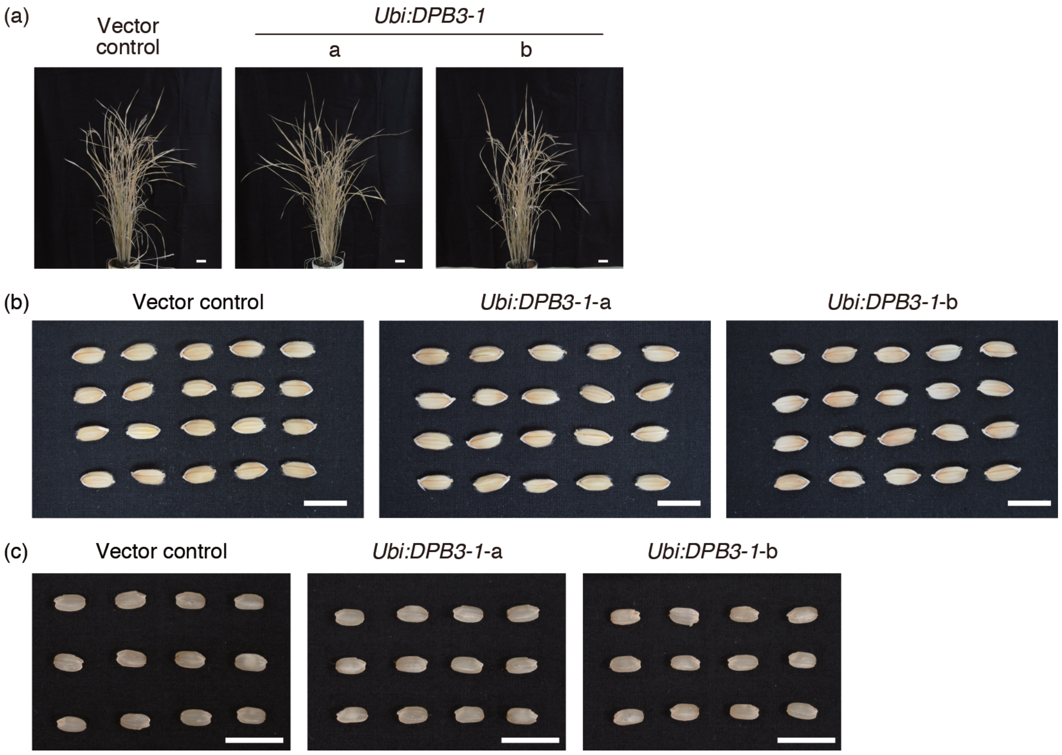

Figure S4 Plant phenotype and seed morphology of the *DPB3-1*-overexpressing rice after desiccation. (a) Phenotype of the vector control and *DPB3-1*-overexpressing plants after desiccation. The plants were grown under non-stress conditions and photographs were taken about 6 months after germination. Scale bars represent 5 cm. (b, c) Seed morphology of the vector control and *DPB3-1*-overexpressing rice. Photographs of rough rice grains (b) and hulled rice grains without gloms (c) are shown. Scale bars represent 1 cm.

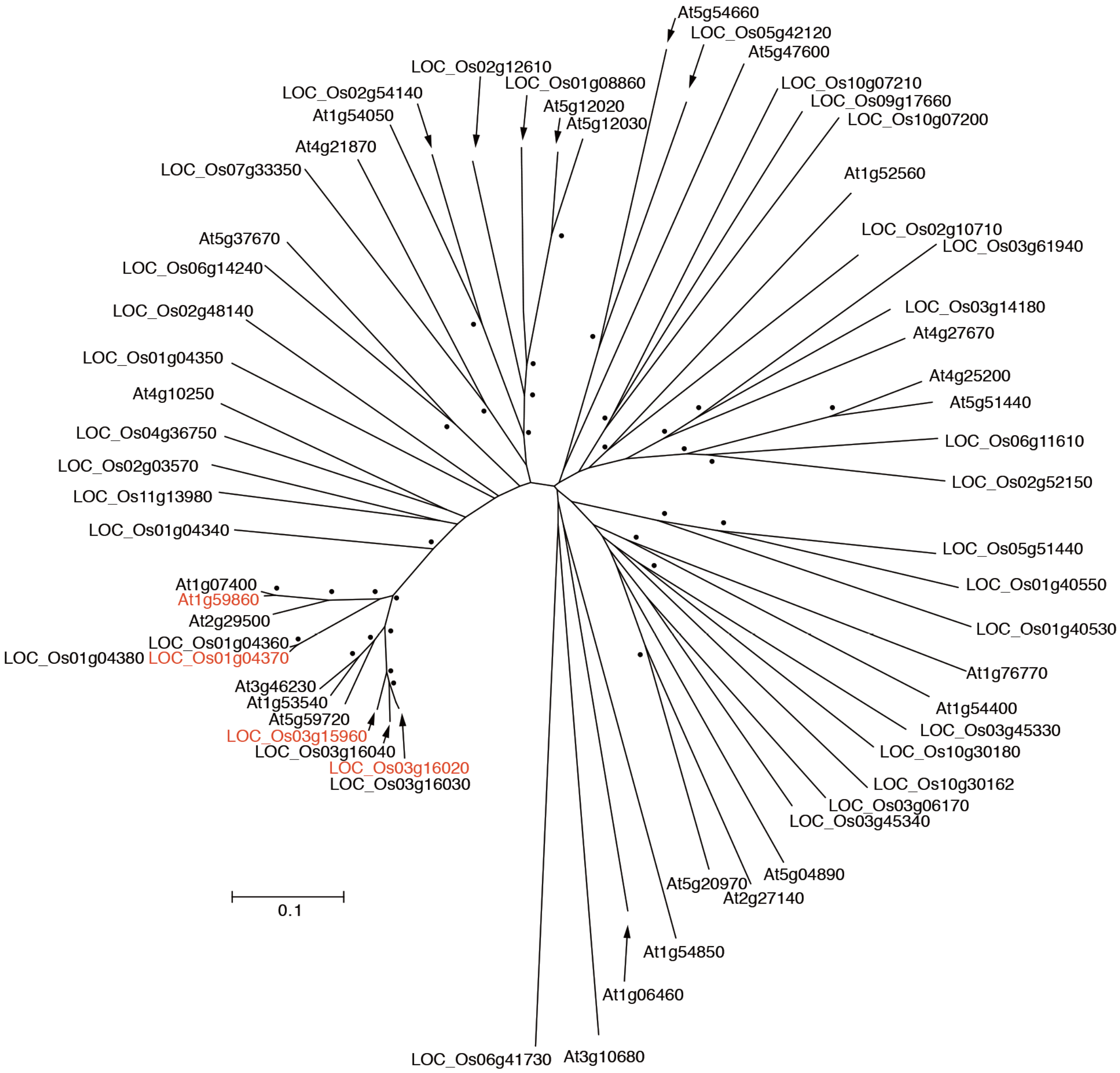

Figure S5 Phylogenetic tree If HSP20 family proteins in *Arabidopsis thaliana* and *Oryza sativa* based on amino acids sequences of the conserved domain. The peptide sequences of the HSP20 family proteins were obtained from Phytozome (Phytozome v10.1, http://www.phytozome.net/) according to the conserved domain (Pfam:00011). A consensus tree from 1000 bootstrap samplings is shown, and a dot indicates a node that was supported with a bootstrap value > 50. The scale bar indicates the substitution rate per residue. AtXgXXXXX and LOC_OsXXgXXXXX represent *Arabidopsis thaliana* and *Oryza sativa* family proteins, respectively. The candidate target gene of DPB3-1 in

Arabidopsis (At1g59860) and the highly conserved proteins in rice whose expression patterns were analyzed in this study were shown in red lettering.

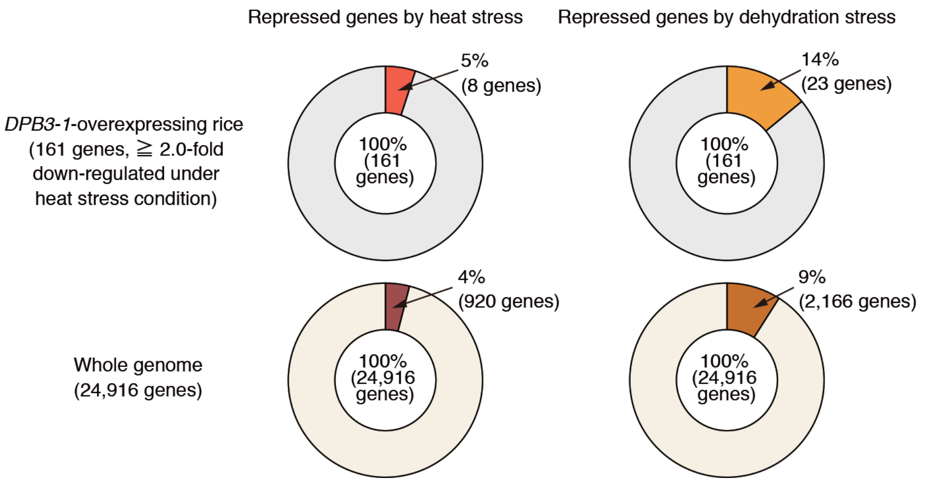

Figure S6 Proportions of abiotic stress-repressive genes among the downregulated genes in the *DPB3-1*-overexpressing rice under heat stress condition or rice whole genome.

Proportions of abiotic stress-inducible genes were calculated according to the microarray analysis (Table S3) and a previous paper (Maruyama et al., 2012; Venu et al., 2013). There were not the significant differences between the proportions of abiotic stress-inducible genes among the downregulated genes in the *DPB3-1*-overexpressing rice under heat stress condition or rice whole genome ($P > 0.05$, Fisher's exact test).

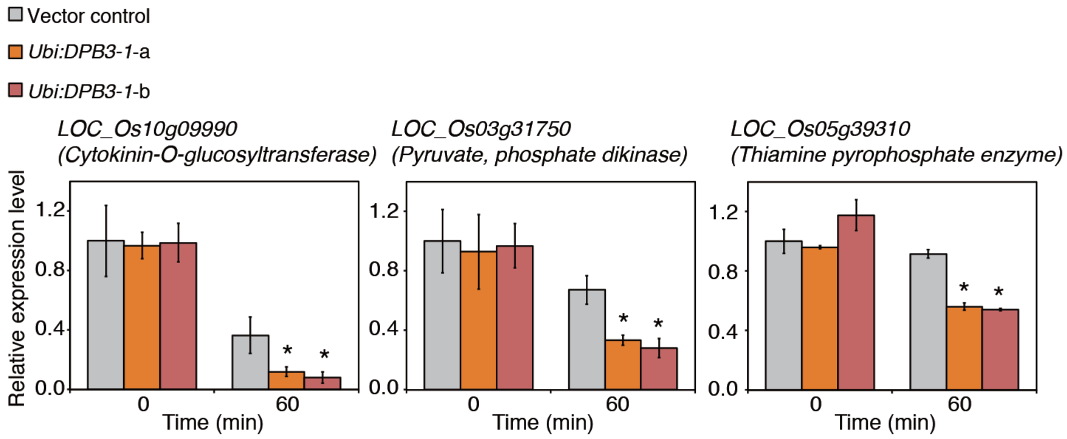

Figure S7 Confirmation of downregulated gene expression in *Ubi:DPB3-1* identified by microarray analysis. The expression levels of three downregulated genes by the overexpression of *DPB3-1* under heat stress condition were analyzed by quantitative RT-PCR analysis. The expression levels of each gene in the vector control plants were defined as 1.0. The error bars indicated the SD (n = 3). Asterisks indicate significant differences between the plants at each time point ($P < 0.05$ according to Bonferroni-corrected Student's *t* test).

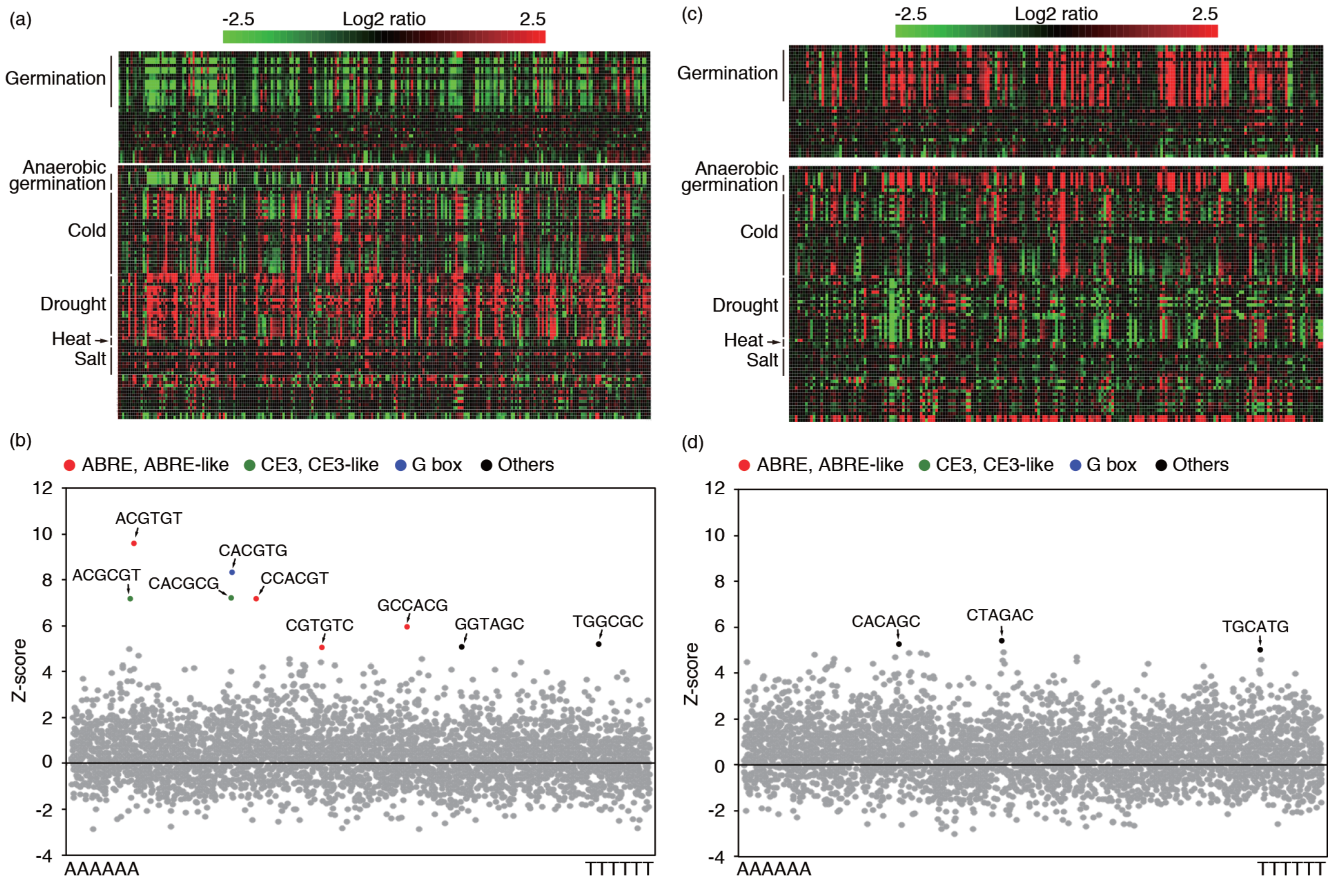

Figure S8 Microarray analysis of upregulated or downregulated genes in *Ubi:DPB3-1* plants under heat stress conditions. (a, c) Expression profile of the upregulated (a) or downregulated (c) genes in the *DPB3-1*-overexpressing plants under the heat stress condition. The top 100 upregulated genes (*x* axis, from left to right) under developmental conditions or in response to various abiotic stress (*y* axis) are shown as heat maps. (b, d) Overrepresentation analysis of hexamer sequences in the promoters of top 100 upregulated (b) or downregulated (d) genes in the *Ubi:DPB3-1* plants under the heat stress condition. Z-scores (*y* axes) for the observed frequencies of all hexamer sequences (*x* axes) are shown in the scatter plot. The highly conserved sequences of ABRE or ABRE-like (red), CE3 or CE3-like (green), G box (blue) and others (black) are exhibited (Z-scores > 5).

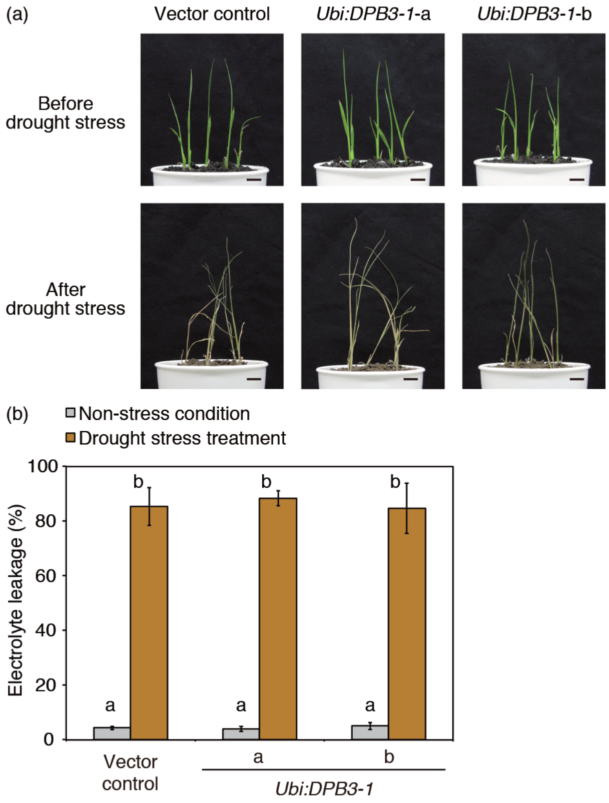

Figure S9 Drought stress tolerance of the *DPB3-1*-overexpressing rice. (a) Photographs of plants before and after drought stress treatment. The seeds were germinated in water for 7 days at 28°C, and after germination, the seedling were grown on soil for 4 days. After 4 days, water was withheld until wilting. Photographs were taken before stress treatment and 9 days after drought stress. Scale bars represent 1 cm. (b) Electrolyte leakage of plant with or without drought stress treatment. Plants shown in (a) were analyzed as the samples with drought stress treatment. The error bars indicated the SD ($n = 10$). There were no significant differences between plants with or without heat stress ($P > 0.05$ according to one-way ANOVA).

Additional tables

Table S1 Yield parameters of the vector control and *DPB3-1*-overexpressing *Arabidopsis* under non-stress conditions.

Traits	Vector control (%)	*35S:DPB3-1*-a (%)	*35S:DPB3-1*-b (%)	*35S:DPB3-1*-c (%)
Silique number per plant	107 ± 7 (100)	108 ± 9 (101)	102 ± 9 (95)	103 ± 5 (97)
Seed weight per plant (mg)	46.5 ± 5.3 (100)	43.2 ± 5.3 (93)	52.2 ± 4.1 (112)	56.6 ± 5.3 (121)
Silique length (mm)	11.1 ± 1.4 (100)	10.5 ± 1.3 (95)	10.3 ± 1.1 (93)	10.4 ± 1.1 (94)
Seed number per silique	43 ± 5 (100)	40 ± 5 (93)	43 ± 5 (100)	40 ± 2 (93)
Seed weight per 1000 seeds (mg)	15.1 ± 1.4 (100)	14.6 ± 1.5 (96)	15.8 ± 1.5 (104)	16.1 ± 0.4 (106)

Various parameters involved in the yield were measured. Values are the means and SD (n = 15). Relative percentages are shown in brackets. The data were evaluated using one-way ANOVA, and no significant differences were detected ($P > 0.05$).

Table S2 Number of *cis* elements on the promoters of genes which expression levels were analyzed in Figure 6.

Gene code	Annotation	DRE	CCAAT	HSE	ABRE
LOC_Os02g32590	OsHsfA3	1	0	0	1
LOC_Os03g53340	OsHsfA2a	0	2	5	1
LOC_Os01g39020	OsHsfA7	0	2	0	1
LOC_Os03g12370	OsHsfA9	4	2	0	1
LOC_Os01g04370	HSP20 family	0	1	1	1
LOC_Os03g15960	HSP20 family	3	3	1	0
LOC_Os03g16020	HSP20 family	0	1	2	2
LOC_Os05g27930	OsDREB2B	1	2	2	0

The sequences of 1-kb promoters of each gene were obtained from Phytozome (Phytozome v10.1, http://www.phytozome.net/). The numbers of DRE (A/GCCGAC), CCAAT, HSE (GAAnnTTC; “n” means an arbitrary nucleotide)

and ABRE (ACGTGG/T) on the 1-kb promoters are shown.

Table S3 Upregulated genes in the vector control rice under the heat stress condition.

This table is uploaded as an individual file because of the size.

Table S4 Downregulated genes in the vector control rice under the heat stress condition.

This table is uploaded as an individual file because of the size.

Table S5 Downregulated gene in *DPB3-1*-overexpressing rice under the non-stress condition.

MSU7_locus	Fold Change	Q-Value	Average (log2)	SD (log2)	Regulation	Description[a)]
LOC_Os05g08910	3.8	0.04004	-1.9	0.1	down	expressed protein

[a)] Description as given by the MSU 7.0 database.

Table S6 Upregulated gene in *DPB3-1*-overexpressing rice under the heat stress condition.

This table is uploaded as an individual file because of the size.

Table S7 Downregulated gene in *DPB3-1*-overexpressing rice under the heat stress condition.

This table is uploaded as an individual file because of the size.

Table S8 GO analysis of the genes upregulated in the *DPB3-1*-overexpressing plants under the heat stress condition.

Term	Background frequency	Sample frequency	P-value
Cation transport (GO:0006812)	529	11	4.68E-04
Ion transport (GO:0006811)	798	13	6.08E-04
Response to heat (GO:0009408)	122	6	2.04E-03
Metal ion transport (GO:0030001)	348	8	9.73E-03

GO analysis was performed using GO Term Enrichment tool on Gene Ontology Consortium (http://geneontology.org/). The terms that are significantly enriched compared with the entire *Oryza sativa* genes are listed (P < 0.01). The background frequency and sample frequency refer to the number of genes in the categories among the total *Oryza sativa* genes and upregulated genes in the *DPB3-1*-overexpressing plants, respectively.

Table S9 GO analysis of the genes downregulated in the *DPB3-1*-overexpressing plants under the heat stress condition.

Term	Background frequency	Sample frequency	P-value
Metabolic process (GO:0008152)	16691	84	1.43E-15
Biological_process (GO:0008150)	20954	92	4.42E-14
Primary metabolic process (GO:0044238)	11238	53	3.87E-06
Organic substance metabolic process (GO:0071704)	11638	54	4.48E-06
Carbohydrate metabolic process (GO:0005975)	1383	16	7.48E-05
Phosphorylation (GO:0016310)	2440	20	3.82E-04
Phosphate-containing compound metabolic process (GO:0006796)	3375	23	1.10E-03
Phosphorus metabolic process (GO:0006793)	3401	23	1.26E-03
Protein phosphorylation (GO:0006468)	1949	17	1.44E-03
Cellular metabolic process (GO:0044237)	10346	43	8.34E-03

GO analysis was performed using GO Term Enrichment tool on Gene Ontology Consortium (http://geneontology.org/). The terms that are significantly enriched

compared with the entire *Oryza sativa* genes are listed ($P < 0.01$). The background frequency and sample frequency refer to the number of genes in the categories among the total *Oryza sativa* genes and downregulated genes in the *DPB3-1*-overexpressing plants, respectively.

Table S10 Overrepresentation analysis of DRE, CCAAT and HSE sequences in the promoters of the top 100 upregulated genes in *Ubi:DPB3-1* rice under the heat stress condition.

cis element	Sequence	Fold change	Z-score	P-value
DRE	ACCGAC	1.81	3.10540	0.00095
DRE	GCCGAC	1.35	1.35487	0.08773
DRE	GTCGGT	1.18	0.57578	0.28238
DRE	GTCGGC	1.00	0.01082	0.49568
CCAAT	CCAAT	1.11	1.07005	0.14230
CCAAT	ATTGG	1.00	-0.00477	0.50190
HSE	GAANNTTC	1.12	0.71549	0.23715

The results of the frequency of each DRE (A/GCCGAC) motif were extracted from the overrepresentation analysis of all hexamer sequences shown in Figure S8b, and the frequency of CCAAT and HSE (GAAnnTTC) motifs in the promoter sequences encompassing 1000 bp before each transcriptional start site of the top 100 upregulated genes in *Ubi:DPB3-1* rice under the heat stress condition were compared with that in the 100 randomly selected promoters from the entire rice genome. Statistic analysis was performed similarly to the overrepresentation analysis of all hexamer sequences.

Table S11 Sequences of primers used in this study.

Target gene	Locus ID	Oligonucleotide name	Sequence (5' to 3')
		Primers used for cloning of coding	
AtDPB3-1	At1g07980	NFYC10_5'_BamHI	ATGGATCCATGGTGTCGTCAAAGAA
		NFYC10_3'_NotI	ATGCGGCCGCTCAGCCTGCATCTGTCAT
OsDREB2B2	LOC_Os05g27930	OsDREB2B_5'_EcoRI	TAGAATTCATGACGGTGGATCAGAGGACG
		OsDREB2B_AP2_3'_BamHI	ATGGATCCGGCCAAAATTAGTGCGAGC
		OsDREB2B_5'_ClaI	TAATCGATATGACGGTGGATCAGAGGACG
		OsDREB2B_3'_XhoI_ns	ATCTCGAGTCCCAAGCCCTCAAAGAACTG
GmDREB2A	Glyma14g06080	GmDREB2A_5'_EcoRI	GCGAATTCATGGGTGCTTATGATCAAGTTTC
		GmDREB2A-AP2_3'_BamHI	ATGGATCCTTTGGGAAAATTGAGGCGTG
		GmDREB2A_5'_ClaI	GCATCGATATGGGTGCTTATGATCAAGTTTC
		GmDREB2A_3'_XhoI_ns	ATCTCGAGCTAGCCACCCTTCCTTGCTT
OsDPB3-2	LOC_Os03g63530	OsDPB3-2_5'_ClaI	ATATCGATATGGCCGGGAAGAAGAAGGCCC
		OsDPB3-2_3'_XhoI_ns	ATCTCGAGTAATTGTGGTTGGTTCACTTGGCTG
		OsDPB3-2_5'_XbaI	ATTCTAGAGATGGCCGGGAAGAAGAAGGCCCTAA
		OsDPB3-2_3'_XhoI	CGCTCGAGTTATTGTGGTTGGTTCACTTGGCTG
		Primers used for qRT-PCR	
OsDPB3-2	LOC_Os03g63530	OsDPB3-2_rt_5	ATCAACAAGGCCACCGAGATATT
		OsDPB3-2_rt_3	GCACACTGCTGTTGAAAGGTTAT
OsHsfA3	LOC_Os02g32590	OsHsfA3_rt_5	GCTGCCAGAGAACATAGGACTT
		OsHsfA3_rt_3	CAAGTTCCTCCTGTGTGTCAAA
OsHsfA2a	LOC_Os03g53340	OsHsfA2a_rt_5	GCGTCCAGGAGAGTAACAGC
		OsHsfA2a_rt_3	GGGGCTGAGGTGATATATGCT
OsHsfA7	LOC_Os01g39020	OsHsfA7_rt_5	CCAATGTGCAATTTCCAGAATA
		OsHsfA7_rt_3	TCCATTCCAGTTTCAGGTAAGG
OsHsfA9	LOC_Os03g12370	OsHsfA9_rt_5	CCCTCCTTTGAGTGTTCAAGAT
		OsHsfA9_rt_3	TCAAGCTTCGGTAATGACATC
HSP20 family gene	LOC_Os01g04370	LOC_Os01g04370_rt_5	GGTGAGGGAAGAAGTCATGTTT
		LOC_Os01g04370_rt_3	ACACCAGCAGCAGACCATACT
HSP20 family gene	LOC_Os03g15960	LOC_Os03g15960.1_rt_5	TCTGTCGTGAAGGAGCAAATAA
		LOC_Os03g15960.1_rt_3	AACAACACACTGACCCAGTGAC
HSP20 family gene	LOC_Os03g16020	LOC_Os03g16020.1_rt_5	TACTGGTGTTTTTGGTGTGCTC

		LOC_Os03g16020.1_rt_3	TGAGACAACAGGTTTTACCGTTT
OsDREB2B1	LOC_Os05g27930	OsDREB2B1_rt_5	TCCAGCCCGGAAGAAAATGT
OsDREB2B2	LOC_Os05g27930	OsDREB2B2_rt_5	CAGCCCGGAAGGAAAAAGCG
		OsDREB2B2_rt_3	GCTCCTGCTGATTGTTGAGC
HSP20 family gene	LOC_Os02g48140	LOC_Os02g48140_rt_5	TAGTACATGTCAAGCCTACCCG
		LOC_Os02g48140_rt_3	AAGTGCACTCATGCGCCATA
HSP20 family gene	LOC_Os03g14180	LOC_Os03g14180_rt_5	ATTGAAGCAAGCAATCAAGCGA
		LOC_Os03g14180_rt_3	GAACCTAAAAGCAGTGAGCTGG
Chaperone protein	LOC_Os03g31300	LOC_Os03g31300_rt_5	ATTTCAAGGACGAGGACAGCAT
		LOC_Os03g31300_rt_3	TGGAAAACGAGCTTTTGCTGAG
MYB family gene	LOC_Os05g37060	LOC_Os05g37060_rt_5	GCTATCTAAGCACCGGCATTTG
		LOC_Os05g37060_rt_3	TCTCTCACACACTCAGATTCGC
Leucine zipper family gene	LOC_Os02g43330	LOC_Os02g43330_rt_5	AGCTAGACGGGAGAGCAGATTA
		LOC_Os02g43330_rt_3	TGCATGTGTGGATTTGCATTGT
bZIP family gene	LOC_Os07g08420	LOC_Os07g08420_rt_5	ATGGCAGACATTGAAGCCCTAA
		LOC_Os07g08420_rt_3	ACATTGAGGGGAGATTGCATGT
Cytokinin-O-glucosyltransferase	LOC_Os10g09990	LOC_Os10g09990_rt_5	CAGATCCTGCCTTGTCAGTACA
		LOC_Os10g09990_rt_3	TGTAACAACCTAAATGTGCGCT
Pyruvate, phosphate dikinase	LOC_Os03g31750	LOC_Os03g31750_rt_5	AGAGGATGTTGGTGGCATGAAT
		LOC_Os03g31750_rt_3	CCACACAACATTTTCCCCATCC
Thiamine pyrophosphate enzyme	LOC_Os05g39310	LOC_Os05g39310_rt_5	ATGGAGCTTGCCCTGGTTG
		LOC_Os05g39310_rt_3	GGTTGATGACGGCGTTGGAG
18S rRNA		18S rRNA_RT-PCR_F	AAACGGCTACCACATCCAAG
		18S rRNA_RT-PCR_R	CCTCCAATGGATCCTCGTTA

Procedures of supporting experiments

Plant material and growth conditions of *Arabidopsis thaliana*

Arabidopsis thaliana ecotype Columbia plants were grown under control condition as previously described (Sato et al., 2014). The transgenic *Arabidopsis* overexpressing *AtDPB3-1* was used as described in the paper.

Sequence alignment and phylogenic analysis

The peptide sequences of each protein were obtained from Phytozome (Phytozome v10.1, http://www.phytozome.net/). Alignment of the family proteins and construction of the neighbor-joining phylogenetic tree performed as described previously (Sato et al., 2014).

Analysis of microarray data

Meta-profile analysis of microarray data was performed using the public microarray database Genevestigator (Hruz et al., 2008). Overrepresentation analysis of hexamers in the promoters of up- or down-regulated genes was performed as described previously (Maruyama et al., 2012) using 1-kb upstream sequences from the translational start sites.

Drought stress treatment

The seeds were germinated in water for 7 days at 28°C, and the seedling were grown on soil for 4 days. After 4 days, water was withheld until wilting for 9 to 11 days.

Construct generation

For yeast two-hybrid assays, fragments of *OsDREB2B2* and *GmDREB2A;2* coding sequence were cloned into *Eco*RI and *Bam*HI sites of the pGBKT7 vector (Clontech). The pGADT7 vector harboring the *DPB3-1* coding sequence was used previously (Sato et al., 2014).

For GFP fluorescence observation, *OsDPB3-2* coding sequence was cloned into *Xba*I and *Xho*I sites of the pGKX-NsGFP vector (Qin et al., 2008).

For BiFC assays, coding sequences of *OsDREB2B2* and *GmDREB2A;2* were cloned into *Cla*I and *Xho*I sites of the pUCSPYCE vector (Qin et al., 2008), and coding sequence to *OsDPB3-2* was cloned into *Cla*I and *Xho*I sites of pUCSPYNE vector (Qin et al., 2008). The pUCSPYCE and pGKX-NsGFP vector harboring the *DPB3-1* coding sequence was used previously (Sato et al., 2014).

For transactivation assays, *OsDPB3-2* coding sequence was cloned into *Xba*I and *Xho*I sites of the pGKX vector (Qin et al., 2008). Other constructs used for transactivation assays were described in previous papers (Matsukura et al., 2010; Mizoi et al., 2013; Sato et al., 2014).

To generate the *Ubi:DPB3-1* constructs, the *DPB3-1* coding sequence was inserted into the *Bam*HI and *Not*I sites of the pGHU vector (Matsukura et al., 2010).

Supporting reference

Hruz, T., Laule, O., Szabo, G., Wessendorp, F., Bleuler, S., Oertle, L., Widmayer, P., Gruissem, W. and Zimmermann, P. (2008) Genevestigator v3: a reference expression database for the meta-analysis of transcriptomes. *Adv Bioinformatics* **2008**, 420747.

Maruyama, K., Todaka, D., Mizoi, J., Yoshida, T., Kidokoro, S., Matsukura, S., Takasaki, H., Sakurai, T., Yamamoto, Y.Y., Yoshiwara, K., Kojima, M., Sakakibara, H., Shinozaki, K. and Yamaguchi-Shinozaki, K. (2012) Identification of cis-acting promoter elements in cold- and dehydration-induced transcriptional pathways in Arabidopsis, rice, and soybean. *DNA Res* **19**, 37-49.

Matsukura, S., Mizoi, J., Yoshida, T., Todaka, D., Ito, Y., Maruyama, K., Shinozaki, K. and Yamaguchi-Shinozaki, K. (2010) Comprehensive analysis of rice DREB2-type genes that encode transcription factors involved in the expression of abiotic stress-responsive genes. *Mol Genet Genomics* **283**, 185-196.

Mizoi, J., Ohori, T., Moriwaki, T., Kidokoro, S., Todaka, D., Maruyama, K., Kusakabe, K., Osakabe, Y., Shinozaki, K. and Yamaguchi-Shinozaki, K. (2013) GmDREB2A;2, a canonical DEHYDRATION-RESPONSIVE ELEMENT-BINDING PROTEIN2-type transcription factor in soybean, is posttranslationally regulated and mediates dehydration-responsive element-dependent gene expression. *Plant Physiol* **161**, 346-361.

Qin, F., Sakuma, Y., Tran, L.S., Maruyama, K., Kidokoro, S., Fujita, Y., Fujita, M., Umezawa, T., Sawano, Y., Miyazono, K., Tanokura, M., Shinozaki, K. and Yamaguchi-Shinozaki, K. (2008) Arabidopsis DREB2A-interacting proteins function as RING E3 ligases and negatively regulate plant drought stress-responsive gene expression. *Plant Cell* **20**, 1693-1707.

Sato, H., Mizoi, J., Tanaka, H., Maruyama, K., Qin, F., Osakabe, Y., Morimoto, K., Ohori, T., Kusakabe, K., Nagata, M., Shinozaki, K. and Yamaguchi-Shinozaki, K. (2014) Arabidopsis DPB3-1, a DREB2A interactor, specifically enhances heat stress-induced gene expression by forming a heat stress-specific transcriptional complex with NF-Y subunits.

Plant Cell **26**, 4954-4973.

Venu, R.-C., Sreerekha, M.V., Madhav, M.S., Nobuta, K., Mohan, K.M., Chen, S., Jia, Y., Meyers, B.C. and Wang, G.-L. (2013) Deep transcriptome sequencing reveals the expression of key functional and regulatory genes involved in the abiotic stress signaling pathways in rice. *Journal of Plant Biology* **56**, 216-231.
